# The relationship between the Early Childhood Environment Rating Scale and its revised form and child outcomes: A systematic review and meta-analysis

**DOI:** 10.1371/journal.pone.0178512

**Published:** 2017-06-06

**Authors:** Ashley Brunsek, Michal Perlman, Olesya Falenchuk, Evelyn McMullen, Brooke Fletcher, Prakesh S. Shah

**Affiliations:** 1 Applied Psychology and Human Development, University of Toronto/OISE, Toronto, Ontario, Canada; 2 Department of Gastroenterology, Alberta Children's Hospital, Calgary, Alberta, Canada; 3 Department of Pediatrics, Mount Sinai Hospital, Toronto, Ontario, Canada; 4 Department of Pediatrics, University of Toronto, Toronto, Ontario, Canada; 5 Institute of Health Policy, Management and Evaluation, University of Toronto, Toronto, Ontario, Canada; Kyoto University, JAPAN

## Abstract

The Early Childhood Environment Rating Scale (ECERS) and its revised version (ECERS-R) were designed as global measures of quality that assess structural and process aspects of Early Childhood Education and Care (ECEC) programs. Despite frequent use of the ECERS/ECERS-R in research and applied settings, associations between it and child outcomes have not been systematically reviewed. The objective of this research was to evaluate the association between the ECERS/ECERS-R and children’s wellbeing. Searches of Medline, PsycINFO, ERIC, websites of large datasets and reference sections of all retrieved articles were completed up to July 3, 2015. Eligible studies provided a statistical link between the ECERS/ECERS-R and child outcomes for preschool-aged children in ECEC programs. Of the 823 studies selected for full review, 73 were included in the systematic review and 16 were meta-analyzed. The combined sample across all eligible studies consisted of 33, 318 preschool-aged children. Qualitative systematic review results revealed that ECERS/ECERS-R total scores were more generally associated with positive outcomes than subscales or factors. Seventeen separate meta-analyses were conducted to assess the strength of association between the ECERS/ECERS-R and measures that assessed children’s language, math and social-emotional outcomes. Meta-analyses revealed a small number of weak effects (in the expected direction) between the ECERS/ECERS-R total score and children’s language and positive behavior outcomes. The Language-Reasoning subscale was weakly related to a language outcome. The enormous heterogeneity in how studies operationalized the ECERS/ECERS-R, the outcomes measured and statistics reported limited our ability to meta-analyze many studies. Greater consistency in study methodology is needed in this area of research. Despite these methodological challenges, the ECERS/ECERS-R does appear to capture aspects of quality that are important for children’s wellbeing; however, the strength of association is weak.

## Introduction

A high proportion of children in Canada [1] and the US [2] receive care from someone other than their parents. As a result, increasing attention is being paid to the role of Early Childhood Education and Care (ECEC) programs in fostering optimal child development. Children who attend ECEC programs of higher quality demonstrate better cognitive [3,4], social [5,6] and emotional [7] outcomes. However, ECEC program quality in the US has been reported to be “mediocre” at best [8–11]. In addition, more information is needed about the validity of measures of ECEC quality [12]. The Early Childhood Environment Rating Scale (ECERS, and its revised version, the ECERS-R) is the most widely used assessment of global childcare classroom quality in centre-based programs [13–15]. It was developed to reflect the early childhood education field’s concept of Developmentally Appropriate Practice (DAP), an approach to teaching grounded in research on how young children learn and in what is known about effective early education [16]. As a result, items on the ECERS/ECERS-R assess a variety of aspects that influence the classroom environment, including curriculum, environment, teacher-child interactions, and teaching practices. The ECERS/ECERS-R is often used for research purposes or as a self-assessment tool to guide quality improvement efforts led by licensing or other agencies. Perhaps even more importantly, it is frequently used in high-stake settings such as Quality Rating and Improvement Systems (QRIS) [17]. In fact, over half of US states use the ECERS-R as part of their QRIS to monitor their state pre-K programs [17–19] and to assist in the allocation of public funding to programs [20].

ECEC quality has been conceptualized in terms of structural (e.g., staff/child ratios and aspects of the physical environment that can be regulated) and process (e.g., interactions that occur within the child’s environment) quality [21]. ECEC assessment tools tend to focus on one of these aspects, resulting in a narrower assessment of classroom quality. For example, the Caregiver Interaction Scale [22] focuses on the interaction styles of individual staff with the children under their care. A more recently developed measure, the Classroom Assessment Scoring System (CLASS) [23] assesses the quality of staff/child interactions at an aggregate, classroom level. A recent systematic review and meta-analysis revealed few associations between the CLASS and child outcomes [24]. The more global nature of the ECERS/ECERS-R may make it a more promising measure of ECEC quality in terms of possible associations with child outcomes.

The ECERS was created as a global measure of quality, designed to measure both structural and process aspects of ECEC environments [25]. A decade later, a revised version, the ECERS-R, was created to accommodate developments in the field related to cultural diversity, family involvement, and children with disabilities [26]. The ECERS and ECERS-R are made up of 37 and 43 items respectively. All items are rated on a seven-point scale. Both versions consist of the following seven subscales: 1) space and furnishings; 2) personal care routines; 3) language-reasoning; 4) activities; 5) interactions; 6) program structure and 7) parents and staff. However, a number of psychometric analyses suggest that the ECERS and ECERS-R are unidimensional measures of quality, providing a total score only [27–29]. Other studies have reported a two-factor solution of Appropriate Caregiving and Developmentally Appropriate Activities and Materials for the ECERS [30] and Teaching and Interactions and Provisions for Learning for the ECERS-R [14,31], grouping items into process and structural aspects of the environment.

Despite the ECERS/ECERS-R influential role in policy and research, to our knowledge, a comprehensive review that assesses whether or not the ECERS/ECERS-R is associated with child outcomes has yet to be published. Thus, the objective of this review was to evaluate the associations between ECERS/ECERS-R total scores in classrooms that serve preschool-aged children and children’s concurrent or subsequent outcomes.

We decided to cast a wide net regarding child outcomes in an attempt to capture cognitive, academic, social and emotional outcomes, all of which contribute to children’s wellbeing. Including this array of outcomes, particularly social emotional outcomes (e.g., positive and problem behavior) reflects an understanding of the classroom context having an impact on children that goes “beyond achievement tests” [32]. An examination of the associations between various subscales and factors and child outcomes was also conducted, with a specific focus on the Teaching and Interactions and Provisions for Learning factors and the Language-Reasoning and Interactions subscales, as these areas have been identified as particularly important for children’s development [5,33].

## Methods

### Types of participants and settings

We restricted our population to classrooms that serve preschool-aged children (age between 30 and 72 months) as these serve the largest number of children in ECEC settings [34,35]. Furthermore, different assessment measures are often required to capture the development of children of different age groups (e.g., infants, toddlers) making it extremely difficult to combine all age groups in one meta-analysis. ECEC settings included child care centers, preschool programs, nursery schools, pre-kindergarten programs, and Head Start programs. Studies that only examined home-based child care or those in which home- and center-based care could not be separated were excluded. The inclusion criteria and rationale are provided in Table 1.

**Table 1:** Inclusion criteria for systematic review and rationale.

Criteria	Rationale
***Child Care Type***	
Only studies that examined the impact of the quality of centre-based programs on children’s outcomes were included. Centre-based programs included daycare and preschool programs, nursery schools, pre-kindergarten programs, and Head Start programs. Studies that only examined home-based child care, or those in which home-based and centre-based could not be separated were excluded.	Center-based child care settings differ from home daycare in many ways such as ratios, group size, physical environment, curriculum, age range of children, and caregiver qualifications. As a result, quality is often measured differently for these two settings (e.g., ECERS versus FCCERS).
***Age Served***	
Studies that included preschool-aged children as the majority of participants were included. For the purposes of the meta-analysis, preschool-age was defined as ranging from 30 to 72 months.	Preschool-aged classrooms are different from infant/toddler classrooms due to the developmental stage and needs of the children in these two age groups. As a result, regulations and standards of care (e.g., ratios, physical environment, etc.) as well as daily activities (e.g., curriculum) differ between infant/toddler and preschool-aged classrooms.
***Child Outcomes***	
Studies that provided information about the association between ECERS/ECERS-R on children’s cognitive, academic, social-emotional, health, or motor outcomes were included. Data could have been gathered from teachers, parents, and/or children themselves. Measures that focus on dyads (e.g., attachment) were excluded.	Cognitive, academic, social-emotional, health, and motor outcomes were selected because they are key predictors of children’s developmental trajectories. Measures that focus on staff-child or peer dyads were not included given that these outcomes often reflect an aspect of child care quality.
***Study Design***	
Cross-sectional and longitudinal designs were included. When multiple child outcome assessments were reported the earliest time-point following the measurement of quality were extracted.	To increase the homogeneity across the extracted data from eligible studies (i.e., increase the likelihood of meta-analysis), we focused on the earliest time-point in which child outcomes were measured following the measurement of quality in instances where multiple waves of outcome data were presented.
***Outcome Reporting***	
Studies must have presented statistical data quantifying the association between ECERS/ECERS-R and a child outcome measure.	Studies only reporting qualitative results were not considered for this review as the domains of assessment could vary markedly between studies.
***Language***	
To be extracted studies had to be in English.	We did not have resources to systematically translate material written in other languages.

Abbreviations: ECERS = Early Childhood Environment Rating Scale; FCCERS Family Child Care Environment Rating Scale.

### Assessment of classroom quality

We used the ECERS/ECERS-R as a measure of classroom quality for this review. The components of this scale are described above. Though they have slightly different items, a high correlation between the ECERS and ECERS-R [14,20] has been reported and justifies combining the literature across the two versions. This allowed for a synthesis of information across a broader span of time and a larger number of studies. However, as part of this review we did set out to explore whether there is a different pattern of associations between the ECERS and ECERS-R and child outcomes.

### Types of studies

English language studies reporting associations in cohort, cross-sectional or longitudinal analyses were included in this review. Studies reporting a statistical link between an aggregate ECEC quality variable that consisted of several measures of quality and child outcomes were only included if the specific effect of ECERS/ECERS-R scores could be separated. The majority of the studies included were peer reviewed. Case-series, reviews, editorials and letters to editors were read to identify articles but were not included in the review.

### Outcomes

Child outcomes were operationalized broadly and included measures of children’s cognitive, pre-academic, social, emotional, behavioral, and motor functioning, all of which contribute to children’s overall wellbeing. Outcome measures were based on direct testing of children as well as teacher and parent reports. Measures that focused on dyads (e.g., staff/child attachment) were excluded, as it is difficult to separate “caregiver/program” effects from child characteristics using such measures.

### Search strategy

An extensive search of the electronic databases PsycINFO, Medline, and ERIC was conducted for English language studies published before July 3, 2015. Two separate searches were performed within each of the three databases. One combined search terms specific to ECERS/ECERS-R and child outcomes and the other combined search terms related to a number of ECEC quality indicators and child outcomes to capture studies in which the ECERS/ECERS-R was not the primary focus of the study. Specific keywords used in the electronic searches are provided in supplemental online material, Tables A-D in S1 File. Websites for key databases used in this literature were reviewed to retrieve relevant studies (e.g., Cost, Quality, and Outcomes Study [36]; Early Childhood Longitudinal Study [37]; Effective Provision of Pre-School Education (EPPSE) [38]; Head Start Impact Study (HS) [39]; National Center for Early Development and Learning’s (NCEDL) Multi-State Study of Pre-Kindergarten and State-Wide Early Education Program Study (SWEEP) [40]; Head Start’s Family and Child Experiences Survey (FACES) [41] and the National Institute of Child Health and Human Development’s (NICHD) Study of Early Child Care and Youth Development) [42]. Finally, reference lists of studies that met our inclusion criteria were manually searched to identify additional relevant studies.

### Study selection and data extraction

Decisions about what to search for and what to retain/exclude are key when conducting a systematic review and meta-analysis. In this study, we adopted a comprehensive approach in what we included as part of the systematic review but were more conservative in what we deemed meta-analyzable. This approach resulted in a comprehensive review that represents the existing literature without running the risk of combining studies that were too methodologically different.

The title and abstract of each paper located through the literature searches were reviewed for relevance. Abstracts that were identified as potentially relevant to the current study underwent full-text review to determine if inclusion criteria were met. Relevant child and family characteristics and reported measures of association were extracted using standardized forms. All reviews were conducted by two independent raters with a third member used for arbitration.

### Statistical analyses

All eligible studies were included in the systematic review. Data included the following statistics: zero order Pearson product-moment correlation coefficient (*r*), Beta, unstandardized coefficient, T-Test, partial correlations, F-Ratio, and various effect sizes. For meta-analyses, the Pearson product-moment correlation coefficient (*r*) or equivalent was used to assess the strength of the association between ECERS/ECERS-R scores and outcome measures (see S2 File). Studies that could be meta-analyzed were drawn from the pool of studies that were eligible for the systematic review. To be meta-analyzed, studies had to use identical operationalizations of the ECERS/ECERS-R and identical child outcome measures. Although there is no empirical basis for requiring a minimum number of studies to conduct a meta-analysis, we set three independent samples as our minimum. To increase homogeneity among studies that were meta-analyzed, and to ensure that children had at least some exposure to the program before outcomes were measured, only studies where a) the authors explicitly stated that children had been in the program for a minimal period of time prior to their assessment b) child pre-scores were available and could be used as a covariate or c) gain scores were provided, were included in meta-analyses. Sixteen of the 21 samples (in 17 studies) that met these inclusion criteria consisted of children who were assessed in the fall and spring, indicating children were in their classrooms for a minimum of 10 months [13,15,27,43–52]. Authors of the remaining studies reported that they ensured that children had exposure to the program prior to quality and child outcome assessments [53] for 2 to 24 months [13,24,54]. In addition, only statistics that accounted for covariates (e.g., child and family characteristics) were combined within a single meta-analysis. When different studies reported results based on the same samples, only the study with the largest sample was included in the meta-analysis [55]. Thus, only one coefficient from each sample was included in any one meta-analysis.

Statistical models with quadratic terms assume non-linear associations between the variables. Given that the statistics extracted for most studies only test for linear relationships (correlation coefficients and linear regression coefficients), associations in models using quadratic terms were excluded and only results examining linear relationships were used in the meta-analyses. We used random-effects models for meta-analyses. All meta-analyses were conducted using Comprehensive Meta-Analysis Version 3 software[56]. Statistical heterogeneity was calculated for each meta-analysis using the *I*^*2*^ values [57].

Efforts were made to rate the methodological quality of the studies based on existing measures of study quality (e.g., using the Newcastle-Ottawa scale [43]. However, this effort was deemed unhelpful, as there was little variability between studies, with studies generally being rated as being of poor quality due to the observational nature of this body of research.

## Results

### Search results

One of the strengths of this study is that the terms used in our initial searches were very broad. Casting a wide net reduced the likelihood that relevant studies were left out of this review. The downside of this strategy is that our searches included a large number of studies that were not relevant for this systematic review/meta-analysis and were therefore dropped during our systematic screening process.

**Fig 1:**
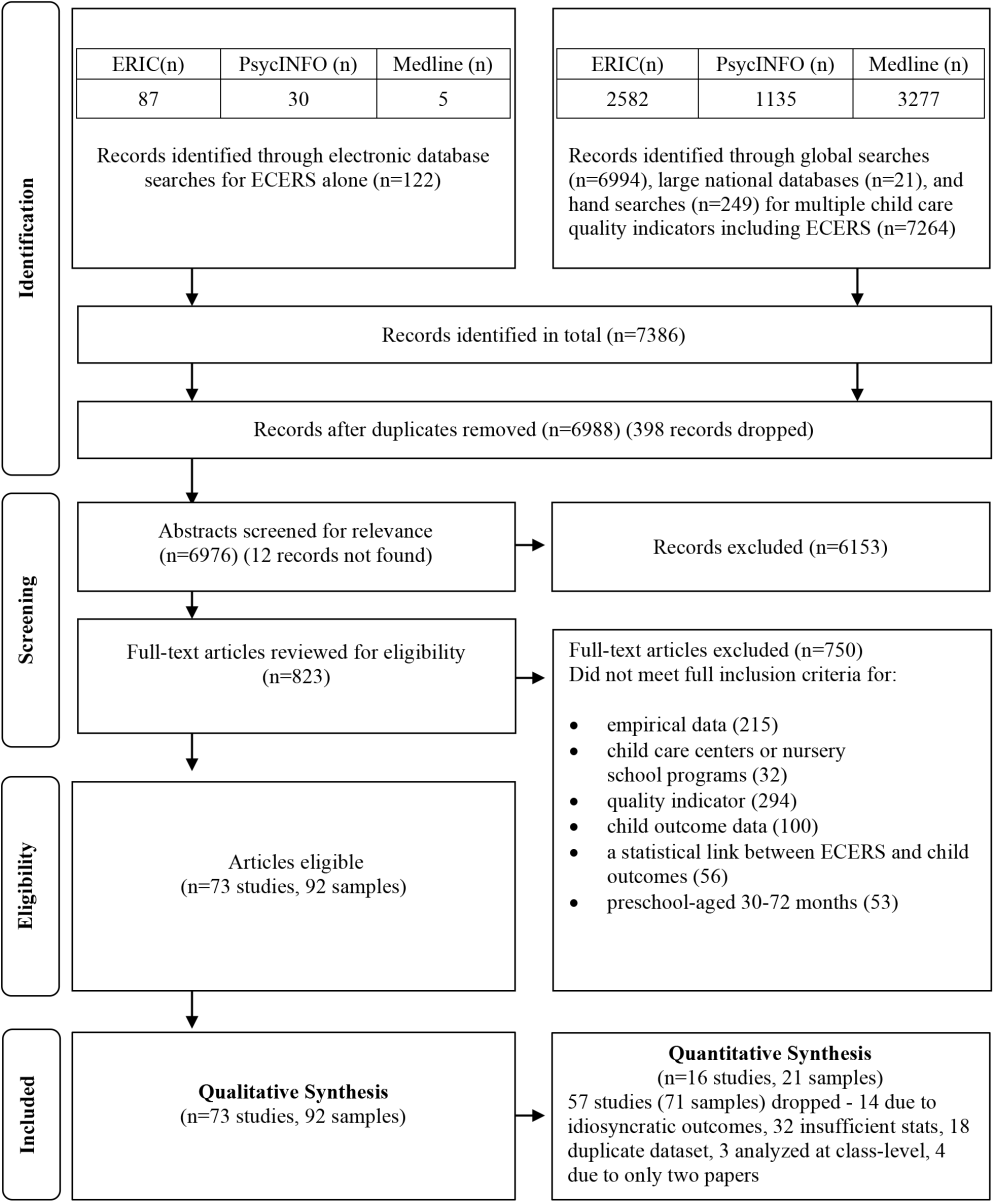
Flow diagram for study selection. **Adapted from Moher, 2009** [47]

Details of the search results and study selection are provided in Fig 1. Seventy-three studies were included in this review. There were 49 journal articles, 19 reports, one monograph, and four book chapters that reported original analyses. Descriptive information for the 73 studies is presented in S3 File. Several of these studies came from large-scale datasets with the largest number based on the NCEDL’s Multi-State Study and SWEEP study (*n* = 11) [15,33–35,44,58–63] and CQO (n = 5) [5,11,15,64,65] databases. Sixteen studies [7,45,46,54,66–77] included ECEC programs located outside of the United States (i.e., Bangladesh, Bermuda, Canada, Chile, England, Germany, Portugal, Singapore).

The 73 eligible studies produced 92 samples, as four studies [5,15,59,60] consisted of multiple datasets and seven studies [13,27,58,70,73,78,79] divided their sample into different groups of participants for analyses. Of the 92 samples, 23 samples (20 studies) measured ECERS/ECERS-R and outcomes using a cross-sectional design [5,7,15,35,45,64,66,68,69,71–74,77,78,80–83]. Sixty-nine samples (55 studies) [4,5,11,13,15,27,29,30,33,34,44,48–54,58–63,65,67,70,75,76,79,84–107]were from studies using a longitudinal design.

Fifty of the 73 studies reported the ethnic composition of their samples. Children were primarily Caucasian, Black or Hispanic. The majority of samples consisted of at-risk children with 18–100% of children coming from low-income families. Nineteen studies did not report children’s risk status. All of the studies had similar numbers of boys and girls. Of the independent samples, the total sample size across all eligible studies consisted of 33,318 preschool-aged children, ranging from 25 to 3584 children (median = 258).

### Operationalization of ECERS/ECERS-R

Of the 73 eligible studies, 26 [4,5,7,11,13,15,27,30,46,64,65,68–70,72–74,77,78,80,84,88,89,98–100] used the ECERS and 47 [15,29,33–35,44,45,48–54,58–61,63,66,67,71,75,76,79,81–83,85–87,90–97,101–108], used the ECERS-R. Some studies provided total and some provided average scores. Seven studies [4,7,13,64,69,70,84] using the ECERS and 15 studies [29,33,49,50,53,59–61,67,81,91,93,102,105,106] using the ECERS-R dropped the ‘Parents and Staff’ subscale from the reported total mean score.

One study [11] used the mean of 5 items, stating that this brief version was highly correlated with the complete ECERS scale. Another [58] dropped the ‘Toileting’ and ‘Parent and Staff’ subscales from the reported total score. Two studies reported the Preschool Appropriate Caregiving (PAC) factor [30,77] and 2 reported the Developmentally Appropriate Activities (DAA) factor [77,99] of the ECERS. Six studies [34,44,63,85,86,107] explored the Provisions for Learning (PL) factor and 10 [34,35,44,62,63,85,86,95,96,107] explored the Teaching and Interactions (TI) factor of the ECERS-R. A few studies explored individual subscales (5 using the ECERS and 6 using the ECERS-R), with Language-Reasoning [15,46,51,87,89,94,99–101] and Interactions [15,51,75] investigated most frequently. All studies reported that ECERS/ECERS-R were collected by trained observers.

### Outcomes

Across the studies that met our inclusion criteria, associations were reported between the ECERS/ECERS-R and 168 different outcomes (see S4 File for a complete list of outcome measures across all studies). Outcome measures varied substantially in terms of the skill/ability being assessed (e.g., inattention, receptive language, counting task), informant (e.g., child assessment, teacher or parent report), and psychometrics (e.g., standardized norm-referenced measures vs. tasks developed by authors with little or no reliability and validity data reported).

### Systematic review

#### ECERS/ECERS-R total score

**Fig 2:**
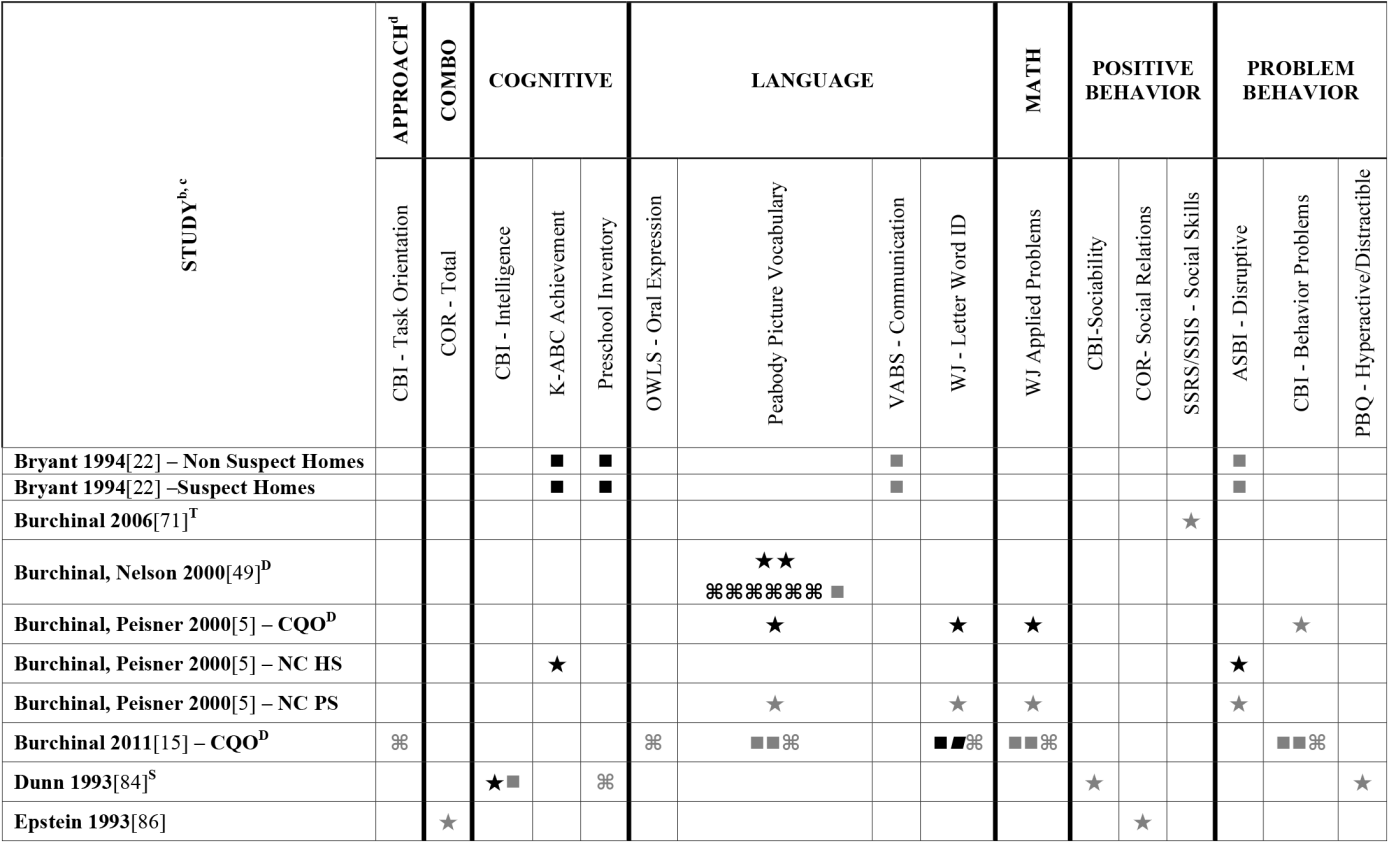
Systematic review of the associations between the ECERS total score, authors A-E and child outcomes. ^a^ Abbreviations: Symbols bolded are significant and positive, symbols bolded and italicized are significant and negative, and symbols in grey are non-significant. Star = Zero Order Pearson’s Correlation, Unfilled circle = Beta, Filled square = Unstandardized Coefficient, Black diamond minus white X = T-Test, Key clover = Partial Correlation, Downward arrow = Effect Size, Filled circle = F-Ratio. ^**a**^To improve the readability of these complex diagrams, ten papers[4,46,54,66,67,70,75,82,83,101] that had an outcome that appeared in only that one paper were omitted from this figure. Several analyses from other papers that had idiosyncratic outcomes are also excluded. For a comprehensive display of all of the data for all of the child outcomes see Supplemental Information S4 File.^**b**^This paper is one of a series of Meta-Analyses and Systematic Reviews assessing the relationship between child care quality and children’s outcomes; therefore, superscript letters below are in reference to various large databases that samples in these papers were drawn from. These letters have been kept consistent across the series for our readers. ^**c**^Samples within papers are described in more detail in S3 File. ^d^Acronyms for child outcomes are listed in S6 File. ^**D**^Cost, Quality and Outcomes Study (CQO, 1993–1994); ^**S**^8-county region of North-Central Indiana (Year NR); ^**T**^Otitis Media Study (Year NR); ^Z^Colorado QRIS.

**Fig 3:**
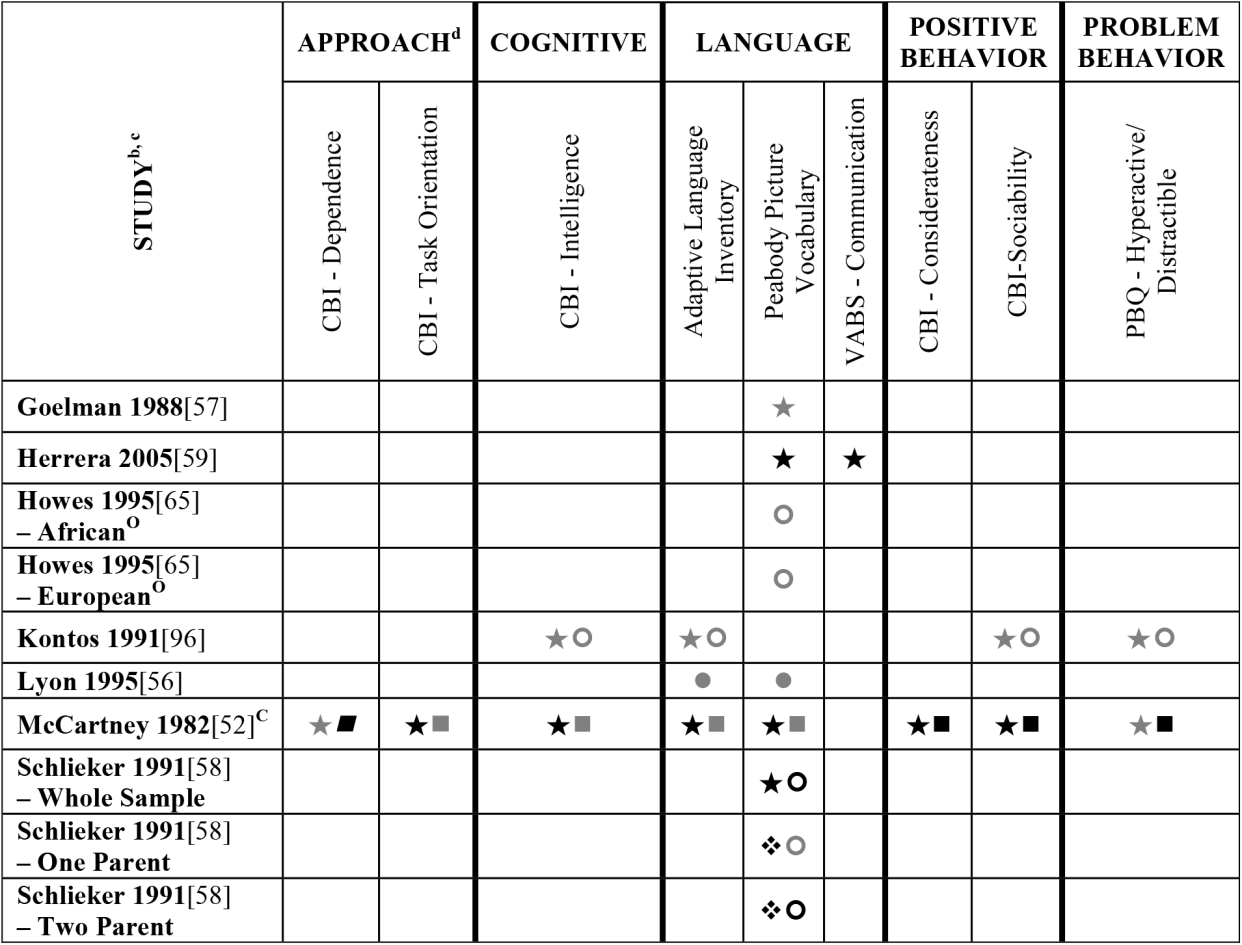
Systematic review of the associations between the ECERS total score, authors F-Z and child outcomes. ^a^Abbreviations: Symbols bolded are significant and positive, symbols bolded and italicized are significant and negative, and symbols in grey are non-significant. Star = Zero Order Pearson’s Correlation, Unfilled circle = Beta, Filled square = Unstandardized Coefficient, Black diamond minus white X = T-Test, Key clover = Partial Correlation, Downward arrow = Effect Size, Filled circle = F-Ratio. ^**a**^To improve the readability of these complex diagrams, ten papers[4,46,54,66,67,70,75,82,83,101] that had an outcome that appeared in only that one paper were omitted from this figure. Several analyses from other papers that had idiosyncratic outcomes are also excluded. For a comprehensive display of all of the data for all of the child outcomes see Supplemental Information S4 File. ^**b**^This paper is one of a series of Meta-Analyses and Systematic Reviews assessing the relationship between child care quality and children’s outcomes; therefore, superscript letters below are in reference to various large databases that samples in these papers were drawn from. These letters have been kept consistent across the series for our readers. ^**c**^Samples within papers are described in more detail in S3 File. ^d^Acronyms for child outcomes are listed in S6 File. ^**C**^Bermuda Preschool Study (1980); ^**O**^National Child Care Staffing Study (NCCSS, 1988.

**Fig 4:**
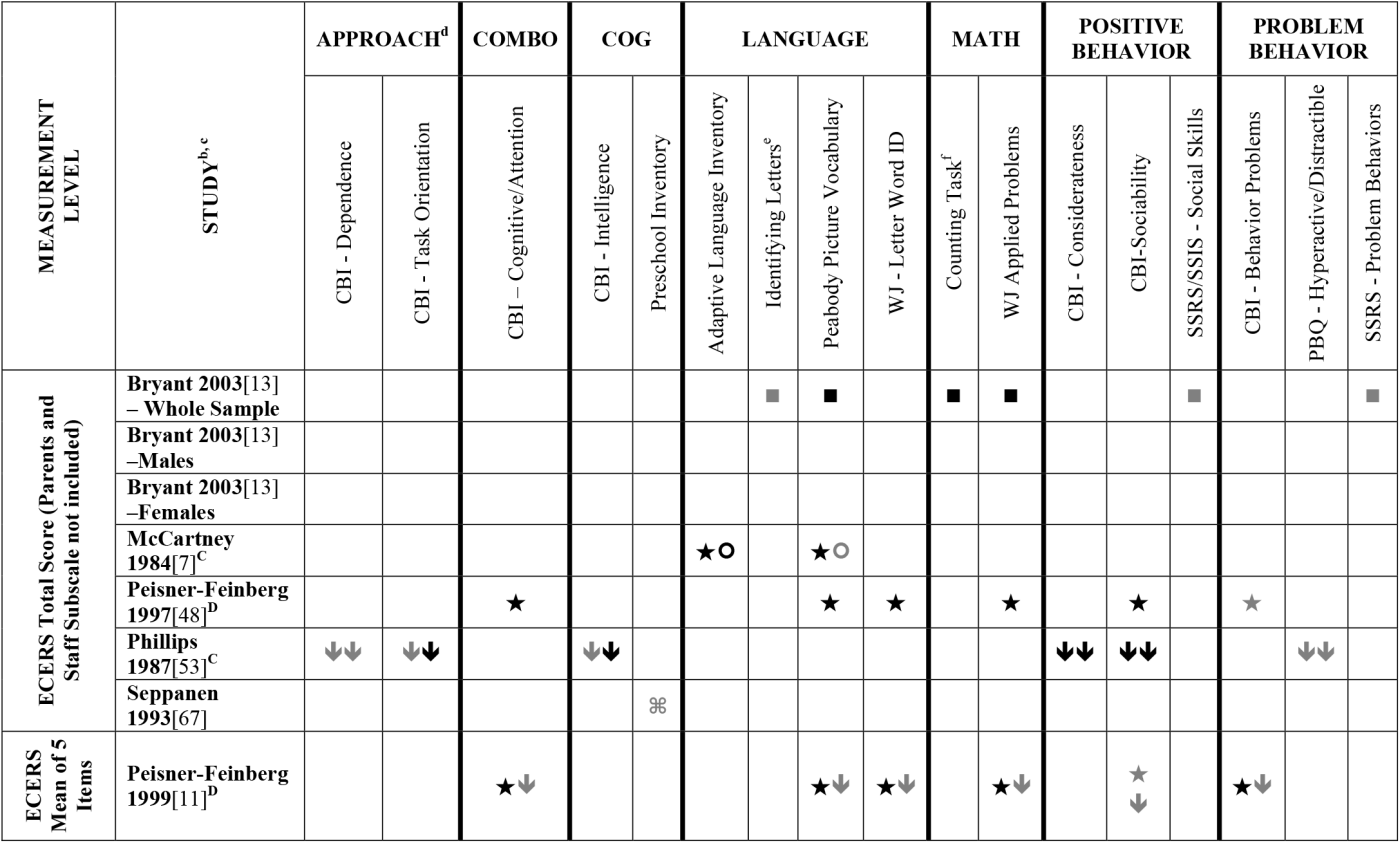
Systematic review of the associations between the ECERS total score (parents and staff subscale not included) and mean of 5 items and child outcomes. ^a^ Abbreviations: Symbols bolded are significant and positive, symbols bolded and italicized are significant and negative, and symbols in grey are non-significant. Star = Zero Order Pearson’s Correlation, Unfilled circle = Beta, Filled square = Unstandardized Coefficient, Black diamond minus white X = T-Test, Key clover = Partial Correlation, Downward arrow = Effect Size, Filled circle = F-Ratio. ^**a**^To improve the readability of these complex diagrams, ten papers[4,46,54,66,67,70,75,82,83,101] that had an outcome that appeared in only that one paper were omitted from this figure. Several analyses from other papers that had idiosyncratic outcomes are also excluded. For a comprehensive display of all of the data for all of the child outcomes see Supplemental Information S4 File.^**b**^This paper is one of a series of Meta-Analyses and Systematic Reviews assessing the relationship between child care quality and children’s outcomes; therefore, superscript letters below are in reference to various large databases that samples in these papers were drawn from. These letters have been kept consistent across the series for our readers. ^**c**^Samples within papers are described in more detail in S3 File. ^d^Acronyms for child outcomes are listed in S6 File. ^**e**^Identifying Letters (also referred to as Alphabet Recognition Test, Letter Identification, Letter Knowledge, Letter-Naming Test, Naming Letters). ^**f**^Counting Task (also referred to as Counting One-to-One, One-One Counting). ^**C**^Bermuda Preschool Study (1980); ^**D**^Cost, Quality and Outcomes Study (CQO, 1993–1994.

**Fig 5:**
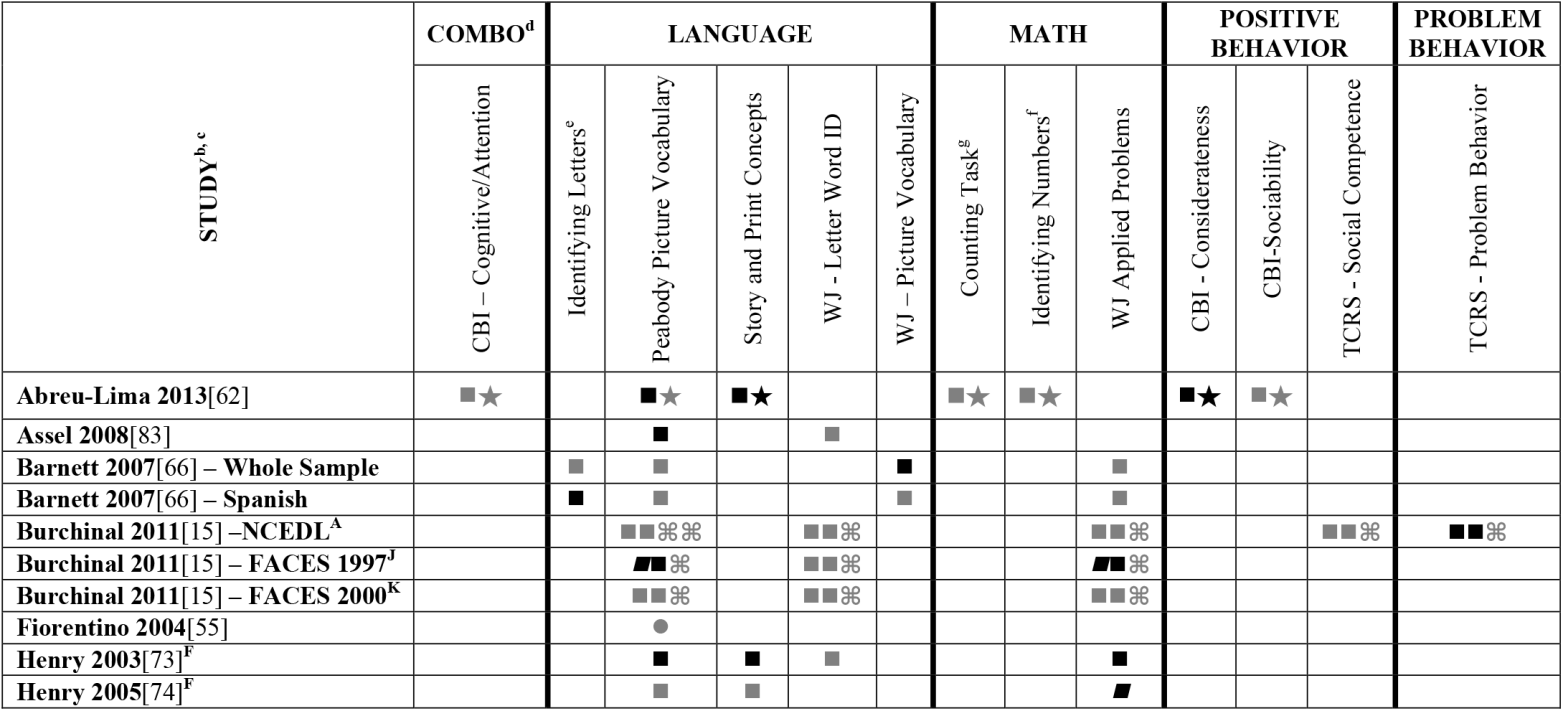
Systematic review of the associations between the ECERS-R total score, authors A-Hen and child outcomes. ^**A**^ Abbreviations: Symbols bolded are significant and positive, symbols bolded and italicized are significant and negative, and symbols in grey are non-significant. Star = Zero Order Pearson’s Correlation, Unfilled circle = Beta, Filled square = Unstandardized Coefficient, Black diamond minus white X = T-Test, Key clover = Partial Correlation, Downward arrow = Effect Size, Filled circle = F-Ratio. ^**a**^To improve the readability of these complex diagrams, ten papers[4,46,54,66,67,70,75,82,83,101] that had an outcome that appeared in only that one paper were omitted from this figure. Several analyses from other papers that had idiosyncratic outcomes are also excluded. For a comprehensive display of all of the data for all of the child outcomes see Supplemental Information S4 File. ^**b**^This paper is one of a series of Meta-Analyses and Systematic Reviews assessing the relationship between child care quality and children’s outcomes; therefore, superscript letters below are in reference to various large databases that samples in these papers were drawn from. These letters have been kept consistent across the series for our readers. ^**c**^Samples within papers are described in more detail in S3 File. ^d^Acronyms for child outcomes are listed in S6 File. ^**e**^Identifying Letters (also referred to as Alphabet Recognition Test, Letter Identification, Letter Knowledge, Letter-Naming Test, Naming Letters). ^**f**^Counting Task (also referred to as Counting One-to-One, One-One Counting). ^**g**^Identifying Numbers (also referred to as Naming Numbers, Number Identification). ^**A**^National Center for Early Development and Learning Dataset (NCEDL, 2002, 2004); ^**F**^Georgia Early Childhood Study (GECS, 2002); ^**J**^Head Start Family and Children Experiences Survey (FACES, 1997) Cohort; ^**K**^Head Start Family and Children Experiences Survey (FACES, 2000) Cohort.

**Fig 6:**
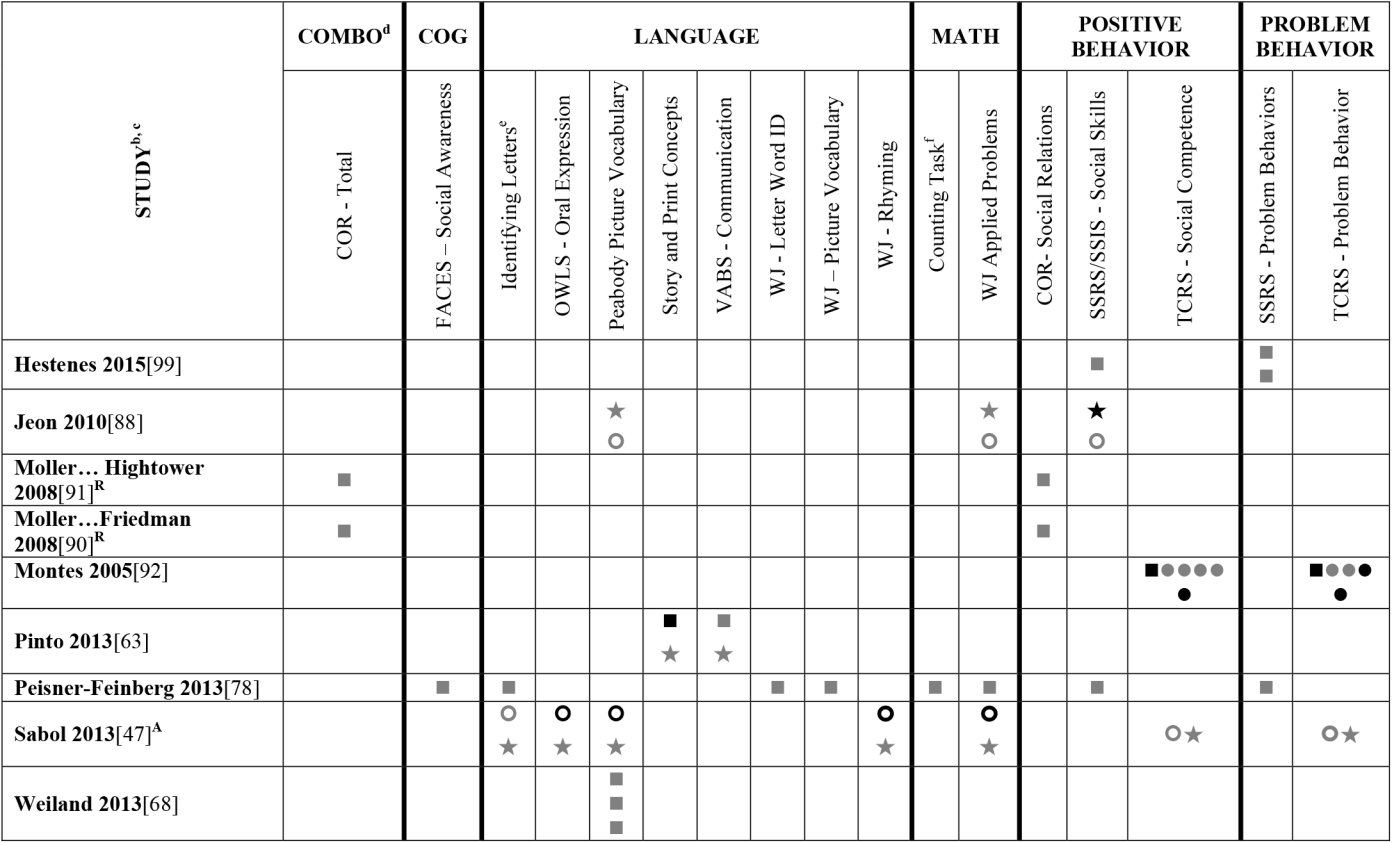
Systematic review of the associations between the ECERS-R total score, authors Hes-Z and child outcomes. ^a^Abbreviations: Symbols bolded are significant and positive, symbols bolded and italicized are significant and negative, and symbols in grey are non-significant. Star = Zero Order Pearson’s Correlation, Unfilled circle = Beta, Filled square = Unstandardized Coefficient, Black diamond minus white X = T-Test, Key clover = Partial Correlation, Downward arrow = Effect Size, Filled circle = F-Ratio. ^**a**^To improve the readability of these complex diagrams, ten papers[4,46,54,66,67,70,75,82,83,101] that had an outcome that appeared in only that one paper were omitted from this figure. Several analyses from other papers that had idiosyncratic outcomes are also excluded. For a comprehensive display of all of the data for all of the child outcomes see Supplemental Information S4 File.^**b**^This paper is one of a series of Meta-Analyses and Systematic Reviews assessing the relationship between child care quality and children’s outcomes; therefore, superscript letters below are in reference to various large databases that samples in these papers were drawn from. These letters have been kept consistent across the series for our readers. ^**c**^Samples within papers are described in more detail in S3 File. ^d^Acronyms for child outcomes are listed in S6 File. ^**e**^Identifying Letters (also referred to as Alphabet Recognition Test, Letter Identification, Letter Knowledge, Letter-Naming Test, Naming Letters). ^**f**^Counting Task (also referred to as Counting One-to-One, One-One Counting). ^**A**^National Center for Early Development and Learning Dataset (NCEDL, 2002, 2004); ^**R**^Northeastern United States sample (Moller and colleagues, 2008; Year NR).

**Fig 7:**
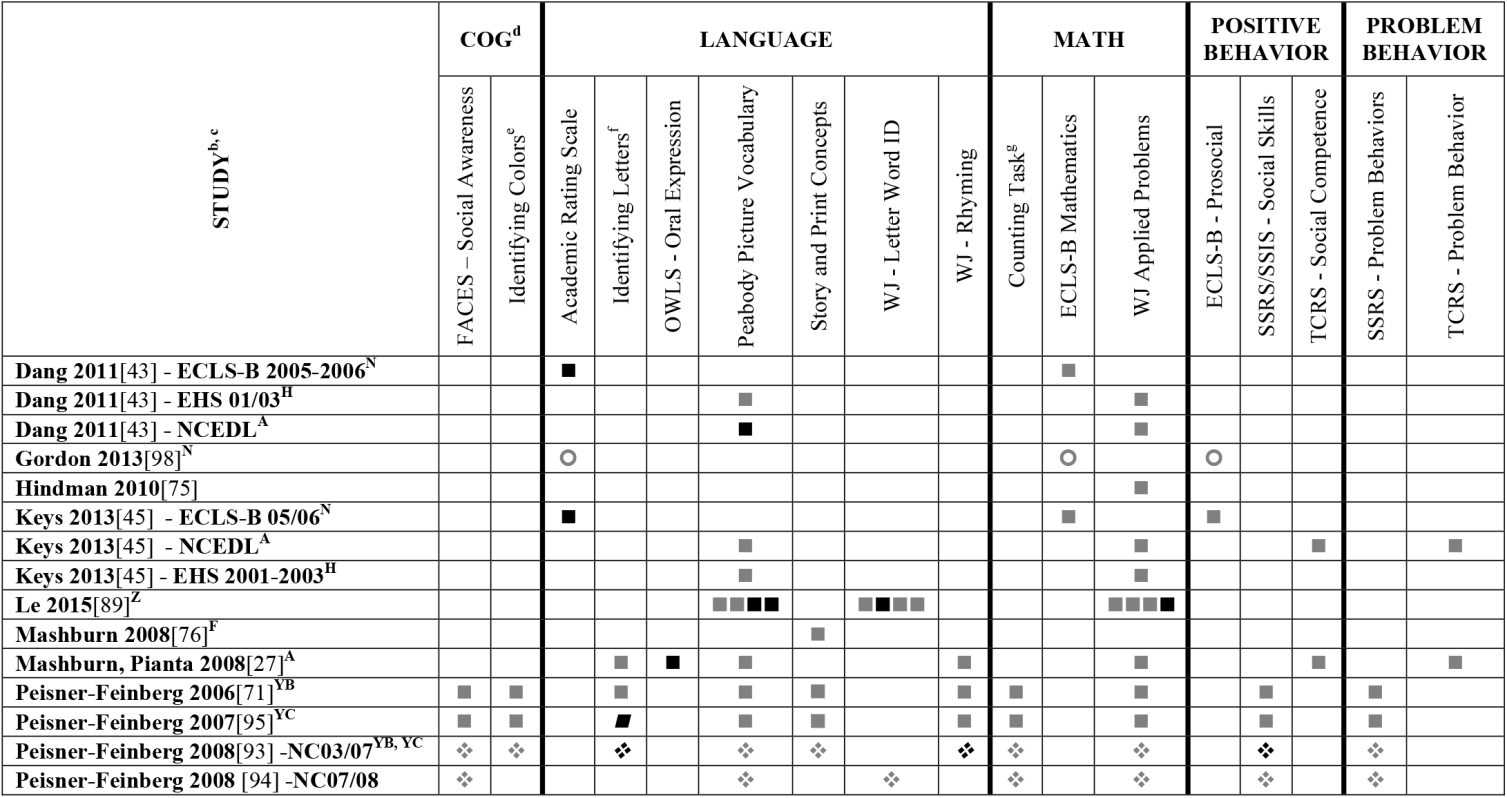
Systematic review of the associations between the ECERS-R total score, authors Hes-Z and child outcomes. ^a^Abbreviations: Symbols bolded are significant and positive, symbols bolded and italicized are significant and negative, and symbols in grey are non-significant. Star = Zero Order Pearson’s Correlation, Unfilled circle = Beta, Filled square = Unstandardized Coefficient, Black diamond minus white X = T-Test, Key clover = Partial Correlation, Downward arrow = Effect Size, Filled circle = F-Ratio. ^**a**^To improve the readability of these complex diagrams, ten papers[4,46,54,66,67,70,75,82,83,101] that had an outcome that appeared in only that one paper were omitted from this figure. Several analyses from other papers that had idiosyncratic outcomes are also excluded. For a comprehensive display of all of the data for all of the child outcomes see Supplemental Information S4 File. ^**b**^This paper is one of a series of Meta-Analyses and Systematic Reviews assessing the relationship between child care quality and children’s outcomes; therefore, superscript letters below are in reference to various large databases that samples in these papers were drawn from. These letters have been kept consistent across the series for our readers. ^**c**^Samples within papers are described in more detail in S3 File. ^d^Acronyms for child outcomes are listed in S6 File. ^**e**^Identifying Colors (also referred to as Color Knowledge, Color Naming, Color Naming Task). ^**f**^Identifying Letters (also referred to as Alphabet Recognition Test, Letter Identification, Letter Knowledge, Letter-Naming Test, Naming Letters). ^**g**^Counting Task (also referred to as Counting One-to-One, One-One Counting). ^**A**^National Center for Early Development and Learning Dataset (NCEDL, 2002, 2004); ^**F**^Georgia Early Childhood Study (GECS, 2002); ^**H**^Early Head Start (EHS, 2001–2003 Cohort); ^**N**^Early Childhood Longitudinal Study (ECLS-B, 2001–2006, Birth Cohort); ^Z^Colorado QRIS; ^**YB**^More at Four North Carolina Study (2003–2004) Cohort; ^**YC**^More at Four North Carolina Study (2005–2006) Cohort.

**Fig 8:**
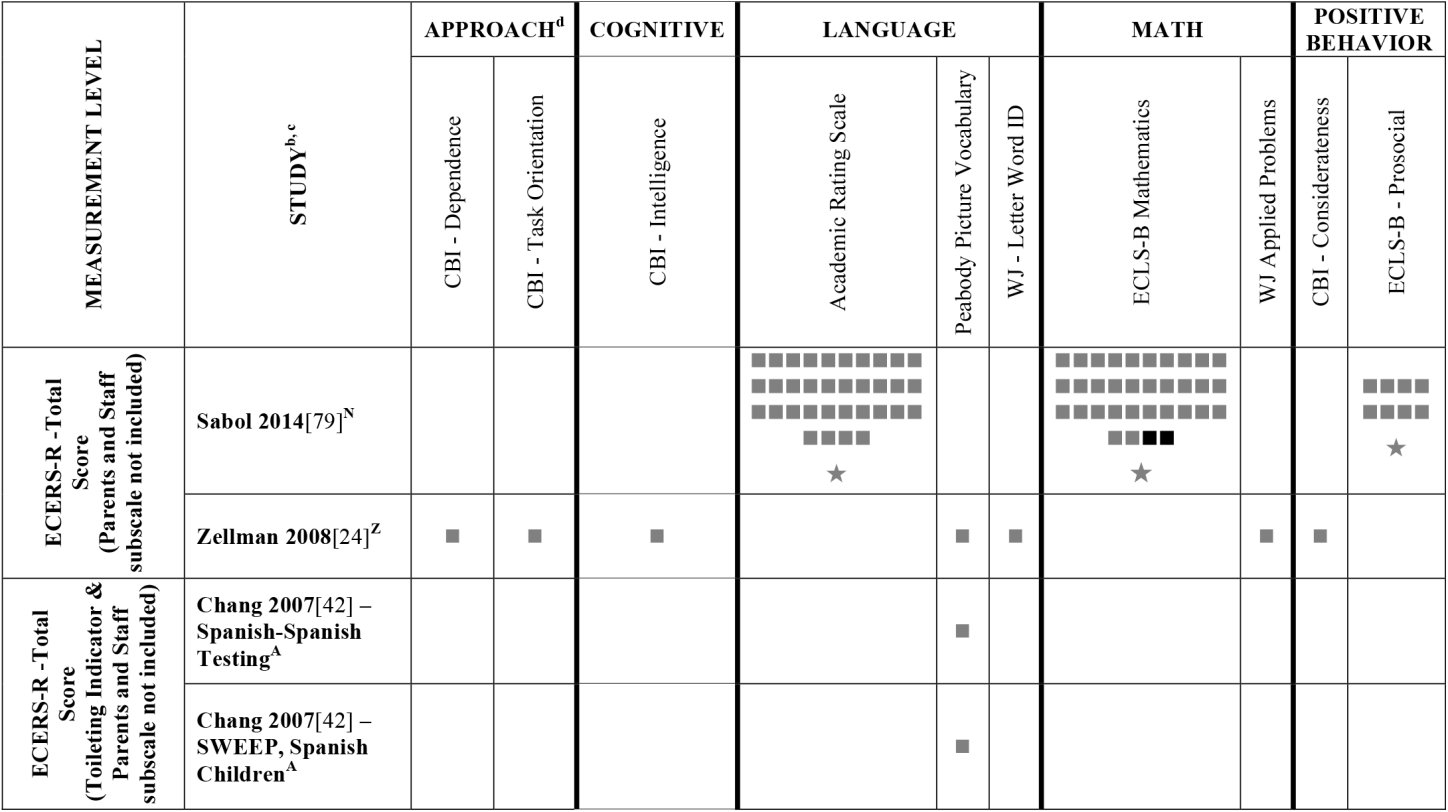
Systematic review of the associations between the ECERS-R total score, authors Hes-Z and child outcomes. ^a^Abbreviations: Symbols bolded are significant and positive, symbols bolded and italicized are significant and negative, and symbols in grey are non-significant. Star = Zero Order Pearson’s Correlation, Unfilled circle = Beta, Filled square = Unstandardized Coefficient, Black diamond minus white X = T-Test, Key clover = Partial Correlation, Downward arrow = Effect Size, Filled circle = F-Ratio. ^**a**^To improve the readability of these complex diagrams, ten papers[4,46,54,66,67,70,75,82,83,101] that had an outcome that appeared in only that one paper were omitted from this figure. Several analyses from other papers that had idiosyncratic outcomes are also excluded. For a comprehensive display of all of the data for all of the child outcomes see Supplemental Information S4 File. ^**b**^This paper is one of a series of Meta-Analyses and Systematic Reviews assessing the relationship between child care quality and children’s outcomes; therefore, superscript letters below are in reference to various large databases that samples in these papers were drawn from. These letters have been kept consistent across the series for our readers. ^**c**^Samples within papers are described in more detail in S3 File. ^d^Acronyms for child outcomes are listed in S6 File. ^**A**^National Center for Early Development and Learning Dataset (NCEDL, 2002, 2004); ^**N**^Early Childhood Longitudinal Study (ECLS-B, 2001–2006, Birth Cohort); ^**Z**^Colorado QRIS.

**Fig 9:**
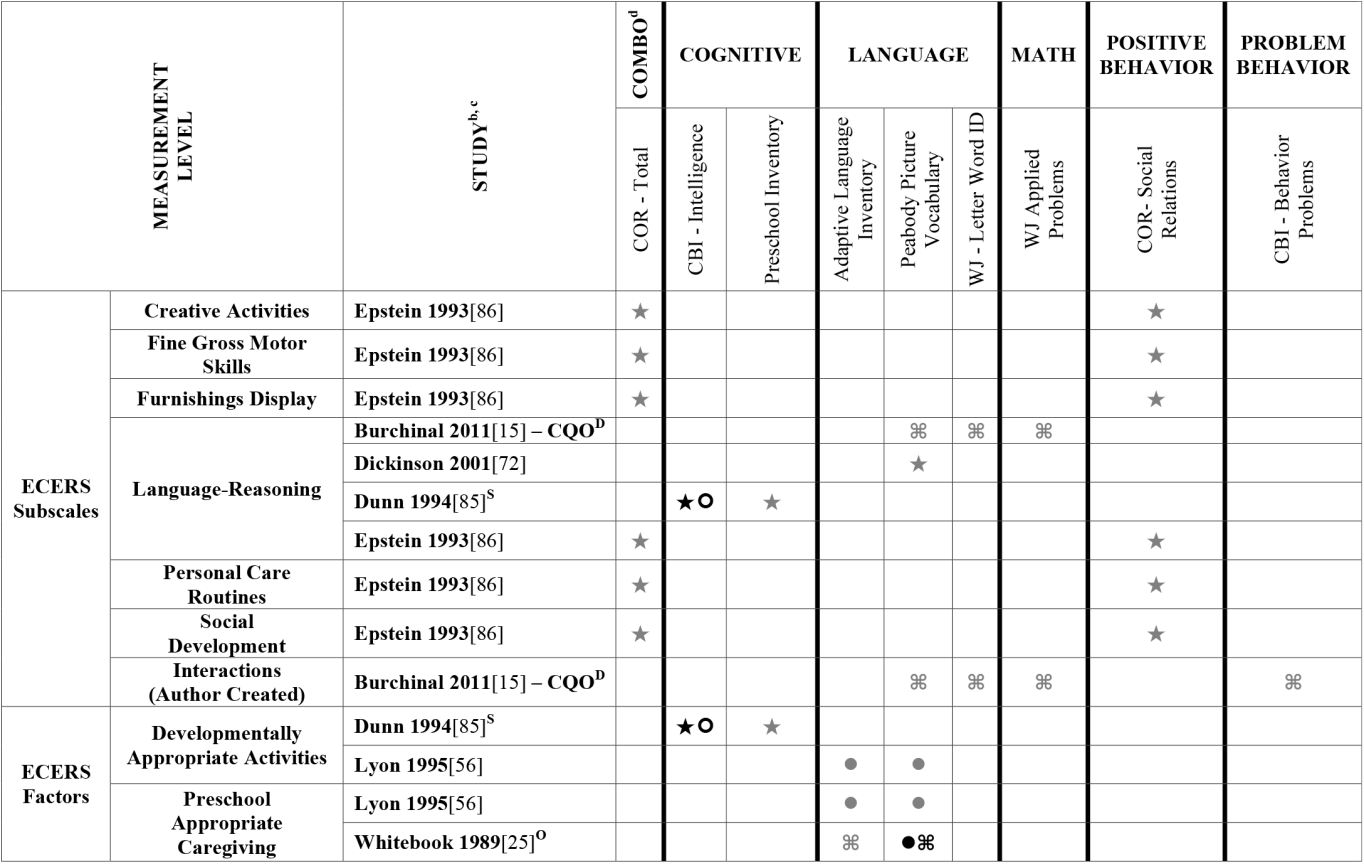
Systematic review of the associations between the ECERS-R total score, authors Hes-Z and child outcomes. ^a^ Abbreviations: Symbols bolded are significant and positive, symbols bolded and italicized are significant and negative, and symbols in grey are non-significant. Star = Zero Order Pearson’s Correlation, Unfilled circle = Beta, Filled square = Unstandardized Coefficient, Black diamond minus white X = T-Test, Key clover = Partial Correlation, Downward arrow = Effect Size, Filled circle = F-Ratio. ^**a**^To improve the readability of these complex diagrams, ten papers[4,46,54,66,67,70,75,82,83,101] that had an outcome that appeared in only that one paper were omitted from this figure. Several analyses from other papers that had idiosyncratic outcomes are also excluded. For a comprehensive display of all of the data for all of the child outcomes see Supplemental Information S4 File. ^**b**^This paper is one of a series of Meta-Analyses and Systematic Reviews assessing the relationship between child care quality and children’s outcomes; therefore, superscript letters below are in reference to various large databases that samples in these papers were drawn from. These letters have been kept consistent across the series for our readers. ^**c**^Samples within papers are described in more detail in S3 File. ^d^Acronyms for child outcomes are listed in S6 File. ^**D**^Cost, Quality and Outcomes Study (CQO, 1993–1994); ^**O**^National Child Care Staffing Study (NCCSS, 1988); ^**S**^8-county region of North-Central Indiana (Year NR).

**Fig 10:**
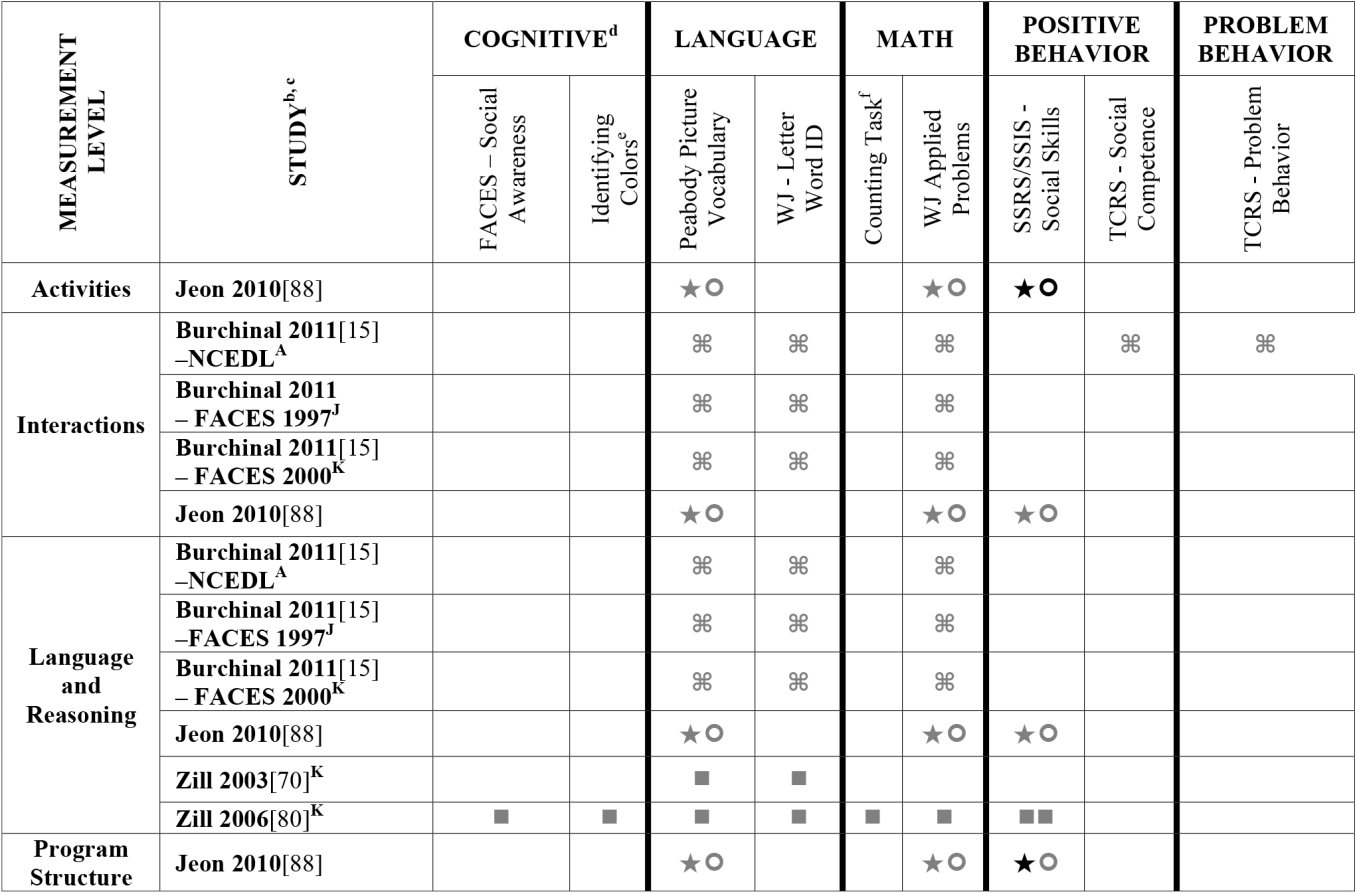
Systematic review of the associations between the ECERS-R total score, authors Hes-Z and child outcomes. ^a^Abbreviations: Symbols bolded are significant and positive, symbols bolded and italicized are significant and negative, and symbols in grey are non-significant. Star = Zero Order Pearson’s Correlation, Unfilled circle = Beta, Filled square = Unstandardized Coefficient, Black diamond minus white X = T-Test, Key clover = Partial Correlation, Downward arrow = Effect Size, Filled circle = F-Ratio. ^**a**^To improve the readability of these complex diagrams, ten papers[4,46,54,66,67,70,75,82,83,101] that had an outcome that appeared in only that one paper were omitted from this figure. Several analyses from other papers that had idiosyncratic outcomes are also excluded. For a comprehensive display of all of the data for all of the child outcomes see Supplemental Information S4 File. ^**b**^This paper is one of a series of Meta-Analyses and Systematic Reviews assessing the relationship between child care quality and children’s outcomes; therefore, superscript letters below are in reference to various large databases that samples in these papers were drawn from. These letters have been kept consistent across the series for our readers. ^**c**^Samples within papers are described in more detail in S3 File. ^d^Acronyms for child outcomes are listed in S6 File. ^**e**^Identifying Colors (also referred to as Color Knowledge, Color Naming, Color Naming Task). ^**f**^Counting Task (also referred to as Counting One-to-One, One-One Counting). ^**A**^National Center for Early Development and Learning Dataset (NCEDL, 2002, 2004); ^**J**^Head Start Family and Children Experiences Survey (FACES, 1997) Cohort; ^**K**^Head Start Family and Children Experiences Survey (FACES, 2000) Cohort.

**Fig 11:**
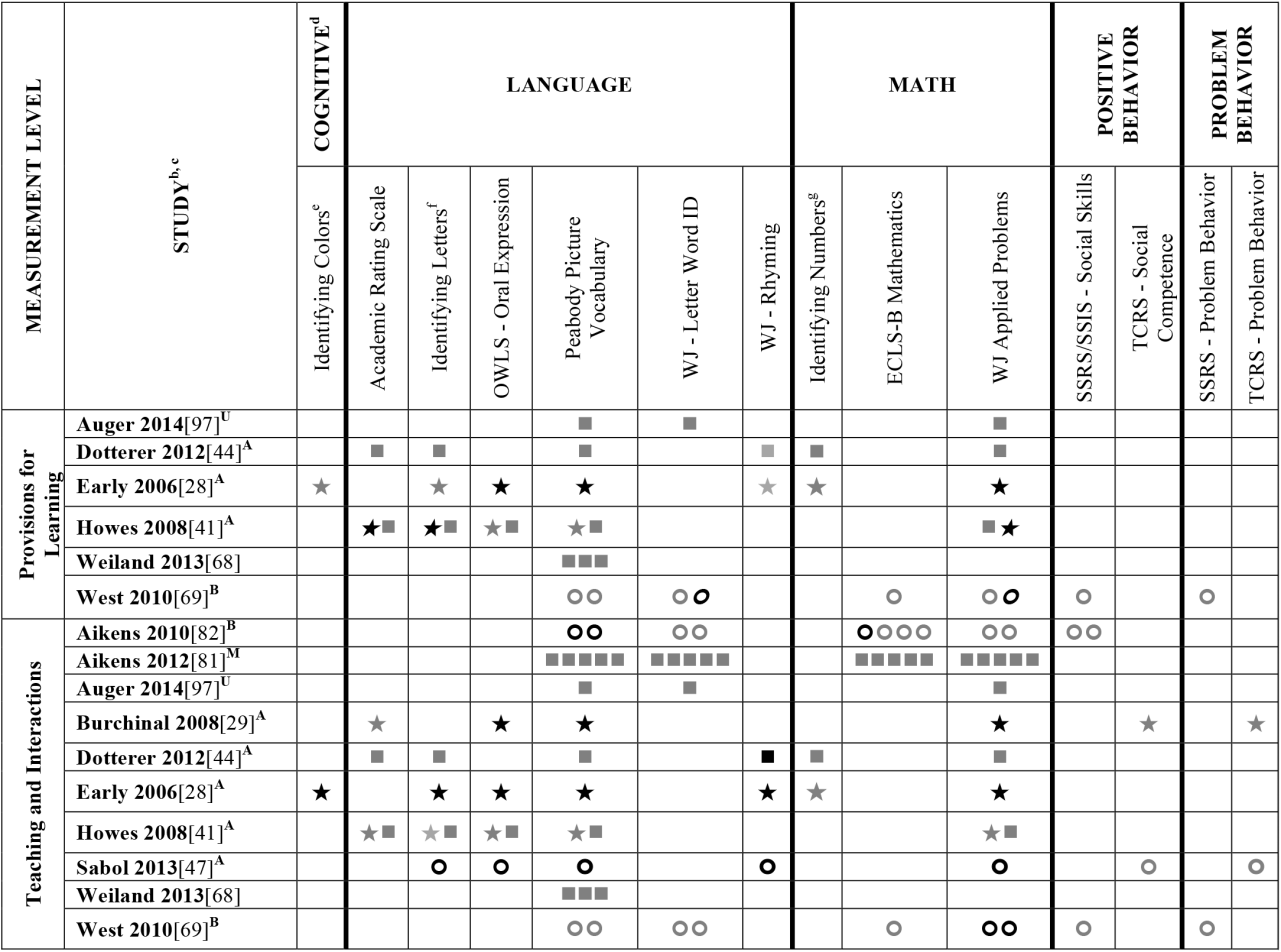
Systematic review of the associations between the ECERS-R factors and child outcomes. ^a^ Abbreviations: Symbols bolded are significant and positive, symbols bolded and italicized are significant and negative, and symbols in grey are non-significant. Star = Zero Order Pearson’s Correlation, Unfilled circle = Beta, Filled square = Unstandardized Coefficient, Black diamond minus white X = T-Test, Key clover = Partial Correlation, Downward arrow = Effect Size, Filled circle = F-Ratio. ^**a**^To improve the readability of these complex diagrams, ten papers[4,46,54,66,67,70,75,82,83,101] that had an outcome that appeared in only that one paper were omitted from this figure. Several analyses from other papers that had idiosyncratic outcomes are also excluded. For a comprehensive display of all of the data for all of the child outcomes see Supplemental Information S4 File. ^**b**^This paper is one of a series of Meta-Analyses and Systematic Reviews assessing the relationship between child care quality and children’s outcomes; therefore, superscript letters below are in reference to various large databases that samples in these papers were drawn from. These letters have been kept consistent across the series for our readers. ^**c**^Samples within papers are described in more detail in S3 File. ^d^Acronyms for child outcomes are listed in S6 File. ^**e**^Identifying Colors (also referred to as Color Knowledge, Color Naming, Color Naming Task). ^**f**^Identifying Letters (also referred to as Alphabet Recognition Test, Letter Identification, Letter Knowledge, Letter-Naming Test, Naming Letters). ^**g**^Identifying Numbers (also referred to as Naming Numbers, Number Identification). National Center for Early Development and Learning Dataset (NCEDL, 2002, 2004); ^**B**^Head Start Family and Children Experiences Survey (FACES, 2006) Cohort; ^**M**^Head Start Family and Children Experiences Survey (FACES, 2009) Cohort ^**U**^Preschool Curriculum Evaluation Research (PCER, 1999–2003).

Data extracted from the 73 eligible studies are presented in Tables A-J in S5 File. A snapshot of the results is also provided in Figs 2–11, which displays the results obtained for only those child outcomes that were used in three or more samples. For each table, each row represents a single sample within a paper and each cell represents the various analyses that were conducted with a specific sample.

Studies looking at Approach [15,29,52,68,69,75,100], Cognitive [4,5,27,29,30,34,48,50,53,66,68–70,75,77,80,84,85,92,94,98–100,102–106], and Positive Behavior outcomes [11,13,15,27,29,30,33,35,44,45,50–53,60–62,64,68–70,74,75,77,80,81,85–88,92–94,96,98,100,102–106,108] showed very few significant associations with ECERS/ECERS-R total scores. Several studies included outcomes that combined various developmental screeners (labeled ‘Combination’ in S4 File). These showed virtually no significant associations with the ECERS/ECERS-R total score. A somewhat higher number of significant associations were reported for analyses in which Mathematics and Problem Behavior were the outcomes. However, the direction of results was inconsistent with some studies reporting positive associations and others reporting negative associations [15,52,90]. Further, a large number of significant positive associations for Mathematics outcomes came from the NCEDL dataset and as such should be interpreted with caution as they draw from the same sample of children. Finally, of the 168 outcomes, 52 were related to children’s language development with the PPVT, WJ-LWI and author created letter identification tasks reported most frequently across studies. Several positively significant associations were identified between ECERS/ECERS-R total scores and Language outcomes, however most studies reporting this association used a single dataset (CQO).

Qualitative review revealed more significant associations between ECERS total scores than ECERS-R total scores and children’s Language outcomes, particularly for the PPVT. Studies using the ECERS-R were often conducted later and were more likely to use analyses that controlled for child and family characteristics. Thus, the lower number of significant associations identified between ECERS-R (as opposed to the ECERS) and child outcomes may reflect better quality in the studies that tested association with the ECERS-R.

#### ECERS/ECERS-R subscales/factors

Virtually no associations were identified between ECERS/ECERS-R subscales or factors and Approach outcomes. A few positive associations were identified between ECERS/ECERS-R subscales/factors and Cognitive outcomes, particularly the Language-Reasoning subscale and the Developmentally Appropriate Activities factor. However, these were largely driven by a single study [99]. Similarly, the very few positive associations that were identified between ECERS/ECERS-R subscales/factors and Combo outcomes were largely derived from a single study [100]. Virtually no associations were identified for any subscale or factor of the ECERS/ECERS-R and social-emotional outcomes. However, more associations were noted for Positive Behaviors when the ‘Parents and Staff’ subscale was removed from the ECERS. A few significant associations were identified between Provisions for Learning (PL) and Teaching and Interactions (TI) and Mathematics outcomes, though most came from the NCEDL dataset. More significant positive associations were identified with Mathematics outcomes when the ‘Parents and Staff’ subscale was removed from the ECERS. Finally, few significant associations were identified between ECERS/ECERS-R subscales/factors and Language outcomes. Of note, several of these associations were identified for the PL and TI factors, factors that are conceptually linked to language development. However, negative associations were also identified for the PL factor, and the majority of the associations with the TI factor were from the NCEDL dataset. Overall, a higher number of significant positive associations are evident for the ECERS/ECERS-R total score than were identified for individual factors, subscales or shorter versions of the measure.

### Meta-analyses

The number of studies included in the meta-analyses is small compared to those included in the systematic review. This is because of the methodological heterogeneity in studies included in the review. For the meta-analyses, we only pooled data when we were confident that studies were sufficiently homogeneous.

**Fig 12:**
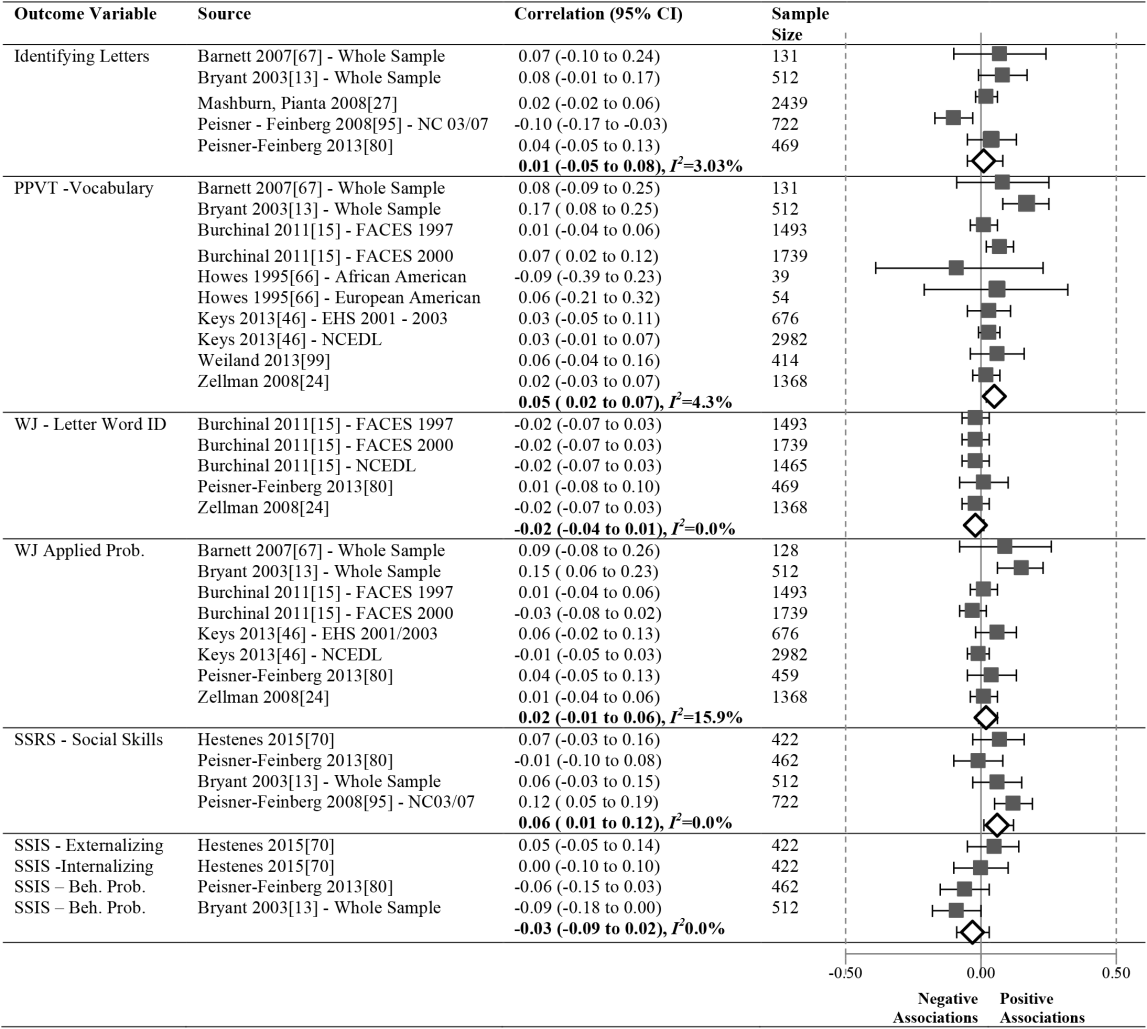
Meta-analyses of the association between ECERS/ECERS-R total score and child outcomes.

**Fig 13:**
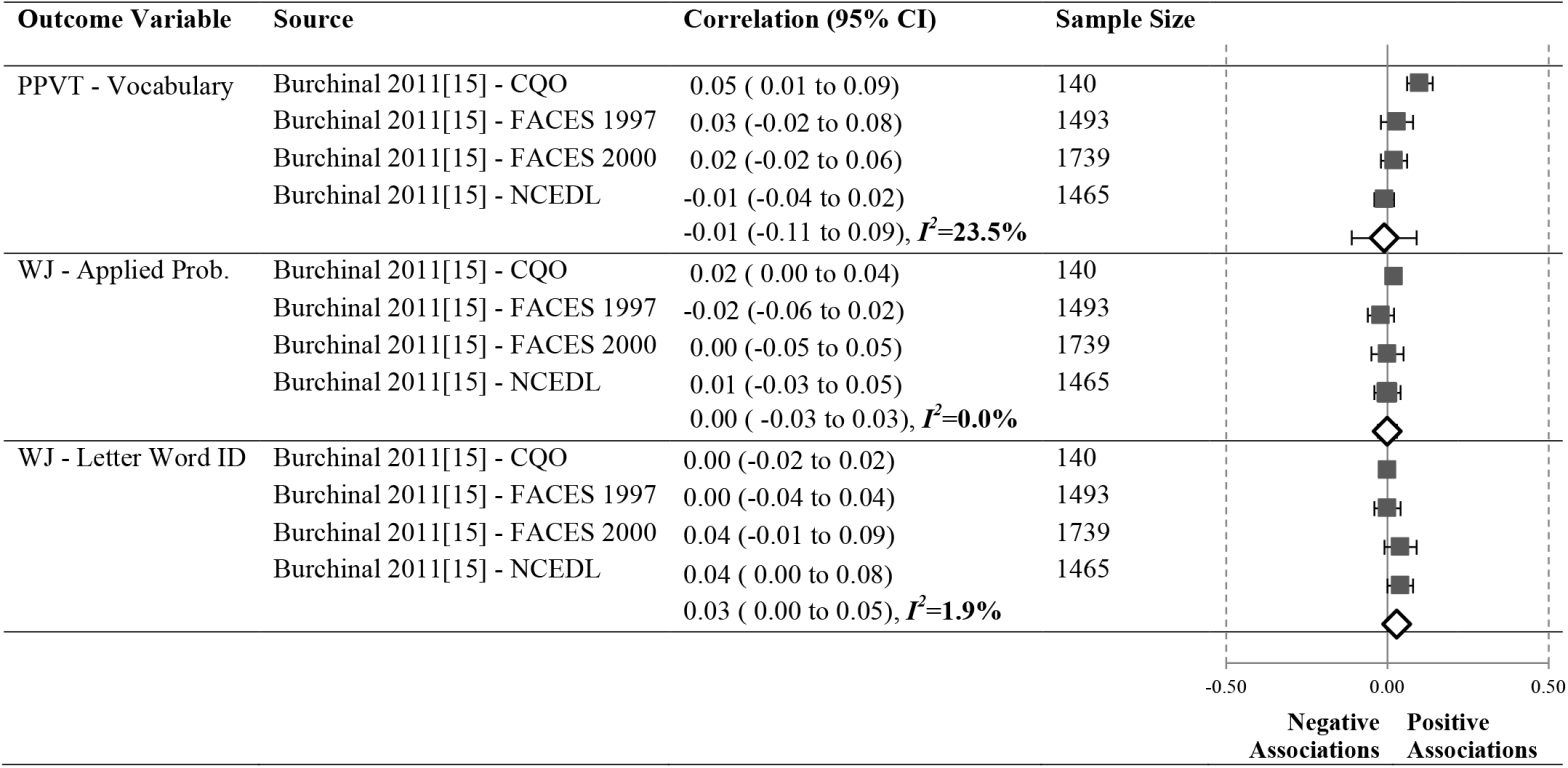
Meta-analyses of the association between ECERS/ECERS-R interactions subscale and child outcomes.

**Fig 14:**
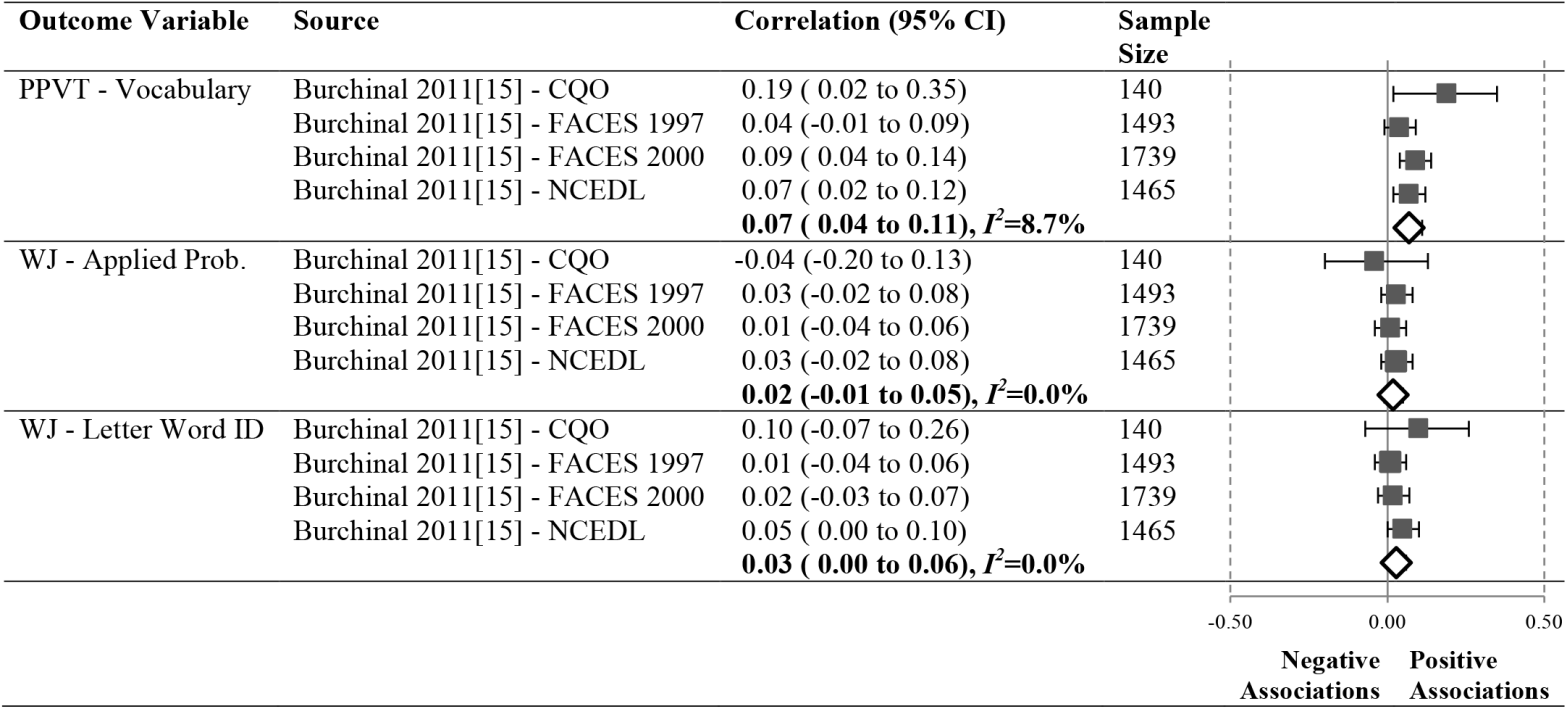
Meta-analyses of the association between ECERS/ECERS-R language reasoning subscale and child outcomes.

**Fig 15:**
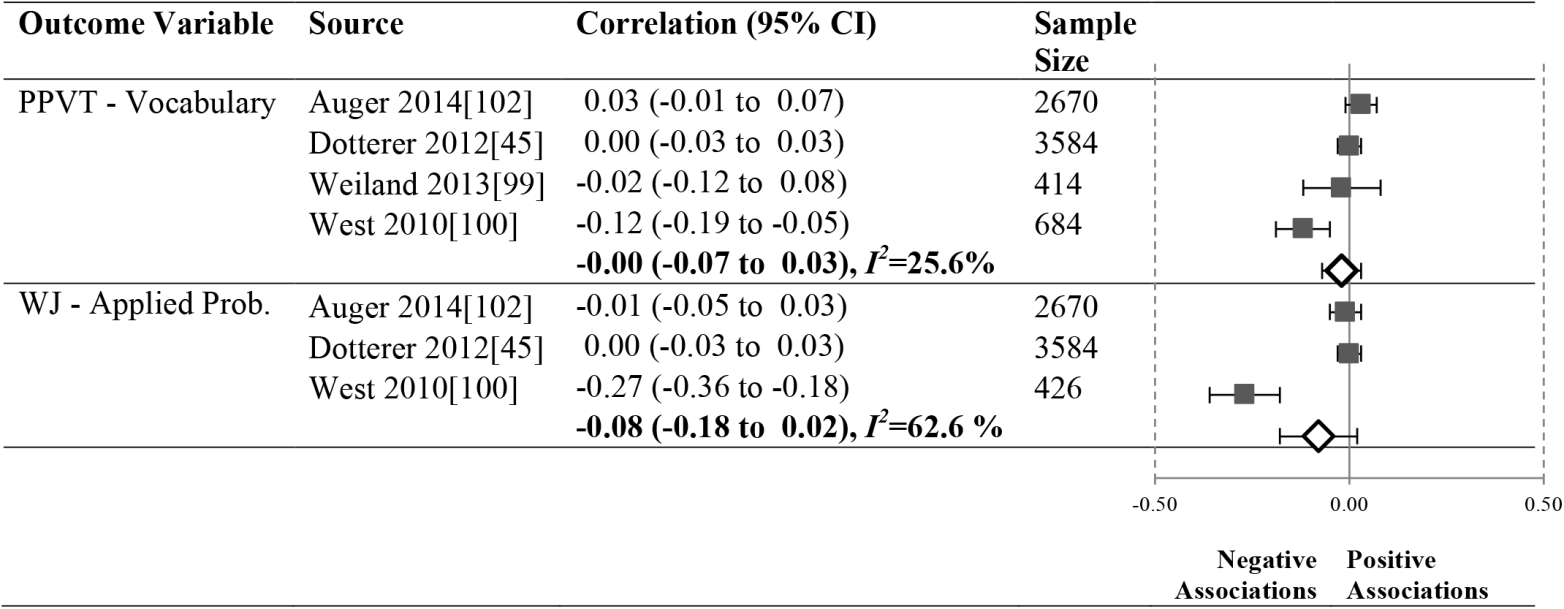
Meta-analyses of the association between ECERS-R provisions for learning factor and child outcomes.

**Fig 16:**
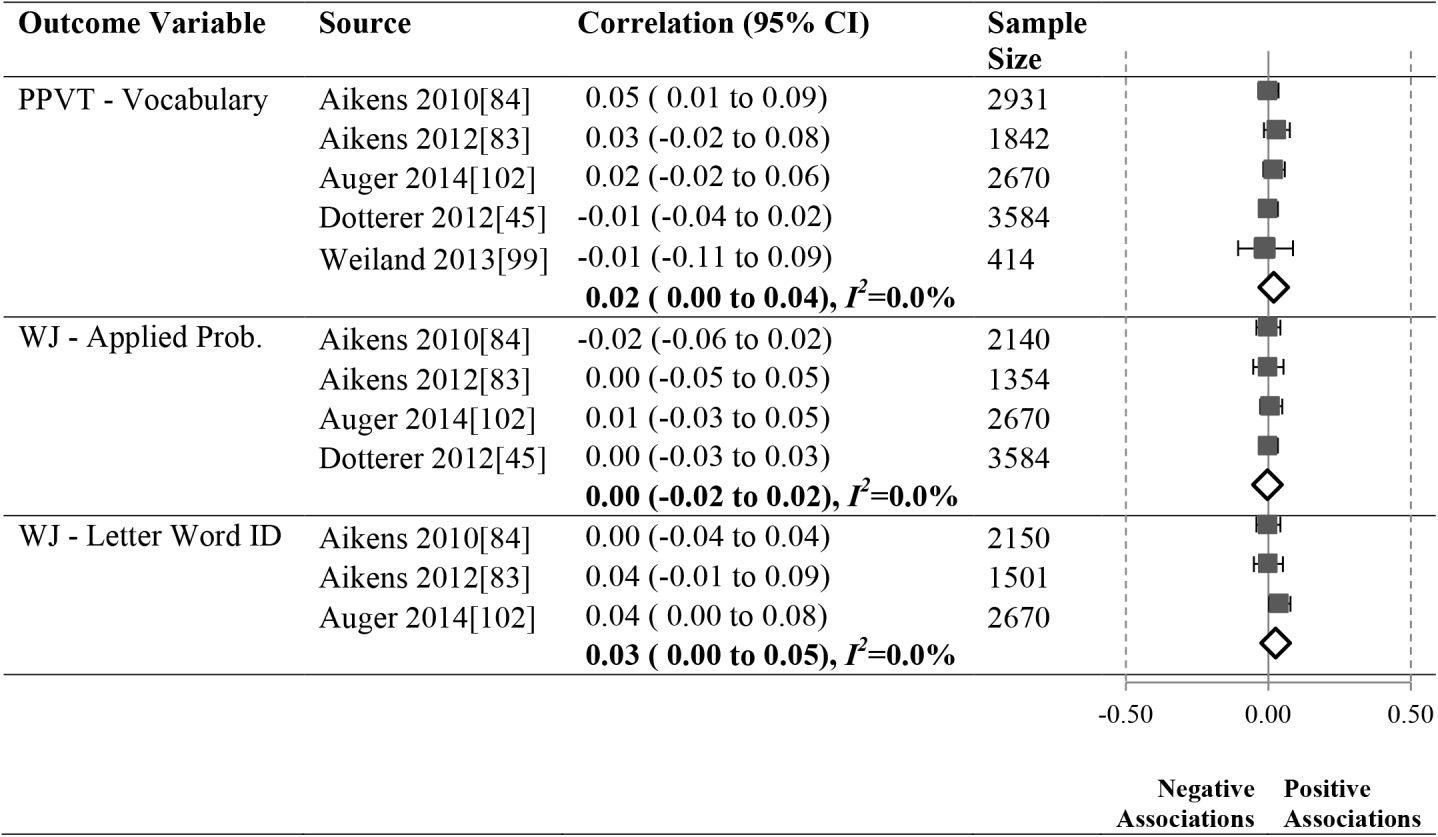
Meta-analyses of the association between ECERS-R teaching and interactions factor and child outcomes.

As shown in Figs 12–16, 16 studies (21 samples) met our criteria, reporting 17 unique relationships of a particular operationalization of the ECERS/ECERS-R and an outcome that could be meta-analyzed. The number of studies in our meta-analyses ranged from 3 to 10. To ensure simplicity and for ease of presentation, outcomes were grouped under the Language, Mathematics and Social-Emotional headings.

I^2^ values were low across the significant meta-analyses we conducted, ranging from 0.00–8.70 with an average I^2^ value of 4.67. I^2^ values were somewhat higher in the non-significant meta-analyses ranging from 0.00–62.6 with an average I^2^ value of 9.74.

### Meta-analysis results by child outcome

We conducted a total of 6 meta-analyses between ECERS/ECERS-R total scores and children’s Language, Mathematics and Social-Emotional outcomes (Fig 12).

#### Language

Meta-analyses were conducted for three unique Language outcomes (Fig 12).

A significant but small positive correlation was revealed between the ECERS/ECERS-R total score and the PPVT (N = 9408, pooled correlation coefficient 0.05; 95%CI: 0.02 to 0.07). Pooled results for author developed measures of Identifying Letters (N = 4273) and WJ-LWI (N = 6534) were not significant.

#### Mathematics

A meta-analysis was conducted for a single Math outcome (Fig 12). No significant relationship was identified for WJ-AP (N = 9357).

#### Social-emotional

One meta-analysis assessed the correlation between ECERS/ECERS-R and Positive Behavior (Fig 12). The SSRS-Social Skills (N = 2118, pooled correlation coefficient 0.06; 95%CI:0.01 to 0.12) revealed a weak positive effect. Another meta-analysis found a non-significant relationship between the ECERS/ECERS-R total score and SSIS-Problem Behavior (N = 1818).

### Meta-analysis results by ECERS/ECERS-R subscales and factors

We were able to investigate the effect of two ECERS/ECERS-R subscales and two factors and children’s Language and Mathematics outcomes (Figs 13–16):

#### Interactions

Associations between the ECERS/ECERS-R Interactions subscale and the PPVT (N = 4837), WJ-AP (N = 4837) and WJ-LWI (N = 4837) were not significant (See Fig 13).

#### Language-reasoning

A small, significant positive correlation was revealed between the Language-Reasoning subscale (See Fig 14) and the PPVT (N = 4837, pooled correlation coefficient 0.07; 95%CI: 0.04 to 0.11). The correlations between Language-Reasoning and the WJ-AP subscale (N = 4928) and the WJ-LWI subscale (N = 4837) were not significant.

#### Provisions for learning

Associations between the ECERS/ECERS-R Provisions for Learning factor and the PPVT (N = 7352) and WJ-AP (N = 6680) were non-significant (See Fig 15).

#### Teaching and interactions

No significant associations were identified between the Teaching and Interactions factor (See Fig 16) and Language outcomes, PPVT (N = 11, 441) and WJ-LWI (N = 6231), or Mathematics outcomes, WJ-AP (N = 9748).

## Discussion

The last 40 years have seen a dramatic increase in the number of children enrolled in regulated child care [109]. Expenditures on ECEC programs have also grown substantially [110]. Research about associations between quality of ECEC environments and child functioning has been conducted in an effort to improve public policy and practice in this area [111,112].

The ECERS/ECERS-R is the most commonly used measure of global quality for preschool-aged classrooms [102]. Developed in the early 1980s, it has been used internationally by researchers and practitioners, accumulating over 30 years of data on its reliability and validity. Yet, a comprehensive understanding of its relationship to child outcomes through a systematic review has not been conducted. Despite heterogeneity in this area of research we were able to integrate data from a large number of studies. Average ECERS/ECERS-R total scores ranged from 2.9 (0.45) [66] considered to be poor quality, to 6.52 (0.64) [104], considered to be of good quality. However, the vast majority of the programs in eligible studies were just above minimal quality (total score of 4), with only nine reporting total scores that were good (i.e., 5 or greater)[15,50,52,60,83,85,102–104], indicating that most centres provide mediocre care. Overall, results of the meta-analyses show a few (3 out of 17) significant, albeit weakly positive relationships were identified for the ECERS/ECERS-R total score and Language and Positive Behavior outcomes.

Empirical studies of the ECERS/ECERS-R generally find that it is made up of either one or two factors [14,30,31]. We were able to meta-analyze two subscales and two factors of the ECERS/ECERS-R. While the Interactions subscale did not reveal any associations with child outcomes, the Language-Reasoning subscale was associated with a Language outcome, but not a Mathematics outcome. When looking at factors, neither the Teaching and Interactions factor nor the Provisions for Learning factor showed any significant associations with child outcomes. These results suggest that while there is some modest evidence to support the use of a particular subscale, use of the total score may be preferable. It is noteworthy that the subscale that did reveal an association with a child outcome was one that assessed the quality of language used in the classroom, rather than materials available to children. Further, this association was identified for a measure of receptive language (PPVT). This should come as no surprise as this subscale focuses on encouraging children to communicate and to use language to develop their reasoning skills. Recent research has highlighted the importance of the quality of staff-child interactions in developing children’s language [33,113,114]. Perhaps the fact that the ECERS/ECERS-R is made up of both structural and process items explains the small magnitude of associations.

### Limitations

Integrating findings in this review was difficult because of methodological limitations of many of the primary studies identified in our searches. One issue is that all of the studies are observational/correlational and thus results from these studies do not imply causality. We also identified large methodological heterogeneity in measurement approaches, for both the ECERS/ECERS-R and child outcomes.

To avoid threats to validity of combining studies that are too heterogeneous we only combined studies that used identical operationalizations of the ECERS/ECERS-R within a single meta-analysis. The one exception to this is that we combined studies that used total scores for the ECERS and ECERS-R based on findings that the two versions are highly correlated [14,20].

The fact that researchers used a wide array of child outcomes was both a strength and a limitation of this review. On the one hand, results of this review are comprehensive in that they cover a very wide range of child outcomes. On the other, many outcomes were only reported in one or two studies, which meant that we could not meta-analyze across them. Fortunately, the child outcomes that were most prevalent in the literature (e.g., PPVT, WJ-AP) are standardized measures that have been well researched, allowing us to meta-analyze results from studies that used psychometrically strong child outcome measures. That being said, our goal was to look at child outcomes that go “beyond achievement texts”. While we were certainly able to do this across the systematic review and meta-analyses we conducted, the meta-analyses in particular were possible largely for measures that fall under the “school readiness” category. Researchers should include social/emotional outcomes so that associations between the ECERS-R and a broader range of outcomes can be meta-analyzed in the future. In addition, future research on the impact of ECEC quality on child outcomes should focus on established measures with good psychometric properties to assess specific aspects of child development that are conceptually linked to the specific aspect of ECEC quality in question.

There was also enormous variability in the covariates used in different studies. More recent studies tend to control for more child/family and program variables. This may explain why we saw more significant associations for ECERS than for the more recent ECERS-R total scores, particularly for Language outcomes. To minimize the impact of this issue, we only included statistics that accounted for covariates in our meta-analyses. Unfortunately, because of the limited number of studies that were deemed meta-analyzable, we did not have the sample size needed to test for specific moderators statistically. In the meta-analysis, we dealt with this by only including statistics from analyses in which covariates were used. In the systematic review, we dealt with this by exploring patterns in the results, such as characteristics of the samples of families and children, whether papers were peer reviewed or reports and whether the papers/samples were part of a large dataset.

Another problem with the ECEC literature in general, and the ECERS/ECERS-R literature in particular, is the lack of variation in program quality. In the 73 studies included in this paper, most ECERS/ECERS-R total scores fell in the mediocre range (score of 4). While limited variability may influence the significance level of associations, it is important to note that there was still non-trivial variability in the quality of programs represented in this meta-analysis and systematic review.

Reducing the ECERS/ECERS-R items by averaging across items to get a total score reduces the richness of how the measure captures quality. As a result, two classrooms can receive similar scores despite having met vastly different characteristics. Using a global measure, with a broad scope that is often not strongly linked to outcomes of interest (i.e., ability to identify letters and numbers) may help explain the limited associations identified in this study. Consistent with this, in the systematic review we found more significant associations when outcomes were more closely conceptually linked to a specific ECERS/ECERS-R factor.

Another methodological issue is that there is a mismatch in measurement units with the ECERS/ECERS-R (captured at the classroom level) and child outcomes (taken at the child level). These methodological limitations also reflect a lack of refinement in the conceptualization of quality. For example, capturing quality at the classroom level may mask important differences in the experiences of individual children within a classroom. Clearly more research that is methodologically and conceptually rigorous is needed in this area.

Finally, as we noted above, we were not able to rate the quality of the papers included in our meta-analyses, and despite our efforts to address the many methodological limitations of the studies we reviewed, our ability to understand the effect of the study design on estimates is limited. This is because (a) our sample size did not allow us to statistically test for moderator effects, (b) for ethical and logistical reasons, research in this area is correlational and thus does not allow for causative modeling, (c) many studies either did not account for, or only accounted for some of the confounders, and (d) there are biases associated with exposure and outcome ascertainment in many studies. Nonetheless, a systematic review and meta-analyses of existing studies, that identifies these deficiencies, allows us to learn from the existing literature and develop a way forward for future studies. Furthermore, it is worth noting that our meta-analysis inclusion criteria selected for studies that are relatively strong methodologically. For example, because we only included studies in which the outcome measure was identical, we ended up with more frequently used measures that also tend to be better researched and therefore more psychometrically sound (e.g., the PPVT). Similarly, by selecting statistics from analyses in which the covariates were used and by selecting the study with the largest sample size when multiple papers reported on the same dataset, we ended up including studies that were generally stronger.

### Implications for research

Despite the substantial limitations of research in this area, the current study found some evidence to support a positive relationship between the ECERS/ECERS-R and child outcomes. While the associations were small in magnitude, they were rarely negative, suggesting that the ECERS/ECERS-R captures aspects of the environment that are important to child development. However, for now, the small associations identified in this meta-analysis and systematic review suggests that investment in the measurement of other quality indicators (e.g., staff development) and the development of measures that accurately assess children’s experiences in ECEC classrooms is warranted. In addition, standardization of measurement and reporting of outcomes is needed so that in the future, results from more studies can be pooled.

## Supporting information

S1 File Search syntax ECERS. (PDF)

S2 File Formulas ECERS. (PDF)

S3 File Descriptives ECERS. (PDF)

S4 File Child outcomes ECERS. (PDF)

S5 File Systematic review tables ECERS. (PDF)

S6 File Acronyms ECERS. (PDF)

S7 File PRISMA checklist ECERS. (PDF)

S8 File Database ECERS. (ZIP)

## References

[1] Statistics Canada,. Child care: an eight-year profile. The Daily. Canada; 5 Apr 2006. Available: http://www.statcan.gc.ca/daily-quotidien/060405/dq060405a-eng.htm. Accessed 1 Jul 2015.

[2] Fuller B, Holloway S, Liang X. Family selection of child-care centers: the influence of household support, ethnicity and parental practices. Child Dev. 1996;67: 3320–3337.

[3] Whitebook M. Early education quality: higher teacher qualifications for better learning environments—a review of the literature [Internet]. Berkely, CA: Center for The Study of Child Care Employment; 2003 Available: http://www.irle.berkeley.edu/cscce/wp-content/uploads/2003/01/Early_Ed_Quality.pdf

[4] Burchinal MR, Roberts JE, Riggins R, Zeisel SA, Neebe E, Bryant D. Relating quality of center-based child care to early cognitive and language development longitudinally. Child Dev. 2000;71: 338–357. 10.1111/1467-8624.00149 10834469

[5] Burchinal M, Peisner-Feinberg E, Bryant D, Clifford R. Children’s social and cognitive development and child-care quality: testing for differential associations related to poverty, gender, or ethnicity. Appl Dev Sci. 2000;4: 149–165.

[6] Peisner-Feinberg E, Burchinal M, Clifford R, Culkin M, Howes C, Kagan S, et al The relation of preschool child-care quality to children’s cognitive and social developmental trajectories through second grade. Child Dev. 2001;72: 1534–1553. 11699686 10.1111/1467-8624.00364

[7] McCartney K. Effect of quality of day care environment on children’s language development. Dev Psychol. 1984;20: 244–260.

[8] Kamerman S, Gatenio-Gabel S. Early childhood education and care in the united states: an overview of the current policy picture. Int J Child Care Educ Policy. 2007;1: 23–24.

[9] Cryer D, Phillipsen L. Quality details: a close-up look at child care program strengths and weaknesses. Young Child. 1997;52: 51–61.

[10] Helburn SW. Cost, quality and child outcomes in child care centers [Internet]. Denver, CO: University of Colorado at Denver; 1995 Available: http://www.researchconnections.org/childcare/resources/1459

[11] Peisner-Feinberg E., Burchinal MR, Clifford RM, Yazejian N, Culkin M., Zelazo J, et al The children of the cost, quality, and outcomes study go to school: Technical report. Frank Porter Graham Child Dev Cent 1999;

[12] Gordon R, Hofer K, Fujimoto K, Risk N, Kaestner R, Korenman S. Identifying high-quality preschool programs: new evidence on the validity of the Early Childhood Environment Rating-Scale (ECERS-R) in relation to school readiness goals. Early Educ Dev. 2015;26: 1086–1110.

[13] Bryant D, Maxwell K, Taylor K, Poe M, Peisner-Feinberg E, Bernier K. Smart start and preschool child care quality in North Carolina: Change over time and relation to children’s readiness FPG Child Dev Inst 2003;

[14] Sakai L, Whitebook M, Wishard A, Howes C. Evaluating the early childhood environment rating scale (ECERS): assessing differences between the first and revised edition. Early Child Res Q. 2003;18: 427–445.

[15] Burchinal M, Kainz K, Cai K. How well do our measures of quality predict child outcomes? A meta-analysis and coordinated analysis of data from large-scale studies of early childhood settings In: Zaslow M, Martinez-Beck I, Tout K, Halle T, editors. Quality measurement in early childhood settings. Baltimore, MD: Paul H Brookes Publishing; 2011 pp. 11–31. Available: http://www.researchconnections.org/childcare/resources/21062

[16] Copple C, Bredekamp S. Developmentally appropriate practice Washington, DC: National Association for the Education of Young Children; 2009.

[17] Tout K, Starr R, Soli M, Moodie S, Kirby G, Boller K. The Child Care Quality Rating System (QRS) Assessment: Compendium of Quality Rating Systems and Evaluations, OPRE Report [Internet]. Washington, DC: U.S. Department of Health and Human Services, Administration for Children and Families, Office of Planning, Research and Evaluation; 2010 Available: https://www.acf.hhs.gov/sites/default/files/opre/qrs_compendium_final.pdf

[18] La Paro KM, Thomason AC, Lower JK, Kinter-Duffy VL, Cassidy DJ. Examining the definition and measurement of quality in early childhood education: a review of studies using the ECERS-R from 2003 to 2010. Early Child Res Pract. 14: n1.

[19] Caronongan P, Kirby G, Malone L, Boller K. Defining and measuring quality: An in-depth study of five child care quality rating and improvement systems (OPRE Report #2011–29). Washington, DC: U.S. Department of Health and Human Services, Administration for Children and Families, Office of Planning, Research and Evaluation; 2011.

[20] Clifford R, Reszka S, Rossbach H. Reliability and validity of the early childhood environment rating scale Unpublished manuscript [Internet]. Chapel Hill, NC: FPG Child Development Institute; 2010 Available: http://www.ersi.info/PDF/ReliabilityEcers.pdf

[21] Vandell DB, Wolfe B. Childcare quality: Does it matter and does it need to be improved. Dep Health Hum Serv. 2000;

[22] Arnett J. Caregiver interaction scale. Princeton, NJ: Educational Testing Service; 1989.

[23] Pianta R, Karen M, Paro L, Hamre B. Classroom assessment scoring system (CLASS) manual, pre-K Baltimore, MD: Paul H. Brookes Publishing Company; 2008.

[24] Perlman M, Falenchuk O, Fletcher B, McMullen E, Beyene J, Shah P. A systematic review and meta-analysis of a measure of staff/child interaction quality (the classroom assessment scoring system) in early childhood education and care settings and child outcomes. PLOS One. 2016;11: e0167660 doi: 10.1371/journal.pone.0167660 28036333 10.1371/journal.pone.0167660 PMC5201239

[25] Harms T, Clifford R. The early childhood environment rating scale (ECERS) New York, NY: Teachers College Record; 1980.

[26] Harms T, Clifford R, Cryer D. Early Childhood Environment Scale-Revised Edition. 1998.

[27] Bryant D, Burchinal M, Lau L, Sparling J. Family and classroom correlates of head start children’s developmental outcomes. Early Child Res Q. 1994;9: 289–304.

[28] Scarr S, Eisenberg M, Deater-Deckard K. Measurement of quality in child care centers. Early Child Res Q. 1994;9: 131–151.

[29] Zellman G. Assessing the validity of the Qualistar early learning quality rating and improvement system as a tool for improving child-care quality [Internet]. Santa Monica, CA: RAND Education; 2008 Available: http://site.ebrary.com/id/10277634

[30] Whitebook M, Howes C, Phillips D. Who cares? Child Care Teachers and The Quality of Care in America. Final Report National Child Care Staffing Study. Berkely, CA: Child Care Employee Project; 1989 pp. 41–45.

[31] Cassidy D, Hestenes L, Hegde A, Hestenes S, Mims S. Measurement of quality in preschool child care classrooms: an exploratory and confirmatory factor analysis of the early childhood environment rating scale-revised. Early Child Res Q. 2005;20: 345–360.

[32] Cappella E, Aber J, Kim H. Teaching beyond achievement tests: Perspectives from developmental and education scienceIn Gitomer D. H. & Bell C. A. (Eds.). Handbook of research on teaching. Washington, DC: American Educational Research Association; 2016 pp. 249–347.

[33] Mashburn A, Pianta R, Hambre B. Measures of classroom quality in prekindergarten and children’s development of academic, language, and social skills. Child Dev. 2008;79: 732–749. doi: 10.1111/j.1467-8624.2008.01154.x 18489424 10.1111/j.1467-8624.2008.01154.x

[34] Early D, Bryant D, Pianta R. Are teachers’ education, major and credentials related to classroom quality and children’s academic gains in pre-kindergarten? Early Child Res Q. 2006;21: 174–195.

[35] Burchinal M, Howes C, Pianta R, Bryant D, Early D, Clifford R, et al Predicting Child Outcomes at the End of Kindergarten from the Quality of Pre-Kindergarten Teacher–Child Interactions and Instruction. Appl Dev Sci. 2008;12: 140–153.

[36] Cost, Quality and Outcomes Study (CQO). Web site. In: Frank Porter Graham Child Development Institute [Internet]. [cited 8 Jul 2015]. Available: http://fpg.unc.edu/search/apachesolr_search/costqualityoutcome

[37] Early Childhood Longitudinal Program (ECLS). Web site. In: National Center for Education Statistics [Internet]. [cited 1 Jul 2015]. Available: http://nces.ed.gov/ecls/

[38] Effective Pre-School, Primary and Secondary Education (EPPSE). Web site. In: Institute of Education, University College London [Internet]. [cited 1 Jul 2015]. Available: http://www.ioe.ac.uk/research/153.html

[39] Head Start Impact Study (HS). Web site. In: Administration for Children and Families [Internet]. [cited 1 Jul 2015]. Available: http://www.acf.hhs.gov/programs/opre/research/project/head-start-impact-study-and-follow-up

[40] National Center for Early Development & Learning (NCEDL) multi-state study of pre-kindergarten. Web site. In: Frank Porter Graham Child Development Institute [Internet]. [cited 8 Jul 2015]. Available: http://fcd-us.org/resources/pre-kindergarten-11-states-ncedls-multi-state-study-pre-kindergarten-and-study-statewide-e?destination=resources%2Fsearch%3Fpage%3D27

[41] Head Start Family And Child Experiences Survey (FACES) series. Web site. In: Inter-University Consortium for Political and Social Research [Internet]. [cited 1 Jul 2015]. Available: https://www.icpsr.umich.edu/icpsrweb/ICPSR/series/236

[42] Study of Early Child Care and Youth Development (NICHD). Web site. In: U.S. Department of Health and Human Services, National Institutes of Health [Internet]. 2015 [cited 8 Jul 2015]. Available: http://www.nichd.nih.gov/research/supported/seccyd/Pages/datasets.aspx

[43] Wells G, Shea B, O’Connel D, Peterson J, Welch V, Losos M, et al. The Newcastle-Ottawa scale (NOS) for assessing the quality if nonrandomized studies in meta-analyses. In: The Ottawa Hospital Research Institute Web site [Internet]. [cited 1 Jul 2015]. Available: http://www.ohri.ca/programs/clinical_epidemiology/oxford.asp

[44] Howes C, Burchinal M, Pianta R, Bryant D, Early D, Clifford R, et al Ready to learn? Children’s pre-academic achievement in pre-Kindergarten programs. Early Child Res Q. 2008;23: 27–50.

[45] Abreu-Lima I, Leal T, Cadima J, Gamelas A. Predicting child outcomes from preschool quality in Portugal. Eur J Psychol Educ. 2013;28: 399–420.

[46] Kwan C, Sylva K, Reeves B. Day care quality and child development in Singapore. Early Child Dev Care. 1998;144: 69–77.

[47] Moher D, Liberati A, Tetzlaff J, Altman D, The PRISMA Group. Preferred reporting items for systematic reviews and meta-analyses: the PRISMA statement. PLoS Med. 2009;6. PMC3090117 21603045

[48] Henry G, Henderson L, Ponder B, Gordon C, Mashburn A, Rickman D. Report of the findings from the early childhood study: 2001–02 [Internet]. Georgia State University, School of Policy Studies; 2003 p. Atlanta, GA Available: http://eric.ed.gov/?id=ED481261

[49] Mashburn A. Quality of social and physical environments in preschools and children’s development of academic, language, and literacy skills. Appl Dev Sci. 2008;12: 103–127.

[50] Peisner-Feinberg E, Maris C, More at Four Evaluation Team. Evaluation of the North Carolina more at four pre-kindergarten program: children’s longitudinal outcomes and classroom quality in kindergarten [Internet]. Chapel Hill, NC: FPG Child Development Institute; 2006 Available: http://fpg.unc.edu/sites/fpg.unc.edu/files/resources/reports-and-policy-briefs/MAF_Yr4_pt2_full_report.pdf

[51] Jeon H, Langill C, Peterson C, Luze G, Carta J, Atwater J. Children’s individual experiences in early care and education: relations with overall classroom quality and children’s school readiness. Early Educ Dev. 2010;21: 912–939.

[52] Montes G, Hightower A, Brugger L, Moustafa E. Quality child care and socio-emotional risk factors: no evidence of diminishing returns for urban children. Early Child Res Q. 2005;20: 361–372.

[53] Peisner-Feinberg E, Schaaf J. Evaluation of the North Carolina More at Four pre-kindergarten program year 7 report (2007–2008): Performance and progress in the seventh year (2007–2008) [Internet]. Chapel Hill, NC: FPG Child Development Institute. University of North Carolina; 2008 Available: http://www.ncga.state.nc.us/documentsites/committees/JLEOC/Reports%20Received/Archives/2009%20Reports%20Received/More%20At%20Four%20Program%20Review/Year%207%20Performance%20and%20Progress.pdf

[54] Anders Y, Rossbach H, Weinert S, Ebert S, Kuger S, Lehrl S, et al Home and preschool learning environments and their relations to the development of early numeracy skills. Early Child Res Q. 2012;27: 231–244.

[55] Wood J. Methodology for dealing with duplicate study effects in a meta-analysis. Organ Res Methods. 2008;11: 79–95.

[56] Comprehensive meta-analysis. Version 2.0. Engelwood, NJ: Biostat; 2005.

[57] Higgins J, Thompson S, Deeks J, Altman D. Measuring inconsistency in meta-analyses. Br Med J. 2003;327: 557–560. 12958120 10.1136/bmj.327.7414.557 PMC192859

[58] Chang F, Crawford G, Early D, Bryant D, Howes C, Burchinal M, et al Spanish-speaking children’s social and language development in pre-kindergarten classrooms. Early Educ Dev. 2007;18: 243–269.

[59] Dang T, Farkas G, Burchinal M, Duncan G, Vandell D, Li W, et al Preschool center quality and school readiness: Quality main effects and variation by demographic and child characteristics [Internet]. Evanston, IL: Society for Research on Educational Effectiveness; 2011 Available: http://eric.ed.gov/?id=ED519004

[60] Keys T, Farkas G, Burchinal M, Duncan G, Vandell D, Li W, et al Preschool center quality and school readiness: quality effects and variation by demographic and child characteristics. Child Dev. 2013;84: 1171–1190. doi: 10.1111/cdev.12048 23331043 10.1111/cdev.12048 PMC4024382

[61] Reid J, Ready D. High-quality preschool: the socioeconomic composition of preschool classrooms and children’s learning. Early Educ Dev. 2013;24: 1082–1111.

[62] Sabol T, Soliday Hong S, Pianta R, Burchinal M. Can rating pre-k programs predict children’s learning? Science. 2013;341: 845–846. doi: 10.1126/science.1233517 23970684 10.1126/science.1233517

[63] Dotterer AM, Burchinal M, Bryant D, Early D, Pianta RC. Universal and targeted pre-kindergarten programmes: a comparison of classroom characteristics and child outcomes. Early Child Dev Care. 2013;183: 931–950.

[64] Peisner-Feinberg E, Burchinal M. Relations between preschool children’s child-care experiences and concurrent development: the cost, quality, and outcomes study. Merrill-Palmer Q. 1997;43: 451–477.

[65] Burchinal M, Nelson L. Family selection and child care experiences: Implications for studies of child outcomes. Early Child Res Q. 2000;15: 385–411.

[66] Aboud F. Evaluation of an early childhood preschool program in rural Bangladesh. Early Child Res Q. 2006;21: 46–60.

[67] Aboud F, Hossain K. The impact of preprimary school on primary school achievement in Bangladesh. Early Child Res Q. 2011;26: 237–246.

[68] McCartney K, Scarr S, Grajek S, Schwarz J. Environmental differences among day care centers and their effects on children’s development In: Zigler E, Gordon E, editors. Day care: scientific and social policy issues. Boston, MA: Auburn House Publishing Company; 1982.

[69] Phillips D, McCartney K, Scarr S. Child-care quality and children’s social development. Dev Psychol. 1987;23: 537–543.

[70] Chin-Quee D, Scarr S. Lack of early child care effects on school-age children’s social competence and academic achievement. Early Dev Parent. 1994;3: 103–112.

[71] Fiorentino L, Howe N. Language competence, narrative ability, and school readiness in low-income preschool children. Can J Behav Sci Can Sci Comport. 2004;36: 280–294.

[72] Goelman H, Pence A. Children in three types of day care: daily experiences, quality of care and developmental outcomes. Early Child Dev Care. 1988;33: 67–76.

[73] Schliecker E, White D, Jacobs E. The role of day care quality in the prediction of children’s vocabulary. Can J Behav Sci Can Sci Comport. 1991;23: 12–24.

[74] Herrera M, Mathiesen M, Merino J, Recart I. Learning contexts for young children in Chile: process quality assessment in preschool centres. Int J Early Years Educ. 2005;13: 13–27.

[75] Sylva K, Siraj-Blatchford I, Taggart B, Sammons P, Melhuish E, Elliot K, et al Capturing quality in early childhood through environmental rating scales. Early Child Res Q. 2006;21: 76–92.

[76] Pinto A, Pessanha M, Aguiar C. Effects of home environment and center-based child care quality on children’s language, communication, and literacy outcomes. Early Child Res Q. 2013;28: 94–101.

[77] Lyon M, Canning P. The Atlantic day care study. Halifax: Mount Saint Vincent University; 1995.

[78] Howes C, Sakai L, Shinn M, Phillips D, Galinsky E, Whitebook M. Race, social class, and maternal working conditions as influences on children’s development. J Appl Dev Psychol. 1995;16: 107–124.

[79] Barnett WS, Yarosz DJ, Thomas J, Jung K, Blanco D. Two-way and monolingual English immersion in preschool education: An experimental comparison. Early Child Res Q. 2007;22: 277–293.

[80] Kontos S. Child care quality, family background, and children’s development. Early Child Res Q. 1991;6: 249–262.

[81] Gordon R, Fujimoto K, Kaestner R, Korenman S, Abner K. An assessment of the validity of the ECERS-R with implications for measures of child care quality and relations to child development. Dev Psychol. 2013;49: 146–160. doi: 10.1037/a0027899 22468567 10.1037/a0027899 PMC3681422

[82] McWayne C, Fantuzzo J, McDermott P. Preschool competency in context: an investigation of the unique contribution of child competencies to early academic success. Dev Psychol. 2004;40: 633–645. doi: 10.1037/0012-1649.40.4.633 15238049 10.1037/0012-1649.40.4.633

[83] Clawson C, Luze G. Individual experiences of children with and without disabilities in early childhood settings. Top Early Child Spec Educ. 2008;28: 132–147.

[84] Seppanen P, Godin K, Metzger J, Bronson M, Cichon D. Observational Study of Early Childhood Programs. Final Report. Volume II: Chapter 1- Funded Early Childhood Programs. [Internet]. Washington, DC: U.S. Department of Education; 1993 Available: http://files.eric.ed.gov/fulltext/ED366468.pdf

[85] Weiland C, Ulvestad K, Sachs J, Yoshikawa H. Associations between classroom quality and children’s vocabulary and executive function skills in an urban public prekindergarten program. Early Child Res Q. 2013;28: 199–209.

[86] West J, Malone L, Hulsey L, Aikens N, Tarullo L. ACF-OPRE report: Head Start children go to kindergarten [Internet]. Washington, DC: U.S. Department of Health and Human Services, Administration for Children and Families, Office of Planning, Research and Evaluation; 2010 Available: http://www.acf.hhs.gov/sites/default/files/opre/hs_kindergarten.pdf

[87] Zill N, Resnick G, Kim K, O’Donnell K, Sorongon A, McKey R, et al Head start FACES 2000: a whole-child perspective on program performance [Internet]. Washington DC: Administration for Children and Families, U.S. Department of Health and Human Services; 2003 Available: http://www.acf.hhs.gov/sites/default/files/opre/faces00_4thprogress.pdf

[88] Burchinal M, Roberts J, Zeisel S, Hennon E, Hooper S. Social risk and protective child, parenting, and child care factors in early elementary school years. Parenting. 2006;6: 79–113.

[89] Dickinson D, Tabors P, editors. Beginning Literacy with Language: Young Children Learning at Home and School. Baltimore, MD: Brookes Publishing; 2001.

[90] Henry G, Rickman D, Ponder B, Henderson L, Mashburn A, Gordon C. The Georgia early childhood study, 2001–2004, final report [Internet]. Atlanta, GA: Andrew Young School of Policy Studies, Georgia State University; 2005 Available: http://citeseerx.ist.psu.edu/viewdoc/download?

[91] Hindman AH, Skibbe LE, Miller A, Zimmerman M. Ecological contexts and early learning: Contributions of child, family, and classroom factors during Head Start, to literacy and mathematics growth through first grade. Early Child Res Q. 2010;25: 235–250.

[92] Peisner-Feinberg E, Schaaf J, LaForett D. Children’s growth and classroom experiences in Georgia’s pre‐k program: findings from the 2011–2012 evaluation study [Internet]. Chapel Hill, NC: FPG Child Development Institute; 2013 Available: http://files.eric.ed.gov/fulltext/ED541933.pdf

[93] Sabol T, Pianta R. Do standard measures of preschool quality used in statewide policy predict school readiness? Educ Finance Policy. 2014;9: 116–164.

[94] Zill N, Resnick G, Kim K, O’Donnell K, Sorongon A, Ziv Y, et al Head start performance measures center family and child experiences survey (FACES 2000): technical report [Internet]. Washington, DC: Office of Planning, Research and Evaluation, Administration for Children and Families, U.S. Department of Health and Human Services; 2006 Available: http://www.acf.hhs.gov/sites/default/files/opre/tech2k_final2.pdf

[95] Aikens N, Moiduddin E, Xue Y, Tarullo L, West J. Data tables for child outcomes and classroom quality in FACES 2009 report. OPRE report 2012- 37b [Internet]. Washington, DC: Administration for Children and Families; 2012 pp. 1–200. Available: http://www.acf.hhs.gov/sites/default/files/opre/data_tables_for_child_outcomes_and_classroom_quality_in_faces_2009.pdf

[96] Aikens N, Tarullo L, Hulsey L, Ross C, West J, Xue Y. A Year in Head Start: Children, Families and Programs. ACF-ORPRE Report. Adm Child Fam. 2010; Available: http://eric.ed.gov/?id=ED517213

[97] Assel M, Landry S, Swank P. Are early childhood classrooms preparing children to be school ready? The circle teacher behavior rating scale In: Justice L, Vukelic C, editors. Achieving Excellence in Preschool Literacy Instruction. New York, NY: Guilford Press; 2008 pp. 120–135.

[98] Dunn L. Proximal and distal features of day care quality and children’s development. Early Child Res Q. 1993;8: 167–192.

[99] Dunn L, Beach S, Kontos S. Quality of the literacy environment in day care and children’s development. J Res Child Educ. 1994;9: 24–34.

[100] Epstein A. Training for quality: improving early childhood programs through systematic inservice training Ypsilanti, Michigan: High/Scope Press; 1993.

[101] Jackson B, Larzelere R, St. Clair L, Corr M, Fichter C, Egertson H. The impact of HeadsUp! reading on early childhood educators’ literacy practices and preschool children’s literacy skills. Early Child Res Q. 2006;21: 213–226.

[102] Le V, Schaack D, Setodji C. Identifying baseline and ceiling thresholds within the qualistar early learning quality rating and improvement system. Early Child Res Q. 2015;30: 215–226. doi: 10.1016/j.ecresq.2014.03.003 25530668 10.1016/j.ecresq.2014.03.003 PMC4269260

[103] Moller A, Forbes-Jones E, Hightower A, Friedman R. The developmental influence of sex composition in preschool classrooms: boys fare worse in preschool classrooms with more boys. Early Child Res Q. 2008;23: 409–418.

[104] Moller A, Forbes-Jones E, Hightower A. Classroom age composition and developmental change in 70 urban preschool classrooms. J Educ Psychol. 2008;100: 741–753.

[105] Peisner-Feinberg E, Schaaf J. Evaluation of the North Carolina More at Four pre-kindergarten program year 6 report (July 1, 2006-June 30, 2007): children’s longitudinal outcomes and program quality over time (2003–2007) [Internet]. Chapel Hill, NC: FPG Child Development Institute; 2008 Available: http://ea.niusileadscape.org/docs/FINAL_PRODUCTS/LearningCarousel/maf_Yr6_rpt.pdf

[106] Peisner-Feinberg E, Schaaf J, The More at Four Evaluation Team. Children’s outcomes & program quality in the fifth year. Evaluation of the North Carolina more at four pre-kindergarten program, year 5 report (July 1, 2005-June 30, 2006) [Internet]. Chapel Hill, NC: FPG Child Development Institute, University of North Carolina; 2007 Available: http://eric.ed.gov/?id=ED499809

[107] Auger A, Farkas G, Burchinal M, Duncan G, Vandell D. Preschool center care quality effects on academic achievement: an instrumental variables analysis. Dev Psychol. 2014;50: 2559–2571. doi: 10.1037/a0037995 25437755 10.1037/a0037995

[108] Hestenes L, Kintner-Duffy V, Wang Y, La Paro K, Mims S, Crosby D, et al Comparisons among quality measures in child care settings: understanding the use of multiple measures in North Carolina’s QRIS and their links to social-emotional development in preschool children. Early Child Res Q. 2015;30: 199–214.

[109] Cleveland G. ECEC Policy in Canada [Internet]. Toronto, ON: Department of Management at the University of Toronto Scarborough; 2015 Available: https://www.policyalternatives.ca/sites/default/files/uploads/publications/National%20Office/2015/09/osos120_ECEC_Policy.pdf

[110] OECD. Encouraging Quality in ECEC (Research Brief) [Internet]. Paris: Organization for Economic Co-operation and Development; Available: http://www.oecd.org/education/school/48483409.pdf

[111] OECD. Starting Strong I: Early Childhood Education and Care. Paris: Organization for Economic Co-operation and Development; 2001.

[112] OECD. Starting Strong II: Early Childhood Education and Care. Paris: Organization for Economic Co-operation and Development; 2006.

[113] Phillips D, Adams G. Child care and our youngest children. Future Child. 2001;11: 35–51. 11712454

[114] Howes C, Smith E. Relations among child care quality, teacher behavior, children’s play activities, emotional security, and cognitive activity in child care. Early Child Res Q. 1995;10: 381–404.

