# Supplementary material for: The relationship between the Early Childhood Environment Rating Scale and its revised form and child outcomes: A systematic review and meta-analysis
Source: PLoS One. 2017 Jun 6;12(6):e0178512. doi: 10.1371/journal.pone.0178512 (PMC5461062; doi:10.1371/journal.pone.0178512)
Supplement: S1 File — (PDF) [file pone.0178512.s001.pdf]

The Relationship between the Early Childhood Environment Rating Scale and its Revised Form and Child Outcomes: a Systematic Review and Meta-Analysis

Supplemental Information 1

Searches were conducted from the earliest date possible until July 3, 2015. The following search terms were used for the 3 global and 3 individual electronic searches:

**Table A**. Syntax for Records identified in ERIC: Global Search with Multiple Quality Indicators
(((("child care" OR "child care center" OR "child care centre" OR "child development center" OR "child development centre" OR "early childhood education" OR "nursery school" OR "preschool children" OR "preschool education" OR "day care centre" OR "day care center") NOT rtype.exact("150 Speeches/meeting Papers" OR "052 Guides - Classroom - Teacher" OR "160 Tests/questionnaires" OR "043 Dissertations/theses - Practicum Papers" OR "021 Collected Works - Proceedings" OR "041 Dissertations/theses - Doctoral Dissertations" OR "040 Dissertations/theses" OR "042 Dissertations/theses - Masters Theses")) AND (("child care" NEAR/3 quality OR "classroom assessment scoring system" OR "early childhood environment rating system" OR "ECERS" OR "caregiver interaction scale" OR "caregiver child relationship" OR "caregiver training" OR "childcare givers" OR " class size" OR "classroom environment" OR "developmentally appropriate practices" OR "preschool evaluation" OR "preschool teacher" OR "teacher background" OR "teacher certification" OR "teacher characteristics" OR "teacher education" OR "teacher student" NEAR/3 ratio OR "teacher student" NEAR/3 interaction* OR "teacher qualifications" OR "teaching experience") NOT rtype.exact("150 Speeches/meeting Papers" OR "052 Guides - Classroom - Teacher" OR "160 Tests/questionnaires" OR "043 Dissertations/theses - Practicum Papers" OR "021 Collected Works - Proceedings" OR "041 Dissertations/theses - Doctoral Dissertations" OR "040 Dissertations/theses" OR "042 Dissertations/theses - Masters Theses")) AND (("academic achievement" OR "behavior development" OR "Behavior development" OR "child development" OR "child health" OR "child language" OR "cognitive ability" OR "student development" OR "school readiness" OR "vocabulary development") NOT rtype.exact("150 Speeches/meeting Papers" OR "052 Guides - Classroom - Teacher" OR "160 Tests/questionnaires" OR "043 Dissertations/theses - Practicum Papers" OR "021 Collected Works - Proceedings" OR "041 Dissertations/theses - Doctoral Dissertations" OR "040 Dissertations/theses" OR "042 Dissertations/theses - Masters Theses"))) NOT rtype.exact("150 Speeches/meeting Papers" OR "052 Guides - Classroom - Teacher" OR "160 Tests/questionnaires" OR "043 Dissertations/theses - Practicum Papers" OR "021 Collected Works - Proceedings" OR "041 Dissertations/theses - Doctoral Dissertations" OR "040 Dissertations/theses" OR "042 Dissertations/theses - Masters Theses")) NOT (rtype.exact("150 Speeches/meeting Papers" OR "052 Guides - Classroom - Teacher" OR "160 Tests/questionnaires" OR "043 Dissertations/theses - Practicum Papers" OR "021 Collected Works - Proceedings" OR "041 Dissertations/theses - Doctoral Dissertations" OR "040 Dissertations/theses" OR "042 Dissertations/theses - Masters Theses") NOT rtype.exact("150 Speeches/meeting Papers" OR "052 Guides - Classroom - Teacher" OR "160 Tests/questionnaires" OR "043 Dissertations/theses - Practicum Papers" OR "021 Collected Works - Proceedings" OR "041 Dissertations/theses - Doctoral Dissertations" OR "040 Dissertations/theses" OR "042 Dissertations/theses - Masters Theses"))

The Relationship between the Early Childhood Environment Rating Scale and its Revised Form and Child Outcomes: a Systematic Review and Meta-Analysis

Supplemental Information 1

**Table B.** Syntax for Records identified in ERIC: Search for ECERS Alone
(((("child care" OR "child care center" OR "child care centre" OR "child development center" OR "child development centre" OR "early childhood education" OR "nursery school" OR "preschool children" OR "preschool education" OR "day care centre" OR "day care center") NOT rtype.exact("150 Speeches/meeting Papers" OR "052 Guides - Classroom - Teacher" OR "160 Tests/questionnaires" OR "043 Dissertations/theses - Practicum Papers" OR "021 Collected Works - Proceedings" OR "041 Dissertations/theses - Doctoral Dissertations" OR "040 Dissertations/theses" OR "042 Dissertations/theses - Masters Theses")) AND (("early childhood environment rating system" OR "ECERS") NOT rtype.exact("150 Speeches/meeting Papers" OR "052 Guides - Classroom - Teacher" OR "160 Tests/questionnaires" OR "043 Dissertations/theses - Practicum Papers" OR "021 Collected Works - Proceedings" OR "041 Dissertations/theses - Doctoral Dissertations" OR "040 Dissertations/theses" OR "042 Dissertations/theses - Masters Theses")) AND (("academic achievement" OR "behavior development" OR "Behavior development" OR "child development" OR "child health" OR "child language" OR "cognitive ability" OR "student development" OR "school readiness" OR "vocabulary development") NOT rtype.exact("150 Speeches/meeting Papers" OR "052 Guides - Classroom - Teacher" OR "160 Tests/questionnaires" OR "043 Dissertations/theses - Practicum Papers" OR "021 Collected Works - Proceedings" OR "041 Dissertations/theses - Doctoral Dissertations" OR "040 Dissertations/theses" OR "042 Dissertations/theses - Masters Theses"))) NOT rtype.exact("150 Speeches/meeting Papers" OR "052 Guides - Classroom - Teacher" OR "160 Tests/questionnaires" OR "043 Dissertations/theses - Practicum Papers" OR "021 Collected Works - Proceedings" OR "041 Dissertations/theses - Doctoral Dissertations" OR "040 Dissertations/theses" OR "042 Dissertations/theses - Masters Theses")) NOT (rtype.exact("150 Speeches/meeting Papers" OR "052 Guides - Classroom - Teacher" OR "160 Tests/questionnaires" OR "043 Dissertations/theses - Practicum Papers" OR "021 Collected Works - Proceedings" OR "041 Dissertations/theses - Doctoral Dissertations" OR "040 Dissertations/theses" OR "042 Dissertations/theses - Masters Theses") NOT rtype.exact("150 Speeches/meeting Papers" OR "052 Guides - Classroom - Teacher" OR "160 Tests/questionnaires" OR "043 Dissertations/theses - Practicum Papers" OR "021 Collected Works - Proceedings" OR "041 Dissertations/theses - Doctoral Dissertations" OR "040 Dissertations/theses" OR "042 Dissertations/theses - Masters Theses"))

The Relationship between the Early Childhood Environment Rating Scale and its Revised Form and Child Outcomes: a Systematic Review and Meta-Analysis

Supplemental Information 1

Table C. Syntax for Records identified in Medline: Global Search with Multiple Quality Indicators & Individual Search for ECERS Alone

Set	Search Statement
1.	child care.mp. or exp Child Care/
2.	exp Child Day Care Centers/ or day care cent*.mp.
3.	exp Schools, Nursery/ or nursery school*.mp.
4.	preschool education.mp.
5.	exp "Early Intervention (Education)"/
6.	exp Child, Preschool/ or preschool student*.mp.
7.	pre-school student*.mp.
8.	child development.mp. or exp Child Development/
9.	intelligence.mp. or exp Intelligence/
10.	exp Language/ or language.mp.
11.	exp Health/ or health.mp.
12.	academic achievement*.mp.
13.	exp Vocabulary/ or vocabulary.mp.
14.	cognitive development.mp.
15.	emotional development.mp.
16.	cortisol.mp. or exp Hydrocortisone/
17.	chicken pox.mp. or exp Chickenpox/
18.	virus.mp. or exp Viruses/
19.	program development.mp. or exp Program Development/
20.	inservice training.mp. or exp Inservice Training/
21.	professional development.mp.
22.	teach* education.mp.
23.	group size.mp.
24.	classroom environment.mp.
25.	school environment.mp.
26.	teach* characteristics.mp.
27.	teach* qualification*.mp.
28.	teacher student interaction.mp.
29.	student teacher interaction.mp.
30.	ECERS.mp.
31.	classroom assessment scoring system.mp.
32.	early childhood environment rating scale.mp.
33.	caregiver interaction scale.mp.
34.	ratios.mp.
35.	preschool child*.mp.
36.	social development.mp.
37.	(teach* adj3 experience).mp. [mp=title, abstract, original title, name of substance word, subject heading word, keyword heading word, protocol supplementary concept, rare disease supplementary concept, unique identifier]
38.	1 or 2 or 3 or 4 or 5 or 6 or 7 or 35

Table C. Syntax for Records identified in Medline: Global Search with Multiple Quality Indicators & Individual Search for ECERS Alone

39.	exp Otitis Media/ or ear infection*.mp.
40.	8 or 9 or 10 or 11 or 12 or 13 or 14 or 15 or 16 or 17 or 18 or 36 or 39
41.	19 or 20 or 21 or 22 or 23 or 24 or 25 or 26 or 27 or 28 or 29 or 30 or 31 or 32 or 33 or 34 or 37
42.	38 and 40 and 41
43.	limit 42 to (english language and humans and ("preschool child (2 to 5 years)" or "child (6 to 12 years)") and (journal article or meta analysis or randomized controlled trial or "research support, american recovery and reinvestment act" or research support, nih, extramural or research support, nih, intramural or research support, non us gov't or research support, us gov't, non phs or research support, us gov't, phs or systematic reviews or technical report))
44.	**Individual Search for ECERS Alone**<br>30 and 32 and 38 and 40

The Relationship between the Early Childhood Environment Rating Scale and its Revised Form and Child Outcomes: a Systematic Review and Meta-Analysis

Supplemental Information 1

Table D. Syntax for Records identified in PsycINFO: Global Search with Multiple Quality Indicators & Individual Search for ECERS Alone

Set	Search Statement
1.	child care.mp. or exp Child care/
2.	day care cent*.mp.
3.	exp Nursery Schools/ or nursery school*.mp.
4.	preschool education.mp. or exp preschool education/
5.	project Head Start.mp. or exp Project Head Start/
6.	exp Preschool Students/ or preschool student*.mp.
7.	1 or 2 or 3 or 4 or 5 or 6
8.	exp Early Childhood Development/
9.	exp school readiness/
10.	exp academic achievement/
11.	exp psychological development/
12.	exp health/
13.	child outcome*.mp. [mp=title, abstract, heading word, table of contents, key concepts, original title, tests & measures]
14.	8 or 9 or 10 or 11 or 12 or 13
15.	exp "Quality of Services"/ or quality of service*.mp.
16.	professional development.mp. or exp Professional Development/
17.	teacher education.mp. or exp Teacher Education/
18.	inservice teacher education.mp. or exp Inservice Teacher Education/
19.	group size.mp. or exp Group size/
20.	classroom environment.mp. or exp Classroom Environment/
21.	school environment.mp. or exp School Environment/
22.	teacher characteristic*.mp. or exp Teacher Characteristics/
23.	teacher student interaction.mp. or exp Teacher Student Interaction/
24.	program evaluation.mp. or exp Program Evaluation/
25.	(child care adj3 quality).mp. [mp=title, abstract, heading word, table of contents, key concepts, original title, tests & measures]
26.	Classroom Assessment Scoring System.mp. [mp=title, abstract, heading word, table of contents, key concepts, original title, tests & measures]
27.	ECERS.mp. [mp=title, abstract, heading word, table of contents, key concepts, original title, tests & measures]
28.	ratio*.mp. [mp=title, abstract, heading word, table of contents, key concepts, original title, tests & measures]
29.	Caregiver Interaction Scale.mp. [mp=title, abstract, heading word, table of contents, key concepts, original title, tests & measures]
30.	(teacher adj3 experience).mp. [mp=title, abstract, heading word, table of contents, key concepts, original title, tests & measures]
31.	15 or 16 or 17 or 18 or 19 or 20 or 21 or 22 or 23 or 24 or 25 or 26 or 27 or 28 or 29 or 30
32.	7 and 14 and 31
33.	**Individual Search for ECERS Alone**<br>7 and 14 and 27
