# Supplementary material for: The relationship between the Early Childhood Environment Rating Scale and its revised form and child outcomes: A systematic review and meta-analysis
Source: PLoS One. 2017 Jun 6;12(6):e0178512. doi: 10.1371/journal.pone.0178512 (PMC5461062; doi:10.1371/journal.pone.0178512)
Supplement: S2 File — (PDF) [file pone.0178512.s002.pdf]

The Relationship between the Early Childhood Environment Rating Scale and its Revised Form and Child Outcomes: a Systematic Review and Meta-Analysis

Supplemental Information 2
Formulas for Converting Statistics to r for Meta Analyses

Conversion to *t*, *d* or *r* through *t*-statistic

$$t = \frac{B}{SE(B)}$$

$$d = \frac{2t}{\sqrt{n-1}}$$

$$r = \frac{d}{\sqrt{d^2 + 4}}$$

where *B* is an unstandardized regression coefficient, *SE(B)* is its standard error, *d* is its standardized mean difference, *r* is the correlation effect size, and *n* is sample size.
