# Supplementary material for: The relationship between the Early Childhood Environment Rating Scale and its revised form and child outcomes: A systematic review and meta-analysis
Source: PLoS One. 2017 Jun 6;12(6):e0178512. doi: 10.1371/journal.pone.0178512 (PMC5461062; doi:10.1371/journal.pone.0178512)
Supplement: S3 File — (PDF) [file pone.0178512.s003.pdf]

The Relationship between the Early Childhood Environment Rating Scale and its Revised Form and Child Outcomes: a Systematic Review and Meta-Analysis

Supplemental Information 3

Description of Studies Meeting Inclusion Criteria[a]

Study[b]	Characteristics	Quality Measures M(SD)[c]	Outcome Measures M(SD)[d]	Covariates
Aboud 2006[1,V]	**Publication:** Journal (ECRQ)<br>**Design:** Cross-Sectional<br>**Country:** Bangladesh<br>**Sample size:** class 22; child 213 (analyses at classroom level)<br>**% Female:** 54<br>**Mean age:** 65.9<br>**Ethnicity:** NR<br>**Mean maternal education:** NR<br>**Mean household income:** NR<br>**Child Care Type**: Preschool program	ECERS-R Total Score 2.9 (0.45)	School Readiness 21.75 (5.5)<br>WPPSI-III-Vocab 8.1 (1.8)<br>WPPSI-III-Matrix 5.24 (2.2)<br>WPPSI-III-Similarities 9/22 (1.5)	**Statistics Extracted:** Pearson's Correlation<br>**Covariates:**none
Aboud 2011[2,V]	**Publication:** Journal (ECRQ)<br>**Design:** Longitudinal<br>**Country:** Bangladesh<br>**Sample size:** class 30; child 92<br>**% Female:** NR<br>**Mean age:** NR<br>**Ethnicity:** NR<br>**Mean maternal education:** NR<br>**Mean household income:** NR<br>**Child Care Type**: Preschool, primary preschool	ECERS-R Total Score (“Parents and Staff” subscale scale not included) 4.03 (0.39)	Reading 80.8 (26.4)<br>Writing 85.6 (25.1)<br>Written Math 61.4 (34.8)	**Statistics Extracted:** Beta<br>**Covariates:**NR
Abreu-Lima 2013[3,m]	**Publication**: Journal (EJPE)<br>**Design**: Cross-Sectional<br>**Country**: Portugal<br>**Sample size:** class 60; child, range by analyses 177-215<br>**% Female:** 47.4<br>**Mean age:** 67<br>**Ethnicity:** NA<br>**Mean maternal education**: 9.65<br>**Mean household income**: NR<br>**Child Care Type**: Preschool classroom	ECERS-R 3.44 (0.72) (may have used Portuguese version)	CBI- Attention – 3.74 (0.84)<br>CBI- Sociability – 4.04 (0.65)<br>CBI- Considerateness – 3.59 (0.73)<br>CLAFL – Letter ID<br>Cross-Linguistic 5.91 (6.61)<br>Rote Counting 34.91 (22.84)<br>Numbers 33.00 (24.30)<br>PAT – 3.04 (1.59)<br>PPVT-R – 41.69(13.88)<br>Story & Print – 7.00 (3.26)<br>WPPSI-R Math – 7.69 (2.84)	**Statistics:** Pearson’s Correlation, B, SE<br>**Covariates:** age, gender, mother’s education, ECERS-R x mother’s education
Aikens 2010[4, m, B]<br>(2nd doc. Hulsey 2010[5])	**Publication:** Report<br>**Design:** Longitudinal<br>**Data set:** FACES 2006<br>**Country:** United States<br>**Sample size:** class 410; child, range by analysis 2140-2931<br>**%Female:** 49.8<br>**Mean age:** NR<br>**Ethnicity:** C23%, B33%, A2%, H35%, M5%, O2%	ECERS-R Total Score 4.6 (1.2) (no data)<br>ECERS-R Teaching & Interactions 4.08 (NR)	ECLS-Math 9.7 (3.19)<br>PPVT-4 107.9 (16.27)<br>Problem Behaviors 6.42 (0.26)<br>Social Skills 17.3 (0.21)<br>WJ-III-AP 390.3 (31.34)<br>WJ-III-LWI 323.5 (25.76)	**Statistics Extracted:** Beta, Effect Size<br>**Covariates:**child/family level - pretest score, age, gender, ethnicity, language, poverty, maternal education, maternal depressive symptoms; classroom level - full time class, peer social abilities, variation of peer abilities, peer abilities (PPVT), variation of peer abilities, DAP attitudes, teacher education; program level – SES, % ELL, program

Description of Studies Meeting Inclusion Criteria[a]				
**Study**[b]	**Characteristics**	**Quality Measures M(SD)**[c]	**Outcome Measures M(SD)**[d]	**Covariates**
	**Mean maternal education:** NR<br>**Mean household income:** $19,191<br>**Child Care Type**: Head Start			curriculum package, teacher turnover, teacher salary
Aikens 2012[6, m, M]<br>(2nd doc. Moiduddin 2012[7])	**Publication**: Report<br>**Design**: Longitudinal<br>**Dataset:** FACES 2009<br>**Country**: United States<br>**Sample size:** class 391; child, range by analyses 1354-1922<br>**% Female:** 49.8<br>**Mean age:** 36-48 mo.<br>**Ethnicity:** C45%, B66%, H73%, A3%, M11%<br>**Mean maternal education**: NR<br>**Mean household income**: NR<br>**Child Care Type**: Head Start	ECERS-R Teaching & Interactions 4.7 (0.1)	**Age 3/Age 4**<br>ECLS-B 49.1 (20.1) / 66 (23.7)<br>BPI-PB 4.7 (.20) / 3.9 (.20)<br>ECLS-K-PB 1.8 (0.0) / 2.0 (0.1)<br>EOWPVT 85.2 (14.8) / 83.9 (14.3)<br>PPVT-4 90.8 (14.6) / 91 (15.)<br>SS/CBS 16.5 (0.2) / 17.9 (0.2)<br>SS/CBS/PALS 12.2 (0.1) / 12.6 (0.1)<br>WJ-III-AP 93.6 (14.7) / 91.2 (15.2)<br>WJ-III-LWI 104.4 (19.1) / 99.3 (14.4)<br>WJ-III-S 97.5 (14) / 97.4 (14.6)	**Statistics Extracted:** B, SE, Effect Size<br>**Covariates:**<u>child/family level</u> – pretest scores, child age at assessment, gender, ethnicity, language, household poverty ratio, maternal education, maternal depressive symptoms, time interval between the fall and spring assessments; <u>program level</u> – SES, percent DLLs, percent using curriculum and assessment from the same package, teacher turnover, program mean salary
Anders 2012[8]	**Publication:** Journal (ECRQ)<br>**Design:** Longitudinal<br>**Data set:** BiKS Project<br>**Country:** Germany<br>**Sample size:** class 97; child 532<br>**% Female:** 48.12<br>**Mean age:** 37<br>**Ethnicity:** 100% German<br>**Mean maternal education:** NR<br>**Mean household income:** NR<br>**Child Care Type**: Pre-K program	ECERS-R Total Score 3.73 (0.58)	K-ABC-Arithmetic 15.08 (3.74)	**Statistics Extracted:** B, SE<br>**Covariates:** pretest, age, gender, language (German/other), highest socioeconomic status in the family (SES), maternal education, age at entry to preschool, HLE-literacy, HLE-numeracy
Assel 2008[9]	**Publication:** Book Chapter<br>**Design:** Longitudinal<br>**Data set:** PCER<br>**Country:** United States<br>**Sample size:** class 39; child, range by analysis 192-194<br>**% Female:** NR<br>**Mean age:** NR<br>**Ethnicity:** NR<br>**Mean maternal education:** NR<br>**Mean household income:** NR<br>**Child Care Type**: 50% Head Start	ECERS-R Total Score NR	PPVT-III NR<br>Early Reading Ability NR<br>WJ-III-LWI NR	**Statistics Extracted:** df<br>**Covariates:** none
Auger 2014[10, m, U]	**Publication**: Journal (DP)<br>**Design:** Longitudinal<br>**Dataset:** PCER<br>**Country**: United States<br>**Sample size:** centers 320; child, range by analyses 2580-2670	ECERS-R Teaching and Interactions 4.79 (1.42)<br>ECERS-R Provision for Learning 4.00 (1.08)	PPVT 93.25 (15.00)<br>WJ- LWI 103.31 (13.87)<br>WJ- AP 96.21 (13.50)	**Statistics:** B, SE<br>**Covariates:** <u>child/family level</u>– pretest scores, age, gender, ethnicity, mother's (a) age, (b) education, (c) marital status, (d) employment status, welfare aid, annual income

The Relationship between the Early Childhood Environment Rating Scale and its Revised Form and Child Outcomes: a Systematic Review and Meta-Analysis

Description of Studies Meeting Inclusion Criteria[a]				
**Study**[b]	**Characteristics**	**Quality Measures M(SD)**[c]	**Outcome Measures M(SD)**[d]	**Covariates**
	**% Female:** 48.5<br>**Mean age:** 54.27 mo.<br>**Ethnicity:** C34%, B43%, A1%, H15.5%, O6.5%<br>**Mean maternal education**: 12.89<br>**Mean household income**: 30.17 (thousands)<br>**Child Care Type**: Preschool program			
Barnett 2007[11]<br>Whole Sample[m]<br>Sample A:<br>Spanish	**Publication:** Journal (ECRQ)<br>**Design:** Longitudinal<br>**Country:** United States<br>**Sample size A:** class 36; child, range by analysis 128-131<br>**Sample size B:** class 36; child 74<br>**% Female:** 65.5<br>**Mean age:** NR<br>**Ethnicity:** C7.5%, B13%, H76.3%, M2.3%<br>**Mean maternal education:** NR<br>**Mean household income:** NR<br>**Child Care Type**: Preschool program	ECERS-R Total Score NR	Alphabet Recognition-English NR<br>Alphabet Recognition-Spanish NR<br>WM-R-AP NR<br>WM-R-PV NR<br>Phoneme Deletion-English NR<br>Phoneme Deletion-Spanish NR<br>PPVT-III NR<br>Rhyme Recognition-English NR<br>Rhyme Recognition-Spanish NR<br>TVIP NR<br>WJ-R-AP NR<br>WJ-R-PV NR	**Statistics Extracted:** B, SE<br>**Covariates:**pretest score, age, gender, language, teacher education (Has a MA), treatment (TWI or EI)
Bryant 1994[12]<br>Sample A: Suspect Homes[m]<br>Sample B: NonSuspectHomes[m]	**Publication:** Journal (ECRQ)<br>**Design:** Longitudinal<br>**Country:** United States<br>**Sample size A:** class 32; child 40<br>**Sample size B:** class 32; child 99<br>**% Female:** 52<br>**Mean age:** 57<br>**Ethnicity:** C8%, B86%, A1%, H2%, M2%<br>**Mean maternal education:** 12.3 years<br>**Mean household income:** NR<br>**Child Care Type**: Head Start	ECERS Total Score 4.24 (0.46)	ASBI-Comply 42.4 (11)<br>ASBI-Disrupt 17.8 (7.9)<br>ASBI-Express 61.8 (10)<br>K-ABC-Achievement 85.8 (9.6)<br>K-ABC-Mental Processing 92.1 (13)<br>PSI-R 16.9 (6.1)<br>VABS-Communication 83.9 (12.9)	**Statistics Extracted:** B, SE<br>**Covariates:** Home Screening Questionnaire (HSQ), ECERS X HSQ
Bryant 2003[13]<br>Whole Sample[m]<br>Sample A:Males<br>Sample B:Females	**Publication:** Report<br>**Design:** Longitudinal<br>**Country:** United States<br>**Sample size:** class NR; child 512 (Sample A n=260, Sample B n=252)<br>**% Female:** 49.2<br>**Mean age:** NR<br>**Ethnicity:** C54.7%, B32.4%, A2.2%, H2%, O8.8%<br>**Mean maternal education:** NR<br>**Mean household income:** NR<br>**Child Care Type**: Mixed (preschool, Head Start)	ECERS Total Score ("Parents and Staff" subscale scale not included) NR	Counting One-to-One 18.8 (12.51)<br>Letter ID 13.5 (9.93)<br>PPVT-III 94.9 (14.85)<br>SSRS-SS 101.7 (13.46)<br>SSRS-PB 103.3 (14.64)<br>Story & Print-Print Awareness 0.4 (0.5)<br>Story & Print-Book Knowledge 2.7 (1.41)<br>Story & Print-Comprehension 0.7 (0.44)<br>WJ-R-AP 93.9 (15.48)	**Statistics Extracted:** B, SE, Effect Size<br>**Covariates:** gender, ethnicity, subsidy, ECERS X gender, ECERS X ethnicity, ECERS X subsidy
Burchinal, Nelson 2000[14,D]	**Publication:** Journal (ECRQ)<br>**Design:** Longitudinal	ECERS Total Score 4.25 (1.03)	PPVT-R 93.59 (18.48)	**Statistics Extracted:** Pearson's Correlation, Partial Correlation , B,

Description of Studies Meeting Inclusion Criteria[a]				
Study[b]	Characteristics	Quality Measures M(SD)[c]	Outcome Measures M(SD)[d]	Covariates
	**Data set:** CQO<br>**Country:** United States<br>**Sample size:** class NR; child 757<br>**% Female:** 48.9<br>**Mean age:** 48.4<br>**Ethnicity:** C67.9%, B15.9%, H4.6%, O11.6%<br>**Mean maternal education:** 14.22<br>**Mean household income:** NR<br>**Child Care Type**: Centres			SE<br>**Covariates:** state, ethnicity, gender, teacher responsiveness, child centered, CIS, group size, ratio, teacher education
Burchinal, Peisner 2000[15]<br>Sample A:CQO[m, D]<br>Sample B:<br>North Carolina Head Start[m]<br>Sample C:<br>North Carolina Public[m]	**Publication:** Journal (ADS)<br>**Design:** Cross-Sectional<br>**Data set:** CQO<br>**Country:** United States<br>**Sample size:** class 177; child 811<br>**% Female:** 48<br>**Mean age:** 52.2<br>**Ethnicity:** C68%, B15%, A4%, H6%, M9%<br>**Mean maternal education:** 14.9 years<br>**Mean household income:** NR<br>**Child Care Type**: Community child care centre	ECERS Total Score	CBI-BP NR<br>PPVT-R NR<br>WJ-ACH-LWI NR<br>WJ-ACH-AP NR	**Statistics Extracted:** Pearson's Correlation<br>**Covariates:** NA
	**Publication:** Journal (ADS)<br>**Design:** Longitudinal<br>**Data set:** North Carolina Head Start Partnership<br>**Country:** United States<br>**Sample size:** class 37; child 253<br>**% Female:** 49<br>**Mean age**: 58.8<br>**Ethnicity:** C10%, B85%, A2%, H2%, M1%<br>**Mean maternal education:** 12.4 years<br>**Mean household income:** NR<br>**Child Care Type**: Head Start	ECERS Total Score 4.2 (0.5)	ASBI-Disrupt 16.6 (7.9)<br>K-ABC 86.51 (10.14)	**Statistics Extracted:** Pearson's Correlation<br>**Covariates:** NA
	**Publication:** Journal (ADS)<br>**Design:** Longitudinal<br>**Data set:** North Carolina Public NCPPE<br>**Country:** United States<br>**Sample size:** class 63; child 263<br>**% Female:** 48<br>**Mean age:** 55.2<br>**Ethnicity:** C28%, B63%, A4%, M9%<br>**Mean maternal education:** NR<br>**Mean household income:** NR<br>**Child Care Type**: Preschool program	ECERS Total Score NR	ASBI-Disrupt NR<br>PPVT-R NR<br>WJ-ACH-LWI NR<br>WJ-ACH-AP NR	**Statistics Extracted:** Pearson's Correlation<br>**Covariates:** NA

The Relationship between the Early Childhood Environment Rating Scale and its Revised Form and Child Outcomes: a Systematic Review and Meta-Analysis

Description of Studies Meeting Inclusion Criteria[a]

Study[b]	Characteristics	Quality Measures M(SD)[c]	Outcome Measures M(SD)[d]	Covariates
Burchinal, Roberts 2000[16,T]	**Publication:** Journal (CD)<br>**Design:** Longitudinal<br>**Data set:** OMS<br>**Country:** United States<br>**Sample size:** class 22; child 51<br>**% Female:** NR<br>**Mean age:** 36<br>**Ethnicity:** B100%<br>**Mean maternal education:** 12.5 years<br>**Mean household income:** NR<br>**Child Care Type**: Centre	ECERS Total Score ("Parents and Staff" subscale scale note included) 4.0 (0.8)	Bayley-R-MDI 95.74 (10.15)<br>SICD-RCA 33.4 (4.63)<br>SICD-ECA 35.56 (4.53)	**Statistics Extracted:** Pearson's Correlation<br>**Covariates:** NA
Burchinal 2006[17, m, T]	**Publication:** Journal (PS&P)<br>**Design:** Longitudinal<br>**Data set:** OMS<br>**Country:** United States<br>**Sample size:** class NR; child 72<br>**% Female:** 55<br>**Mean age:** NR<br>**Ethnicity:** B100%<br>**Mean maternal education:** 12.5 years<br>**Mean household income:** NR<br>**Child Care Type**: Community centre	ECERS Total Score 3.46 (0.87)	Language Skills NR<br>SSRS-Preschool 101 (10.7)	**Statistics Extracted:** Pearson's Correlation<br>**Covariates:** NA
Burchinal 2008[18,A]	**Publication:** Journal (ADS)<br>**Design:** Cross-Sectional<br>**Country:** United States<br>**Sample size:** class 227; child, range by analysis 642-743<br>**% Female:** 49.52<br>**Mean age:** 54.24<br>**Ethnicity:** C42%, B21%, A4%, H23%, M9%<br>**Mean maternal education:** 12.42 years<br>**Mean household income:** $30,000<br>**Child Care Type**: Pre-K program	ECERS-R Teaching & Interactions 4.46 (1.12)	Acad. Rat. Scale 2.13 (0.82)<br>OWLS-Oral Exp. 91.46 (12.16)<br>PPVT-III 93.15 (13.71)<br>TCRS-SC 3.44 (0.74)<br>TCRS-PB 1.5 (0.51)<br>WJ-III-AP 97.6 (13.03)	**Statistics Extracted:** Pearson's Correlation<br>**Covariates:** NA
Burchinal 2011[19]<br>Sample A:<br>CQO[m, D]<br>Sample B:<br>NCEDL[m, a, A]<br>Sample C:<br>FACES 1997[m, J]<br>Sample D:<br>FACES 2000[m, K]	**Publication:** Book Chapter<br>**Design:** Cross-Sectional<br>**Data set:** CQO<br>**Country:** United States<br>**Sample size:** class 50+; child 140<br>**% Female:** 49<br>**Mean age:** 51.1<br>**Ethnicity:** C46%, B32.4%, H13.7%, O7.9%<br>**Mean maternal education:** 13 years<br>**Mean household income:** NR<br>**Child Care Type**: Centre	ECERS Interactions (author created) 4.62 (1.24)<br>ECERS Language Reasoning 4.38 (1.4)<br>ECERS Total Score 4.2 (0.9)	CBI-TO 3.3 (1.0)<br>CBI-BP 2.7 (1.0)<br>PPVT-R 83.6 (17.1)<br>WJ-LWI 94.7 (10.9)<br>WJ-AP 96.6 (12.8)	**Statistics Extracted:** Partial Correlation, B, SE<br>**Covariates:**gender, ethnicity, maternal education, site

The Relationship between the Early Childhood Environment Rating Scale and its Revised Form and Child Outcomes: a Systematic Review and Meta-Analysis

Description of Studies Meeting Inclusion Criteria[a]

Study[b]	Characteristics	Quality Measures M(SD)[c]	Outcome Measures M(SD)[d]	Covariates
	**Publication:** Book Chapter<br>**Design:** Longitudinal<br>**Data set:** NCEDL (Multi-State & SWEEP)<br>**Country:** United States<br>**Sample size:** class 50+; child 1465<br>**% Female:** 51<br>**Mean age:** 60.6<br>**Ethnicity:** C28.6%, B20.7%, H36%, O14.7%<br>**Mean maternal education:** 11.8 years<br>**Mean household income:** NR<br>**Child Care Type**: Pre-K program	ECERS-R Interactions 4.7 (1.5)<br>ECERS-R Language Reasoning 4.5 (1.1)<br>ECERS-R Total Score 3.8 (0.8)	PPVT-R 92 (13.4)<br>TCRS-SS 3.6 (0.8)<br>TCRS-PB 1.5 (0.6)<br>WJ-III-LWI 99.5 (13)<br>WJ-III-AP 96 (12.2)	**Statistics Extracted:** Partial Correlation, B, SE<br>**Covariates:**gender, ethnicity, maternal education, site
	**Publication:** Book Chapter<br>**Design:** Longitudinal<br>**Data set:** FACES 1997<br>**Country:** United States<br>**Sample size:** class 50+; child 1493<br>**% Female:** 49<br>**Mean age:** 55.4<br>**Ethnicity:** C29%, B21%, H36%, O14%<br>**Mean maternal education:** 11.7 years<br>**Mean household income:** NR<br>**Child Care Type**: Head Start	ECERS Interactions 5.0 (0.7)<br>ECERS Language Reasoning 5.0 (1.0)<br>ECERS-R Total Score 5.1 (0.6)	BPI-SS 16.4 (4.5)<br>BPI-BP 4.9 (4.5)<br>PPVT-R 84.1 (15.1)<br>WJ-LWI 90.2 (10.1)<br>WJ-AP 84.9 (17.7)	**Statistics Extracted:** Partial Correlation, B, SE<br>**Covariates:**gender, ethnicity, maternal education, site
	**Publication:** Book Chapter<br>**Design:** Longitudinal<br>**Data set:** FACES 2000<br>**Country:** United States<br>**Sample size:** class 50+; child 1739<br>**% Female:** 50<br>**Mean age:** 54<br>**Ethnicity:** C24%, B38%, H29%, O10%<br>**Mean maternal education:** 11.9 years<br>**Mean household income:** NR<br>**Child Care Type**: Head Start	ECERS Interactions 5.6 (1.6)<br>ECERS Language Reasoning 5.0 (1.3)<br>ECERS-R Total Score 4.9 (1.0)	BPI-SS 16.5 (4.6)<br>BPI-BP 5.5 (5.0)<br>PPVT-R 84.6 (16.7)<br>WJ-LWI 92.5 (10.3)<br>WJ-AP 86.6 (17.5)	**Statistics Extracted:** Partial Correlation, B, SE<br>**Covariates:**gender, ethnicity, maternal education, site
Chang 2007[20]<br>Whole Sample: NCEDL (Multi &SWEEP)[m, A]<br>Sample A: SWEEP (Spanish-Spanish Testing)[A]<br>Sample B: SWEEP (Spanish children)[A]	**Publication:** Journal (EED)<br>**Design:** Longitudinal<br>**Data set:** NCEDL (SWEEP &Mult)<br>**Country:** United States<br>**Sample size A:** class 161; child 330<br>**Sample size B:** class 161; child 134<br>**Sample size C:** class 161; child 213<br>**% Female:** 52.17<br>**Mean age:** 55.32<br>**Ethnicity:** H100%<br>**Mean maternal education:** NR<br>**Mean household income:** NR	ECERS-R Total Score (Toileting indicator and the Parents and Staff subscale not included) 3.74 (0.76)	PPVT-III 42.12 (11.76)<br>Pre-LAS 17.49 (12.45)<br>TVIP 22.4 (11.74)	**Statistics Extracted:** B, SE, Beta<br>**Covariates:** child/family level - ethnicity, income, maternal education; classroom level – teacher education, proportion Latino peers, teacher-child closeness, teacher-child language Interactions

Description of Studies Meeting Inclusion Criteria[a]

Study[b]	Characteristics	Quality Measures M(SD)[c]	Outcome Measures M(SD)[d]	Covariates
	**Child Care Type**: Prekindergarten program			
Chin-Quee 1994[21]<br>Sample A:<br>Primary 1, 2<br>Sample B:<br>Primary 3, 4	**Publication:** Journal (ED&P)<br>**Design:** Longitudinal<br>**Country:** Bermuda<br>**Sample size A:** class NR; child, range by analysis 74-96<br>**Sample size B:** class NR, child, range by analysis 74-97<br>**% Female:** 51.18<br>**Mean age:** 19.2<br>**Ethnicity:** C13%, B87%<br>**Mean maternal education:** 12.2 years<br>**Mean household income:** NR<br>**Child Care Type**: Centre	ECERS Total Score ("Parents and Staff" subscale scale note included) 3.3 (0.94)	Sample A & B<br>Academic Achievement NR<br>Social Competence-Primary NR	**Statistics Extracted:** Pearson's Correlation, Beta<br>**Covariates:** age began care, maternal education, IQ (PPVT), parental values conformity and social skills), duration in care
Clawson 2008[22]	**Publication:** Journal (TECSE)<br>**Design:** Cross-Sectional<br>**Country:** United States<br>**Sample size:** class 11; child 60<br>**% Female:** 60<br>**Mean age:** 56.52<br>**Ethnicity:** C81.7%, B5%, A1.7%, H8.3%, M3.3%<br>**Mean maternal education:** NR<br>**Mean household income:** NR<br>**Child Care Type**: Head Start	ECERS-R Total Score 5.1 (0.94)	CBCL-Parent 47.24 (10.7)<br>CBCL-Teacher 37.69 (8.36)	**Statistics Extracted:** Pearson's Correlation<br>**Covariates:** NA
Dang 2011[23]<br><br>Sample A:<br>ECLS-B 2005-2006[N]<br><br>Sample B;<br>NCEDL[A]<br><br>Sample C:<br>EHS 2001-2003[H]	**Publication:** Report<br>**Design:** Longitudinal<br>**Data set:** ECLS-B 2005-2006<br>**Country:** United States<br>**Sample size:** class 1429; child 1429<br>**% Female:** NR<br>**Mean age:** 4.40<br>**Ethnicity:** C56%, B15%, A3%, H22%, O4%<br>**Mean maternal education:** NR<br>**Mean household income:** NR<br>**Child Care Type:** Head Start	ECERS-R Total Score ("Parents and Staff" subscale scale not included) 4.53 (1.07)	ECLS-B-Literary 8.73 (1.95)<br>ECLS-B-Math 23.48 (7.05)	**Statistics Extracted:** B, SE<br>**Covariates:**pretest, gender, child's age, ethnicity, maternal education, age at baseline assessment, exclusive maternal care (a) age 9 mos., (b) age 24 mos., center hours (a) age 9 mos. (b) 24 mos., (c) 48 mos., maternal sensitivity age 9 mos. (NCATS), low birth weight, very low birth weight, 7 interaction terms, CLASS Instructional Climate
	**Publication:** Report<br>**Design:** Longitudinal<br>**Data set:** NCEDL (Multi-State & SWEEP)<br>**Country:** United States<br>**Sample size:** class 721; child 2982<br>**% Female:** NR<br>**Mean age:** 55.56<br>**Ethnicity:** C41%, B18%, H22%, O14%<br>**Mean maternal education:** NR	ECERS-R Total Score ("Parents and Staff" subscale scale not included) 3.84 (0.82)	PPVT-III 52.25 (18.2)<br>WJ-III-AP 99.11 (12.85)	**Statistics Extracted:** B, SE<br>**Covariates:**pretest, gender, child's age, ethnicity, maternal education, age at baseline assessment, exclusive maternal care (a) age 9 mos., (b) age 24 mos., center hours (a) age 9 mos. (b) 24 mos., (c) 48 mos., maternal sensitivity age 9 mos. (NCATS), low birth weight, very low birth weight, 7

The Relationship between the Early Childhood Environment Rating Scale and its Revised Form and Child Outcomes: a Systematic Review and Meta-Analysis

Description of Studies Meeting Inclusion Criteria[a]

**Study[b]**	**Characteristics**	**Quality Measures M(SD)[c]**	**Outcome Measures M(SD)[d]**	**Covariates**
	**Mean household income:** NR<br>**Child Care Type:** Pre-kindergarten programs			interaction terms, CLASS Instructional Climate
	**Publication:** Report<br>**Design:** Longitudinal<br>**Data set:** EHS 2001-2003<br>**Country:** United States<br>**Sample size:** class 241; child 241<br>**% Female:** NR<br>**Mean age:** 37.10<br>**Ethnicity:** C29%, B38%, H26%, O6%<br>**Mean maternal education:** NR<br>**Mean household income:** NR<br>**Child Care Type:** Preschool center-based care	ECERS-R Total Score ("Parents and Staff" subscale scale not included) 4.86 (1.25)	PPVT-III 90.21 (14.8)<br>WJ-III-AP 88.94 (17.99)	**Statistics Extracted:** B, SE<br>**Covariates:**pretest, gender, child's age, ethnicity, maternal education, age at baseline assessment, exclusive maternal care (a) age 9 mos., (b) age 24 mos., center hours (a) age 9 mos. (b) 24 mos., (c) 48 mos., maternal sensitivity age 9 mos. (NCATS), low birth weight, very low birth weight, 7 interaction terms, CLASS Instructional Climate
Dickinson 2001[24,m]	**Publication:** Book Chapter<br>**Design:** Longitudinal<br>**Data set:** Home-School Study<br>**Country:** United States<br>**Sample size:** class 61; child 75<br>**% Female:** 51<br>**Mean age:** NR<br>**Ethnicity:** C63.5%, B21.6%, H8.1%, M6.8%<br>**Mean maternal education:** NR<br>**Mean household income:** NR<br>**Child Care Type:** Center-based preschool	ECERS Language NR	CAP-Emergent Literacy NR<br>PPVT-R 93.86 (15.49)<br>SHELL-K-Narrative Productions 5.43 (3.07)<br>SHELL-K-Formal Definitions 1.96 (3.02)	**Statistics Extracted:** Pearson's Correlation<br>**Covariates:** NA
Dotterer 2012[25, m, A]	**Publication:** Journal (ECD&C)<br>**Design:** Longitudinal<br>**Data set:** NCEDL & SWEEP<br>**Country:** United States<br>**Sample size:** class 716; child 3548<br>**% Female:** 51.17<br>**Mean age:** 58<br>**Ethnicity:** C41%, B18%, H27%, O14%<br>**Mean maternal education:** 12.62<br>**Mean household income:** $36,041<br>**Child Care Type:** Pre-kindergarten programs	ECERS-R Teaching & Interactions<br>ECERS-R Provisions for Learning 3.76 (0.96)	Acad. Rat. Scale 92.22 (0.93)<br>Naming Letters 6.2 (3.65)<br>Naming Numbers 11.71 (9.33)<br>OWLS-Oral Exp.90.61 (12.24)<br>PPVT-III 92.22 (13.32)<br>WJ-III-R 2.76 (3.43)<br>WJ-III-AP 96.11 (12.26)	**Statistics Extracted:** B, SE<br>**Covariates:** child/family level - gender, ethnicity, maternal education; classroom level – hours per day, % Caucasian, poverty, program, poverty x program, teacher education, staff-child ratio, CLASS - Emotional Climate, CLASS – Instructional Climate
Dunn 1993[26, m, S]	**Publication:** Journal (ECRQ)<br>**Design:** Longitudinal<br>**Data set:** NCEDL<br>**Country:** United States<br>**Sample size:** class 30; child 60<br>**% Female:** 51<br>**Mean age:** 51.85<br>**Ethnicity:** B60%	ECERS Total Score 4.36 (NR)	CBI-Intellectual 53.88 (20.24)<br>CBI-Preschool 33.87 (15.67)<br>CBQ 13.78 (8.96)<br>PSI-R 44.8 (9.2)	**Statistics Extracted:** B, Pearson's Correlation, Partial Correlation<br>**Covariates:** SES, maternal education, income, experience-centre, degree

The Relationship between the Early Childhood Environment Rating Scale and its Revised Form and Child Outcomes: a Systematic Review and Meta-Analysis

Description of Studies Meeting Inclusion Criteria[a]

Study[b]	Characteristics	Quality Measures M(SD)[c]	Outcome Measures M(SD)[d]	Covariates
	**Mean maternal education:** 13.4 years<br>**Mean household income:** Between $29,000 and $34,000<br>**Child Care Type:** Day care centers			
Dunn 1994[27,27,S]	**Publication:** Journal (JRCE)<br>**Design:** Longitudinal<br>**Country:** United States<br>**Sample size:** class 30; child 60<br>**% Female:** 57<br>**Mean age:** 51.85<br>**Ethnicity:** C90%, B10%<br>**Mean maternal education:** 13.4 years<br>**Mean household income:** Between $29,000 and $34,999<br>**Child Care Type:** Community based day care centers	ECERS Total Score 36.66 (10.1)<br>ECERS Developmentally Appropriate Activities NR	CBI-Verbal 33.47 (7.29)<br>PSI 45.1 (8.98)	**Statistics Extracted:** Pearson's Correlation, Beta<br>**Covariates:** age, SES
Early 2006[28,A]	**Publication:** Journal (ECRQ)<br>**Design:** Longitudinal<br>**Data set:** NCEDL<br>**Country:** United States<br>**Sample size:** class 237; child, range by analysis 804-807<br>**% Female:** 51<br>**Mean age:** 54.7<br>**Ethnicity:** C41%, B24%, A2%, H25%, M8%<br>**Mean maternal education:** NR<br>**Mean household income:** NR<br>**Child Care Type:** Pre-Kindergarten program	ECERS-R Provision for Learning 3.79 (0.96)<br>ECERS-R Teaching & Interactions 4.43 (1.29)	Identifying Colors 9.29 (1.73)<br>Identifying Letters 12.26 (9.5)<br>Identifying Numbers 6.26 (3.67)<br>OWLS-Oral Exp. 94.79 (12.29)<br>PPVT-III 95.69 (13.58)<br>WJ-III-AP 98.56 (11.86)<br>WJ-III-SA 2.95 (3.54)	**Statistics Extracted:** Pearson's Correlation<br>**Covariates:** none
Epstein, 1993[29]	**Publication**: Book<br>**Design**: Longitudinal<br>**Dataset:** High/Scope<br>**Country**: NR<br>**Sample size:** class 26, child 200 (analyses at program level n=26)<br>**% Female**: 53.5% female<br>**Ethnicity:** NR<br>**Mean maternal education**: NR<br>**Mean household income**: NR<br>**Child Care Type:** Head Start	ECERS Total Score NR<br>ECERS Creativity NR<br>ECERS Furnishings NR<br>ECERS Language NR<br>ECERS Motor NR<br>ECERS Personal care NR<br>ECERS Social Development NR	COR-Total NR<br>COR-Logic/Math NR<br>COR-Representation NR<br>COR-Language NR<br>COR-Initiative NR<br>COR-Social NR<br>COR-Music NR<br>DIAL-R-Total NR<br>DIAL-R-Math NR<br>DIAL-R-Concepts NR<br>DIAL-R-Language NR	**Statistics Extracted:** Pearson's Correlation<br>**Covariates:** none
Fiorentino 2004[30]	**Publication:** Journal (CJBS)<br>**Design:** Cross-Sectional<br>**Country:** Canada<br>**Sample size:** class NR; child 25<br>**% Female:** 48<br>**Mean age:** 57.6	ECERS-R Total Score 4.17 (0.71)	EDI 37.46 (6.69)<br>MSSB 86.62 (62.3)	**Statistics Extracted:** F-Ratio<br>**Covariates:**none

The Relationship between the Early Childhood Environment Rating Scale and its Revised Form and Child Outcomes: a Systematic Review and Meta-Analysis

Description of Studies Meeting Inclusion Criteria[a]				
**Study**[b]	**Characteristics**	**Quality Measures M(SD)**[c]	**Outcome Measures M(SD)**[d]	**Covariates**
	**Ethnicity:** C84%, B12%, A4%<br>**Mean maternal education:** NR<br>**Mean household income:** NR<br>**Child Care Type:** Day care centers			
Goelman 1988[31]	**Publication:** Journal (ECD&C)<br>**Design:** Cross-Sectional<br>**Data set:** Canadian Victoria Day Care Research Project<br>**Country:** Canada<br>**Sample size:** class NR; child 105<br>**% Female:** 45. 28<br>**Mean age:** 54<br>**Ethnicity:** NR<br>**Mean maternal education:** NR<br>**Mean household income:** NR<br>**Child Care Type:** Day care centers	ECERS Total Score 171 (22.2)	EOWPVT NR<br>PPVT NR	**Statistics Extracted:** Pearson's Correlation<br>**Covariates:**none
Gordon 2013[32,N]	**Publication**: Journal (DP)<br>**Design**: Cross-Sectional<br>**Dataset:** ECLS-B<br>**Country**: United States<br>**Sample size:** class NR; child, range by analyses 1,100-1115 (weighted n=10,700)<br>**% Female:** 51<br>**Mean age:** NR<br>**Ethnicity:** non-Hispanic White 54%, Hispanic 26%, non-Hispanic African American 14%, Asian/Pacific Islander 3%, other 4%<br>**Mean maternal education**: NR<br>**Mean household income**: NR<br>**Child Care Type:** Head Start	ECERS-R: Total Score (Parents and Staff subscale not included)	ECLS-B Attention and Concentration 0.78 (0.86)<br>ECLS-B Emotional and Behavioral Regulation 1.01 (0.61)<br>ECLS-B Math -0.35 (0.78)<br>ECLS-B Prosocial 1.49 (0.98)	**Statistics:** Beta<br>**Covariates:** child level - pretest score, ethnicity, gender, low birth weight, breast fed, well-child checkup, received WIC, Bayley score (mental, motor, Behavior), temperament at 2 yrs. health at 2 yrs., BMI at 2 yrs., child (a) in excellent health, (b) not overweight, (c) no respiratory illness, (d) no gastrointestinal illness, (e) no ear infections, (f) no injury; family level – mother's (a) age, (b) marital status, (c) employment status, no English at home or mother not born in US, child less than 6 yrs. in home, child 6-18 yrs. in home, SES, food stamps, TANF: classroom level – CIS, group size, ratios, teacher education, ECE credentials, interest areas, child-centered activities, math activities, language activities; center level – type of center (HS, public, private, church, profit, non-profit), size and license status, accredited, accepts children with subsidies; community level – poverty rate, unbanicity, region

The Relationship between the Early Childhood Environment Rating Scale and its Revised Form and Child Outcomes: a Systematic Review and Meta-Analysis

Description of Studies Meeting Inclusion Criteria[a]

Study[b]	Characteristics	Quality Measures M(SD)[c]	Outcome Measures M(SD)[d]	Covariates
Henry, Henderson 2003[33,F]	**Publication:** Report<br>**Design:** Longitudinal<br>**Data set:** ECS 2001-2002<br>**Country:** United States<br>**Sample size:** class NR; child 466<br>**% Female:** 47<br>**Mean age:** 54<br>**Ethnicity:** C49%, B41%, O11%<br>**Mean maternal education:** NR<br>**Mean household income:** Between $40,000 and $50,000<br>**Child Care Type:** Preschool programs	ECERS-R Total Score 4.3 (0.93)	PPVT 97.1 (NR)<br>Skill Mastery NR<br>Story and Print Concepts 7.1 (NR)<br>WJ-III-LWI 105.6 (NR)<br>WJ-III-AP 99.9 (NR)	**Statistics Extracted:** B<br>**Covariates:**sex, race, age, maternal education, lives with both parents since birth, parent participation in preschool, family receives any means tested benefits, age of entry at preschool
Henry 2005[34, F]	**Publication:** Report<br>**Design:** Longitudinal<br>**Data set:** GECS<br>**Country:** United States<br>**Sample size:** class NR; child 630<br>**% Female:** 48.3<br>**Mean age:** 54<br>**Ethnicity:** C57.7%, Black =33%, H3.6%, O4.9%<br>**Mean maternal education:** NR<br>**Mean household income:** NR<br>**Child Care Type:** Head Start	ECERS-R Total Score NR	PPVT-III 106 (12.3)<br>Story & Print 7.1 (2.6)<br>WJ-III-AP 100.1 (13.3)	**Statistics:** B<br>**Covariates:**child/family level – pretest score, gender, ethnicity, income, subsidy, lived continuously with both parents, maternal education; classroom level – ability, % male, ethnicity, time spent on discipline, program type, group size, teacher's experience, teacher education (Has a BA)
Herrera 2005[35, m]	**Publication:** Journal (IJEYE)<br>**Design:** Cross-Sectional<br>**Data set:** Consists of 3 separate projects<br>**Country:** Chile<br>**Sample size:** class 120; child, range by analysis 440-466<br>**% Female:** 50<br>**Mean age:** NR<br>**Ethnicity:** NR<br>**Mean maternal education:** NR<br>**Mean household income:** NR<br>**Child Care Type:** Early childhood programs	ECERS Total Score 3.8 (0.86)	Adapted SCS NR<br>TEVI NR<br>Vineland NR	**Statistics Extracted:** Pearson's Correlation<br>**Covariates:**none
Hestenes 2015[36, m]	**Publication**: Journal (ECRQ)<br>**Design**: Cross-sectional<br>**Country**: United States<br>**Sample size:** class 97; child 422<br>**% Female:** 52<br>**Mean age:** 48.5 mo.<br>**Ethnicity:** B29%, C58%, H5%<br>**Mean maternal education**: NR<br>**Mean household income**: NR	ECERS-R 4.67 (0.72)	Learning Self-Efficacy NR<br>SSIS - Social Skills NR<br>SSIS - Externalizing Problems NR<br>SSIS - Internalizing Problems NR	**Statistics:** B, SE<br>**Covariates:** age, gender, hours in care

Description of Studies Meeting Inclusion Criteria[a]

Study[b]	Characteristics	Quality Measures M(SD)[c]	Outcome Measures M(SD)[d]	Covariates
	**Child Care Type:** Child care centers			
Hindman 2010[37, J]	**Publication:** Journal (ECRQ)<br>**Design:** Longitudinal<br>**Data set:** FACES 1997<br>**Country:** United States<br>**Sample size:** class NR; child 945<br>**% Female:** 44.5<br>**Mean age:** 51.94<br>**Ethnicity:** C32%, B25%, A2%, H33%, M7%, AI=2%<br>**Mean maternal education:** 3.08 years<br>**Mean household income:** NR<br>**Child Care Type:** Head Start	ECERS-R Total Score ("Parents and Staff" subscale scale note included) 4.95 (0.65)	WJ/WM-D 449.83 (19.67)<br>WJ/WM-AP 461.42 (16.66)	**Statistics Extracted:** B<br>**Covariates:**child/family level language skills, social skills, ethnicity, gender, age, disability diagnosis, parent involvement, maternal education, mastery, poverty/ public assistance status; classroom level - teacher background, class size, affective quality, structural features of the center, teacher experience, teacher education
Howes 1995[38]<br><br>Sample A: African[m, O]<br><br>Sample B: European[m, O]	**Publication:** Journal (JADP)<br>**Design:** Cross-Sectional<br>**Data set:** NCCSS<br>**Country:** United States<br>**Sample size A:** class NR; child 39<br>**Sample size B:** class NR; child 54<br>**% Female:** NR<br>**Mean age:** NR<br>**Ethnicity:** C58%, B42%<br>**Mean maternal education:** NR<br>**Mean household income:** NR<br>**Child Care Type:** Pre-kindergarten programs	ECERS Total Score NR	PPVT 94.15 (NR)	**Statistics Extracted:** Beta<br>**Covariates:**social class, maternal working conditions, stress, maternal working conditions (demands)
Howes 2008[39,A]	**Publication:** Journal (ECRQ)<br>**Design:** Longitudinal<br>**Data set:** NCEDL & SWEEP<br>**Country:** United States<br>**Sample size:** class 70; child, range by analysis 1787-2044<br>**% Female:** 51<br>**Mean age:** NR<br>**Ethnicity:** C42%, O58%<br>**Mean maternal education:** 12.8 years<br>**Mean household income:** NR<br>**Child Care Type:** Child care centers	ECERS-R Provision for Learning NR<br>ECERS-R Teaching & Interactions NR	Identifying Letters NR<br>Language/Literacy NR<br>OWLS-Oral Exp. NR<br>PPVT-R NR<br>WJ-III-AP NR<br>SSRS-SS NR<br>SSRS-BP NR	**Statistics Extracted:** Pearson's Correlation, B, SE<br>**Covariates:**child/family level - state, gender, child age at fall assessment, ethnicity, maternal education, poverty, number of people in the household; classroom level - teacher education (BA), ratios, in/out school, full/part-day, T-C relationship, CLASS Emotional Climate, CLASS Instructional Climate, ECERS-R Provisions for Learning for learning
Jackson 2006[40]	**Publication:** Journal (ECRQ)<br>**Design:** Longitudinal<br>**Country:** United States<br>**Sample size:** class NR; child 143<br>**% Female:** 49<br>**Mean age:** 62.8 (3 yrs) and 37.2 (4 yrs)	ECERS-R Total Score NR<br>ECERS-R Language Reasoning	TROLL NR<br>TERA-3 NR<br>WMLS NR	**Statistics Extracted:** Gamma Coefficient<br>**Covariates:**pretest score, 3 subscales from the ELLCO

Description of Studies Meeting Inclusion Criteria[a]				
**Study[b]**	**Characteristics**	**Quality Measures M(SD)[c]**	**Outcome Measures M(SD)[d]**	**Covariates**
	**Ethnicity:** C35%, B22%, A4%, H22%, M3%, O14%<br>**Mean maternal education:** NR<br>**Mean household income:** NR<br>**Child Care Type:** Head Start, child care centers, federal Even Start family literacy programs, pre-kindergarten programs			
Jeon 2010[41,m]	**Publication:** Journal (EE&D)<br>**Design:** Longitudinal<br>**Data set:** EHRSE<br>**Country:** United States<br>**Sample size:** class 106; child, range by analysis 91-102<br>**% Female:** 50<br>**Mean age:** 62.16<br>**Ethnicity:** C57%, B24%, H12%, O9%<br>**Mean maternal education:** NR<br>**Mean household income:** $22,688<br>**Child Care Type:** Pre-kindergarten programs	ECERS-R Total Score 4.76 (1.12)<br>ECERS-R Activities NR<br>ECERS-R Interactions 4.22 (1.3)<br>ECERS-R Language Reasoning NR<br>ECERS-R Program NR	PPVT-III 92.87 (14.16)<br>WJ-III-AP 89.57 (18.46)<br>STRS-Close 4.24 (0.46)	**Statistics Extracted:** Pearson's Correlation, T-Test, Beta<br>**Covariates:**site, group status (EHS program or control), minority status, disability status
Keys 2013[42]<br><br>Sample A: ECLS-B 2004-2006[N]<br><br>Sample B: NCED[m, A]<br><br>Sample C: EHS 2001-2003[m, H]	**Publication:** Journal (CD)<br>**Design:** Longitudinal<br>**Data set:** ECLS-B 2004-2006<br>**Country:** United States<br>**Sample size:** class 543; child 543<br>**% Female:** 50<br>**Mean age:** 24.59<br>**Ethnicity:** C52%, B18%, A2%, H23%, O5%<br>**Mean maternal education:** NR<br>**Mean household income:** NR<br>**Child Care Type:** Child care centers	ECERS-R Total Score ("Parents and Staff" subscale scale note included) 4.51 (1.10)	ECLS-B-Literacy 8.85 (1.92)<br>ECLS-B-Math 24.29 (7.0)<br>ECLS-B-Conduct Problems 0.16 (0.96)<br>ECLS-B-Social Skills -0.06 (01.02)	**Statistics Extracted:** Pearson's Correlation, B, SE<br>**Covariates:** child/family level - pretest scores, gender, ethnicity, school readiness skills (cognitive, attention, and externalizing Behavior problems), maternal education,child's age, low or very low birth weight, mom an immigrant, maternal sensitivity, exclusive maternal care (a) 9 (b) 24 months, hours of center care (a) 9 (b) 24 (c) 48 months, differences between age, interaction terms
	**Publication:** Journal (CD)<br>**Design:** Longitudinal<br>**Data set:** NCEDL (Multi-State & SWEEP)<br>**Country:** United States<br>**Sample size:** class 721; child 2982<br>**% Female:** 51<br>**Mean age:** 24.59<br>**Ethnicity:** C41%, B18%, H26%, O14%<br>**Mean maternal education:** NR<br>**Mean household income:** NR<br>**Child Care Type:** Head Start	ECERS-R Total Score ("Parents and Staff" subscale scale note included) 3.84 (0.81)	PPVT-III 96.3 (14.31)<br>TCRS-Prob. Behavior 1.57 (0.7)<br>TCRS-Social Skills 3.64 (0.7)<br>WJ-III-AP 99.11 (12.85)	**Statistics Extracted:** Pearson's Correlation, B, SE<br>**Covariates:** child/family level - pretest scores, gender, ethnicity, school readiness skills (cognitive, attention, and externalizing Behavior problems), maternal education,child's age at fall and spring assessments, family income at or below 150% of poverty, no. of people in household, grandma present, father present, step father present, interaction terms

Description of Studies Meeting Inclusion Criteria[a]				
Study[b]	Characteristics	Quality Measures M(SD)[c]	Outcome Measures M(SD)[d]	Covariates
	**Publication:** Journal (CD)<br>**Design:** Longitudinal<br>**Data set:** EHS 2001-2003<br>**Country:** United States<br>**Sample size:** class NR; child 676<br>**% Female:** 49<br>**Mean age:** 55.56<br>**Ethnicity:** C37%, B33%, H24%, O6%<br>**Mean maternal education:** NR<br>**Mean household income:** NR<br>**Child Care Type:** Pre-kindergarten programs	ECERS-R Total Score ("Parents and Staff" subscale scale note included) 5.25 (1.14)	CBCL-Aggressive 11.03 (6.78)<br>PPVT-III 92.44 (15.29)<br>WJ-III-AP 89.07 (20.09)	**Statistics Extracted:** Pearson's Correlation, B, SE<br>**Covariates:** child/family level - pretest scores, gender, ethnicity, school readiness skills (cognitive, attention, and externalizing Behavior problems), child's age at 24 mo. assessment, maternal education, primary language is English, poverty level, teenage mom, mother's partner lives in home, H.O.M.E. score, program group, interaction terms
Kontos 1991[43, m]	**Publication:** Journal (ECRQ)<br>**Design:** Cross-Sectional<br>**Country:** United States<br>**Sample size:** class NR; child 100<br>**% Female:** 47<br>**Mean age:** 52.8<br>**Ethnicity:** NR<br>**Mean maternal education:** 13.07 years<br>**Mean household income:** NR<br>**Child Care Type:** Child care centers	ECERS Total Score 139.62 (21.6)	ALI 60.29 (11.41)<br>CBI-Intellectual 52.83 (18.34)<br>CBI-Sociability 31.89 (14.48)<br>PBQ 15.91 (9.6)<br>Slosson Intelligence 112.47 (16.84)<br>TELD-Language 101.72 (13.07)	**Statistics Extracted:** Pearson's Correlation, Beta<br>**Covariates:**age, maternal education, whether child was receiving subsidy, maternal values - social, age at entry into care, length of time in care
Kwan 1998[44]	**Publication:** Journal (ECD&C)<br>**Design:** Longitudinal<br>**Country:** Singapore<br>**Sample size:** class 16; child 116<br>**% Female:** NR<br>**Mean age:** NR<br>**Ethnicity:** NR<br>**Mean maternal education:** NR<br>**Mean household income:** NR<br>**Child Care Type:** Child care centers	ECERS Total Score NR<br>ECERS Adult Needs NR<br>ECERS Creative Activities NR<br>ECERS Fine and Gross Motor NR<br>ECERS Furnishings and Display<br>ECERS Language Reasoning NR<br>ECERS Personal Care and Routine<br>ECERS Social Development NR	BAS-Verbal Fluency NR<br>BAS-Word Reading NR	**Statistics Extracted:** Effect Size<br>**Covariates:**center, pretest score, child characteristics (NR), mother's education, parental values
Le 2015[45, Z]	**Publication**: Journal (ECRQ)<br>**Design:** Longitudinal<br>**Dataset:** Colorado's QRIS<br>**Country**: United States<br>**Sample size:** centers 49; child, range by analyses 292-300<br>**% Female:** NR<br>**Mean age:** 55.46 mo.<br>**Ethnicity: NR**<br>**2014 Mean maternal education**: NR<br>**Mean household income**: NR<br>**Child Care Type:** Community-based child care centers	ECERS-R Total Score ("Parents and Staff" subscale scale note included) 5.43 (0.79)	PPVT 96.67 (14.21)<br>Social Competence NR<br>WJ – AP 109.90 (13.32)<br>WJ – LWI 103.77 (15.81)<br>WJ – PC 98.73 (13.13)	**Statistics:** B, SE<br>**Covariates:** child level - pretest score, ethnicity, age at assessment, gender, hours per week of care, length of time in center, number of months between assessments; family level - met state median income, parent's education (Has BA); classroom level - non-profit status, HS, NAEYC accreditation status, family partnership points, classroom teaching experience, directors' admin experience, number of ECE credits (teacher and director), has

The Relationship between the Early Childhood Environment Rating Scale and its Revised Form and Child Outcomes: a Systematic Review and Meta-Analysis

Description of Studies Meeting Inclusion Criteria[a]

**Study**[b]	**Characteristics**	**Quality Measures M(SD)**[c]	**Outcome Measures M(SD)**[d]	**Covariates**
				a BA (teacher and director)
Lyon 1995[46]	**Publication:** Report<br>**Design:** Cross-Sectional<br>**Data set:** Atlantic Day Care Study<br>**Country:** Canada<br>**Sample size:** class NR; child, range by analysis 256-551<br>**% Female:** 50<br>**Mean age:** 47.53<br>**Ethnicity:** NR<br>**Mean maternal education:** NR<br>**Mean household income:** NR<br>**Child Care Type:** Child care centers	ECERS Total Score 4.52 (0.7)<br>ECERS Developmentally Appropriate Activities 4.04 (0.93)<br>ECERS Preschool Appropriate Caregiving 4.79 (0.74)	ALI 57.1 (12.24)<br>PPVT 97.68 (14.94)<br>Entwistle Scale 73.06 (14.72)<br>PSPCSAYC-Peer Acceptance 3 (0.64)<br>PSPCSAYC-Maternal Acceptance 3.11 (0.6)<br>PSPCSAYC-Physical Competence 3.23 (0.5)<br>PSPCSAYC-Cognitive Competence 3.58 (0.47)	**Statistics Extracted:** F-Ratio<br>**Covariates:**none
Mashburn 2008[47, m, F]	**Publication:** Journal (ADS)<br>**Design:** Longitudinal<br>**Data set:** GECS<br>**Country:** United States<br>**Sample size:** class 124; child 540<br>**% Female:** 47<br>**Mean age:** NR<br>**Ethnicity:** C50%, O50%<br>**Mean maternal education:** NR<br>**Mean household income:** $36,389<br>**Child Care Type:** Head Start, the Georgia pre-kindergarten program, private preschools	ECERS-R Total Score ("Parents and Staff" subscale scale note included) 4.35 (0.92)	Academic Skills 100.1 (11.7)<br>Language Skills 95.3 (13.3)<br>Literacy Skills 4.93 (2.55)	**Statistics Extracted:** B, SE<br>**Covariates:**pretest score, assessment interval, sex, race, family income, type of program
Mashburn, Pianta 2008[48, m, A]	**Publication:** Journal (CD)<br>**Design:** Longitudinal<br>**Data set:** NCEDL & SWEEP<br>**Country:** United States<br>**Sample size:** class 671; child, range by analysis 2307-2439<br>**% Female:** 51<br>**Mean age:** NR<br>**Ethnicity:** C46%, B21%, H27%, O15%<br>**Mean maternal education:** 12.9 years<br>**Mean household income:** NR<br>**Child Care Type:** Pre-kindergarten programs	ECERS-R Total Score ("Parents and Staff" subscale scale note included) 3.85 (0.82)	Letter Naming 13.9 (9.42)<br>OWLS-Oral Exp. 93.6 (13)<br>PPVT-III 96.3 (14.3)<br>TCRS-SS 3.66 (0.7)<br>TCRS-PB 1.49 (0.54)<br>WJ-III-SA 3.65 (4.02)<br>WJ-III-AP 99.1 (12.9)	**Statistics Extracted:** B, SE<br>**Covariates:** pretest scores, gender, ethnicity, mother's education, poverty, state
McCartney 1982[49, m, C]	**Publication:** Book Chapter<br>**Design:** Cross-Sectional<br>**Data set:** Bermuda Study<br>**Country:** Bermuda<br>**Sample size:** class NR; child, range by analysis 130-150<br>**% Female:** NR	ECERS Total Score 123.2 (35.3)	Adult Orientation 3.0 (0.6)<br>ALI 3.1 (0.7)<br>CBI 3.1 (0.9)<br>CBI-Consideration 3.0 (0.4)<br>CBI-Dependency 2.5 (0.7)<br>CBI-Sociability 2.9 (0.5)<br>CBI-Task Orientation 3.1 (0.9)	**Statistics Extracted:** Pearson's Correlation, B, SE<br>**Covariates:**age, mother's PPVT, maternal education, maternal ethnicity, age at entry, number of hours in group care, adult talk to children

Description of Studies Meeting Inclusion Criteria[a]				
Study[b]	Characteristics	Quality Measures M(SD)[c]	Outcome Measures M(SD)[d]	Covariates
	**Mean age:** 46.6<br>**Ethnicity:** C13%, B83%, O4%<br>**Mean maternal education:** 12.2 years<br>**Mean household income:** NR<br>**Child Care Type:** Day care centers		PBQ-Aggressive-Hostile 1.7 (0.5)<br>PBQ-Anxious 1.4 (0.2)<br>PBQ-Hyperactive-Distractible 2.0 (0.5)<br>PBQ-Total Maladjustment 1.7 (0.4)<br>PLAI 1.3 (0.5)<br>PPVT-R 82.8 (16.7)	
McCartney 1984[50, C]	**Publication:** Journal (DP)<br>**Design:** Cross-Sectional<br>**Data set:** Bermuda Study<br>**Country:** Bermuda<br>**Sample size:** class NR; child, range by analysis 46-131<br>**% Female:** NR<br>**Mean age:** NR<br>**Ethnicity:** C22%, B78%<br>**Mean maternal education:** 12.2 years<br>**Mean household income:** NR<br>**Child Care Type:** Day care centers	ECERS Total Score ("Parents and Staff" subscale scale note included) 123.2 (35.3)	ALI 3.1 (0.7)<br>Communication Task34.4 (12.9)<br>PPVT-R 82.8 (16.7)<br>PLAI 1.3 (0.5)	**Statistics Extracted:** Pearson's Correlation, Beta<br>**Covariates:** age of testing, parental values (social skills), parental values (conformity), age of entry, hours in center care
McWayne 2004[51]	**Publication:** Journal (DP)<br>**Design:** Cross-Sectional<br>**Country:** United States<br>**Sample size:** class 32; child 195<br>**% Female:** 47<br>**Mean age:** 62.52<br>**Ethnicity:** C12%, B81%, A2%, H5%<br>**Mean maternal education:** NR<br>**Mean household income:** NR<br>**Child Care Type:** Head Start	ECERS-R Total Score NR	Competency Profile NR<br>ESI-K NR	**Statistics Extracted:** F-Ratio, Beta<br>**Covariates:**age, gender, neighbourhood structural danger, neighbourhood social stress
Moller…Hightower 2008[52,R]	**Publication:** Journal (JEP)<br>**Design:** Longitudinal<br>**Country:** United States<br>**Sample size:** class 70; child 806<br>**% Female:** 49<br>**Mean age:** 49.8<br>**Ethnicity:** C17%, B57%, A2%, H15%, O9%<br>**Mean maternal education:** NR<br>**Mean household income:** NR<br>**Child Care Type:** Preschool classrooms	ECERS-R Total Score 6.42 (0.77)	COR-Cognitive 3.07 (0.77)<br>COR-Moto Skills 3.65 (0.8)<br>COR-Total 3.48 (0.72)<br>COR-Social 3.72 (0.8)	**Statistics Extracted:** B, SE<br>**Covariates:**pretest score, age, gender, class chronological age SD, interaction between age SD and child's age, class size
Moller…Friedman 2008b[53, R]	**Publication:** Journal (ECRQ)<br>**Design:** Longitudinal<br>**Country:** United States<br>**Sample size:** class 70; child 806<br>**% Female:** 49<br>**Mean age:** 49.8<br>**Ethnicity:** C17%, B57%, A2%, H15%, O9%	ECERS-R Total Score 6.52 (0.64)	COR-Cognitive 3.07 (0.77)<br>COR-Motor Skills 3.65 (0.8)<br>COR-Total 3.48 (0.72)<br>COR-Social 3.72 (0.8)	**Statistics Extracted:** B, SE<br>**Covariates:** pretest score, age, gender, gender composition of classroom, class gender x child's gender, group size

The Relationship between the Early Childhood Environment Rating Scale and its Revised Form and Child Outcomes: a Systematic Review and Meta-Analysis

Description of Studies Meeting Inclusion Criteria[a]				
**Study**[b]	**Characteristics**	**Quality Measures M(SD)**[c]	**Outcome Measures M(SD)**[d]	**Covariates**
	**Mean maternal education:** NR<br>**Mean household income:** NR<br>**Child Care Type:** Preschool programs			
Montes 2005[54]	**Publication:** Journal (ECRQ)<br>**Design:** Longitudinal<br>**Data set:** RECAP Study<br>**Country:** United States<br>**Sample size:** class 88; child 1551 (analyses at classroom level n=88)<br>**% Female:** 51.3<br>**Mean age:** NR<br>**Ethnicity:** Black or H78.4%<br>**Mean maternal education:** NR<br>**Mean household income:** NR<br>**Child Care Type:** Pre-kindergarten programs	ECERS-R Total Score 6.3 (NR)	TCRS-Task Orientation NR<br>TCRS-Behavior Control NR<br>TCRS-Assertiveness NR<br>TCRS-Peer Social Skills NR<br>TCRS-Multiple Risk Factors NR<br>TCRS-Percent not at Risk NR<br>TCRS-Decreased Risk Status NR<br>TCRS-Increased Risk Status NR	**Statistics Extracted:** F-Ratio, B, T-Test, Cohen's D<br>**Covariates:**gender, minority ethnicity distribution of the classroom
Peisner-Feinberg 1997[55, m, D]	**Publication:** Journal (MPQ)<br>**Design:** Cross-Sectional<br>**Data set:** CQO<br>**Country:** United States<br>**Sample size:** class 177; child 757<br>**% Female:** 48.9<br>**Mean age:** 51.6<br>**Ethnicity:** C67.9%, B15.9%, H4.6%, O11.6%<br>**Mean maternal education:** 14.22 years<br>**Mean household income:** $38,900<br>**Child Care Type:** Child care centers	ECERS Total Score ("Parents and Staff" subscale scale note included) 4.25 (1.03)	CBI-PB 3.62 (0.71)<br>PPVT-R 93.59 (18.48)<br>WJ-AP 102.38 (13.45)<br>WJ-LWI 99.65 (13.02)	**Statistics Extracted:** Pearson's Correlation<br>**Covariates:**none
Pesiner-Feinberg 1999[56, D]	**Publication:** Report<br>**Design:** Longitudinal<br>**Data set:** CQO<br>**Country:** United States<br>**Sample size:** class 149; child 418<br>**% Female:** 48.8<br>**Mean age:** 61.2<br>**Ethnicity:** C72.1%, O28%<br>**Mean maternal education:** 14.4 years<br>**Mean household income:** $48,421<br>**Child Care Type:** Pre-kindergarten programs	ECERS Total Score (Mean based on 5 items) 4.05 (1.21)	CBI-Cognitive 3.85 (0.71)<br>CBI-Problem Behavior 2.38 (0.88)<br>CBI-Sociability 4.14 (0.67)<br>PPVT-R 101.69 (17.71)<br>WJ-ACH-AP 106.14 (15.09)<br>WJ-ACH-LWI 100.38 (13.23)	**Statistics Extracted:** Pearson's Correlation, Effect Size (regression)<br>**Covariates:**maternal education, gender, ethnicity
Peisner-Feinberg 2006[57, YA,YC]	**Publication:** Report<br>**Design:** Longitudinal<br>**Data set:** NC More at Four (2003-2005)<br>**Country:** United States<br>**Sample size:** class 58; child 185<br>**% Female:** NR<br>**Mean age:** 54	ECERS-R Total Score ("Parents and Staff" subscale scale not included) 5.3 (0.7)	Color Naming 19.7 (1.3)<br>Counting Task 34.1 (9.2)<br>Naming Letters 24.8 (4)<br>PPVT-III 96.7 (13.4)<br>Social Awareness 4.9 (1.2)<br>SSRS-PB 98.7 (12.5)<br>SSRS-SS 101.7 (14.2)	**Statistics Extracted:** B, SE<br>**Covariates:**age, gender, risk status, service priority status, English proficiency, ECERS X Time

The Relationship between the Early Childhood Environment Rating Scale and its Revised Form and Child Outcomes: a Systematic Review and Meta-Analysis

Description of Studies Meeting Inclusion Criteria[a]

Study[b]	Characteristics	Quality Measures M(SD)[c]	Outcome Measures M(SD)[d]	Covariates
	**Ethnicity:** NR<br>**Mean maternal education:** NR<br>**Mean household income:** NR<br>**Child Care Type:** Head Start, community centers		Story & Print 9.2 (2.3)<br>WJ-III-AP 100.6 (11)<br>WJ-III-R 9.1 (4.3)	
Peisner-Feinberg 2007[58, YC]	**Publication:** Report<br>**Design:** Longitudinal<br>**Data set:** NC More at Four 2005-2006<br>**Country:** United States<br>**Sample size:** class 57; child, range by analysis 372-445<br>**% Female:** 49.8<br>**Mean age:** 54<br>**Ethnicity:** C32.6%, B30.1%, A1.3%, H28%, O8%<br>**Mean maternal education:** NR<br>**Mean household income:** NR<br>**Child Care Type:** Centre-based child care	ECERS-R Total Score (“Parents and Staff” subscale scale note included) 4.2 (0.7)	Color Bears 18.6 (3.2)<br>Counting Bears 18.9 (10.6)<br>Naming Letters 15.3 (9.6)<br>PPVT-III 87 (19.6)<br>Social Awareness 4.2 (1.5)<br>SSRS-PB 99.5 (13.2)<br>SSRS-Social 109.7 (14.7)<br>Story & Print Concepts 4.7 (2.6)<br>WJ-AP 94 (14.3)<br>WJ-R 3.8 (3.8)	**Statistics Extracted:** B, SE<br>**Covariates:**age, gender, children's risk, English proficiency level, attendance
Peisner-Feinberg 2008[59, m, YB, YC]	**Publication:** Report<br>**Design:** Longitudinal<br>**Data set:** NC More at Four (2003-2007)<br>**Country:** United States<br>**Sample size:** class 105; child 722<br>**% Female:** 50<br>**Mean age:** 54<br>**Ethnicity:** C34.58%, B33.57%, A1%, H22.28%<br>**Mean maternal education:** NR<br>**Mean household income:** NR<br>**Child Care Type:** Head Start, community child care centers	ECERS-R Total Score (“Parents and Staff” subscale scale note included) NR	Color Naming NR<br>Counting Task NR<br>Naming Letters NR<br>PPVT-III NR<br>Social Awareness NR<br>SSRS-PB NR<br>SSRS-SS NR<br>Story & Print NR<br>WJ-III-AP NR<br>WJ-III-R NR	**Statistics Extracted:** T-Test, df<br>**Covariates:**age at entry to MAF, dosage of MAF, gender, time elapsed between assessments, class, cohort, cumulative risk level, English proficiency level
Peisner-Feinberg 2008[60, m]	**Publication:** Report<br>**Design:** Longitudinal<br>**Data set:** NC More at Four 2007-2008<br>**Country:** United States<br>**Sample size:** class 50; child, 321<br>**% Female:** 53.9<br>**Mean age:** 55.2<br>**Ethnicity:** C29.3%, B35.8%, A1.6%, H24.9%, O8.4%<br>**Mean maternal education:** NR<br>**Mean household income:** NR<br>**Child Care Type:** Head Start, public preschool, private for profit child care, private for non-profit child care	ECERS-R Total Score (“Parents and Staff” subscale scale note included) 4.4 (1.0)	85.3 (15.2)<br>Counting Task 18 (11)<br>PPVT-4 91 (17.2)<br>Social Awareness 4.2 (1.6)<br>SSRS-PB 99.5 (13.2)<br>SSRS-SS 109.4 (14.6)<br>TOPEL-Phonological Awareness<br>TOPEL-Print Knowledge 95.8 (14.1)<br>WJ-III-AP 98.2 (12.3)<br>WJ-III-LWI 96.5 (12.3)	**Statistics Extracted:** T-Test<br>**Covariates:**pretest, gender, age at first assessment, days elapsed since previous assessment, days of attendance at More at Four

The Relationship between the Early Childhood Environment Rating Scale and its Revised Form and Child Outcomes: a Systematic Review and Meta-Analysis

Description of Studies Meeting Inclusion Criteria[a]

**Study[b]**	**Characteristics**	**Quality Measures M(SD)[c]**	**Outcome Measures M(SD)[d]**	**Covariates**
Peisner-Feinberg 2013[61, m]	**Publication:** Report<br>**Design:** Longitudinal<br>**Data set:** Georgia Pre-K 2011-2012<br>**Country:** United States<br>**Sample size:** class 99; child, range by analysis 454-469<br>**% Female:** 52.1<br>**Mean age:** 54<br>**Ethnicity:** C35%, B39%, A4%, H15%, M3%, O4%<br>**Mean maternal education:** NR<br>**Mean household income:** NR<br>**Child Care Type:** Local school systems, private settings, Head Start	ECERS-R Total Score 3.6 (0.6)	Counting 11.7 (1.0)<br>Naming Letters 8.0 (0.0)<br>Social Awareness 4.8 (1.3)<br>SSIS-Prob. Behavior 100.3 (15.8)<br>SSIS-Social Skills 100.2 (14.7)	**Statistics Extracted:** B, SE<br>**Covariates:** child/family level - gender, child's age at fall assessment, family income, English proficiency; classroom level - program type (school, private), lead teacher certified, lead teacher years of experience teaching pre-k, percent non-English-speaking children in class, CLASS (Emotional Support, Classroom Organization, and Instructional Support)
Phillips 1987[62, C]	**Publication:** Journal (DP)<br>**Design:** Cross-Sectional<br>**Data set:** Bermuda Study<br>**Country:** Bermuda<br>**Sample size:** class NR; child, range by analysis 153-156<br>**% Female:** NR<br>**Mean age:** NR<br>**Ethnicity:** C22%, B78%<br>**Mean maternal education:** NR<br>**Mean household income:** NR<br>**Child Care Type:** Child care centers	ECERS Total Score ("Parents and Staff" subscale scale note included) 123.2 (NR)	CBI-Intelligence NR<br>CBI-Considerateness NR<br>CBI-Sociability NR<br>CBI-Task Orientation NR<br>CBI-Dependence NR<br>PBQ-Aggression NR<br>PBQ-Anxiety NR<br>PBQ-Hyperactivity NR	**Statistics Extracted:** R-Squared Change<br>**Covariates:** age at testing, values conformity, values social skills, age at entry, time in group care
Pinto 2013[63,m]	**Publication**: Journal (ECRQ)<br>**Design**: Longitudinal<br>**Country**: Portugal<br>**Sample size:** class NR; 95<br>**% Female:** 51.6<br>**Mean age:** 26.33 mo.<br>**Ethnicity:** NA<br>**Mean maternal education**: 10.64<br>**Mean household income**: 1574.29 Euros/mo<br>**Child Care Type:** Preschool classrooms	ECERS-R (Portuguese Version) 3.32 (0.89)	Griffiths – Language 93.04 (12.62)<br>Story & Print Concepts 7.44 (4.30)<br>VABS - Communication 15.32 (4.21)	**Statistics:** Pearson Correlation, B, SE, Effect Size<br>**Covariates:**prior developmental level, maternal education, home environment quality at Time 1
Reid 2013[64, A]	**Publication**: Journal (EED)<br>**Design**: Longitudinal<br>**Dataset:** NCEDL (Multi-State & SWEEP)<br>**Country**: United States<br>**Sample size:** class 704; child 2,966<br>**% Female:** NR<br>**Mean age:** NR<br>**Ethnicity:** NR<br>**2014 Mean maternal education**: 12.8	ECERS-R Total Score ("Parents and Staff" subscale scale note included)<br><br>Z-scores<br>Low SES classrooms -0.20 (0.99)<br>Mid SES classrooms 0.00 (0.95)<br>High SES classrooms 0.31 (1.01)	PPVT NR<br>OWLS-Oral Exp. NR<br>WJ-III AP NR<br>TCRS-Social Competence NR	**Statistics:** Beta<br>**Covariates:**child/family level - pretest score, gender, age, SES, ethnicity, single parent, ELL status, IEP status; classroom level - SES, deviation of income, percent Caucasian, CLASS - Instructional Quality, teacher has BA, teacher has more than a BA, class size (less than 18), full-day, Head Start,

Description of Studies Meeting Inclusion Criteria[a]

Study[b]	Characteristics	Quality Measures M(SD)[c]	Outcome Measures M(SD)[d]	Covariates
	**Mean household income**: 32, 574<br>**Child Care Type:** School based			interaction terms
Sabol 2013[65, A]	**Publication**: Journal (EED)<br>**Design**: Longitudinal<br>**Dataset:** NCEDL (Multi-State & SWEEP)<br>**Country**: United States<br>**Sample size:** program 673; child 2419<br>**% Female:**<br>**Mean age:** 4.61<br>**Ethnicity:** C42%, B25%, H18%, O15%<br>**Mean maternal education**: 12.96 years<br>**Mean household income**: NR<br>**Child Care Type:** Head start, public day care centers, private day care centers	ECERS-R Total Score 3.85 (0.80)<br>ECERS -R Teaching and Interactions 4.69 (1.17)	Letter Knowledge 14.40 (9.34)<br>OWLS-Oral Exp. 93.21 (13.45)<br>PPVT-III 95.52 (14.70)<br>TCRS-Problem Behaviors 1.49 (0.55)<br>TCRS-Social Skills 3.56 (0.77)<br>WJ AP 98.88 (13.37)<br>WJ Rhyming 3.36 (3.82)	**Statistics:** Pearson's Correlation, Beta<br>**Covariates:** child/family level - pretest score, gender, ethnicity, maternal education, poverty, household size, attend pre-k prior year; classroom level -state, ethnicity, Head Start
Sabol 2014[66, m, N]	**Publication**: Journal (EED)<br>**Design**: Longitudinal<br>**Dataset:** ECLS-B<br>**Country**: United States<br>**Sample size:** class NR, child 800<br>**% Female:** 51%<br>**Mean age:** 65 months<br>**Ethnicity:** H22%, B14% A3% Other 4%<br>**Mean maternal education**: NR<br>**Mean household income**:<br>**Child Care Type:** School based programs	ECERS-R 4.51 (1.01)	ARS-Literacy 51.83 (9.21)<br>ECLS-B Math 51.41 (8.63)<br>ECLS-B Prosocial 51.12 (10.47)<br>ECLS-B Externalizing 49.69 (9.61)<br>PreLas 51.86 (7.75)	**Statistics:** Pearson's Correlation, B, SE<br>**Covariates:** child level – pretest score, gender, ethnicity, low birth weight, multiple birth, age wave 4, age at entry, hours in care wave 1-2, hours in care wave 3, 9-mo. mental ability, 9-mo. motor skills, in kindergarten wave 4, assessment interval; family level – maternal education (<BA), single mother, household size, SES, food stamps, WIC, owed in child support, subsidized housing, no car, non-English home, mother poor English, mother age at child's birth, parenting skills (a) cognitive, (b) detachment, (c) emotional supportiveness, sociodemographic risk status; classroom level – teacher BA or more, ECE degree, child-staff ratio, group size, percent ELL, percent special needs; center level – region, non-English care, HS, public, private, enrollment, ethnicity, risk X ECERS-R
Schliecker 1991[67]<br><br>Sample A:<br>Whole Sample[m]<br><br>Sample B:<br>One Parent	**Publication:** Journal (CJBS)<br>**Design:** Cross-Sectional<br>**Country:** Canada<br>**Sample size A:** class NR; child 100<br>**Sample size B:** class NR; child 63<br>**Sample size C:** class NR; child 37<br>**% Female:** 48	ECERS Total Score NR	PPVT-R 98.03 (19.63)	**Statistics Extracted:** Pearson's Correlation, T-Test, Beta<br>**Covariates:** maternal age, maternal education, maternal occupation prestige, paternal age, paternal occupational prestige

The Relationship between the Early Childhood Environment Rating Scale and its Revised Form and Child Outcomes: a Systematic Review and Meta-Analysis

Description of Studies Meeting Inclusion Criteria[a]

Study[b]	Characteristics	Quality Measures M(SD)[c]	Outcome Measures M(SD)[d]	Covariates
Sample C:<br>Two Parent	**Mean age:** NR<br>**Ethnicity:** NR<br>**Mean maternal education:** NR<br>**Mean household income:** NR<br>**Child Care Type:** Day care centers			
Seppanen 1993[68]	**Publication:** Report<br>**Design:** Longitudinal<br>**Data set:** OSECP<br>**Country:** United States<br>**Sample size:** class 55; child 673<br>**% Female:** 48<br>**Mean age:** NR<br>**Ethnicity:** C15%, B48%, H31%, O7%<br>**Mean maternal education:** NR<br>**Mean household income:** NR<br>**Child Care Type:** Pre-kindergarten programs	ECERS Total Score ("Parents and Staff" subscale scale note included) 4.7 (0.7)	CBRS NR<br>PSI NR	**Statistics Extracted:** Partial Correlation<br>**Covariates:** pretest score
Sylva et al., 2006[69, E]	**Publication:** Journal (ECRQ)<br>**Design:** Longitudinal<br>**Dataset:** EPPE<br>**Country:** United States<br>**Sample size:** class 141; child 2857<br>**% Female:** 47.9<br>**Mean age:** 40 mo.<br>**Ethnicity:** C78.6%, B6.3%, M6.5%, O8.5%<br>**Mean maternal education:** NR<br>**Mean household income:** NR<br>**Child Care Type:** Preschool centers	ECERS-R Total Score 4.34 (1.0)<br>ECERS-R Activities 3.83 (1.16)<br>ECERS-R Interactions 4.82 (1.31)<br>ECERS-R Language Reasoning 4.32 (1.33)<br>ECERS-R Parents and Staff 4.07 (1.28)<br>ECERS-R Personal Care Routines 3.81 (1.36)<br>ECERS-R Program Structure 4.7 (1.47)<br>ECERS-R Space and Furnishings 4.85 (1.04)	Antisocial/Worried 1.74 (0.66)<br>BAS II-Number Concepts 18.5 (5.66)<br>BAS II-Pattern 11.6 (7.27)<br>BAS II-Picture 22.38 (4.54)<br>Cooperation Conformity 3.92 (0.68)<br>Independence 3.54 (0.83)<br>Language 42.13 (7.68)<br>Peer Sociability 3.65 (0.71)<br>Pre-Reading 21.57 (12.67)	**Statistics Extracted:** B, SD, Effect Size<br>**Covariates:**child/family level - pretest score, age, gender, ethnicity, birth weight, language, number of siblings, SES, maternal education, poverty, frequency parent teaches (a) alphabet sounds, (b) letters/numbers, frequency child (a) paints/draws at home, (b) changed preschool, developmental problems before age 3; classroom level - percentage of mothers who have degree or higher, duration in preschool during EPPE study, duration in reception class before assessment was completed, percentage of children at centre and whose scores were 1 SD lower than mean BAS-II GCA
Weiland 2013[70,m]	**Publication**: Journal (ECRQ)<br>**Design**: Longitudinal<br>**Country**: United States<br>**Sample size:** class NR; child 414<br>**% Female:** 50.0<br>**Mean age:** 26.33 mo.<br>**Ethnicity:** C16%, B28%, H43%, A11%<br>**Mean maternal education**: NA<br>**Mean household income**: NA<br>**Child Care Type:** Public school-based pre-kindergarten programs	ECERS-R Total Score 5.54 (1.21)<br>ECERS-R Provisions for Learning 4.47 (0.50)<br>ECERS-R Teaching and Interactions 3.72 (0.55)	Backward Digit 1.44 (0.72)<br>Forward Digit 4.51 (1.25)<br>PPVT-III 94.45 (17.89)<br>Pencil Tap 11.65 (5.35)	**Statistics:** B, SE<br>**Covariates:**child family level - pretest scores, ethnicity, gender, home language, free/reduced lunch, special needs, age, attendance zone, pre-BPS experience; classroom level - teacher education (MA), group size

Description of Studies Meeting Inclusion Criteria[a]

Study[b]	Characteristics	Quality Measures M(SD)[c]	Outcome Measures M(SD)[d]	Covariates
West 2010[71, m, B]	**Publication:** Report<br>**Design:** Longitudinal<br>**Data set:** FACES 2006<br>**Country:** United States<br>**Sample size:** class 410; child, range by analysis 426-684<br>**% Female:** 49.3<br>**Mean age:** NR<br>**Ethnicity:** C24.9%, B26.8%, A1.4%, H38.9%, M5.4%, O2.5%<br>**Mean maternal education:** NR<br>**Mean household income:** NR<br>**Child Care Type:** Head Start	ECERS-R-Provision for Learning NR<br>ECERS-R-Teaching & Interactions NR	Behavior Problems 6.7 (NR)<br>ECLS-Math 19 (NR)<br>PPVT-4 95 (NR)<br>Social Skills 18 (NR)<br>WJ-III-AP 401.5 (NR)<br>WJ-III-LWI 334.5 (NR)	**Statistics Extracted:** Beta<br>**Covariates:**child/family level - child's exposure to HS (1 vs. 2 years), gender, ethnicity, language, poverty ratio, joint book reading at least 3 times per week, number of books in home, maternal education, parent depressive symptoms, Low/mid/high ability at HS entry; classroom level - mean peer abilities at HS entry on PPVT-4, variation in peer abilities at HS entry on PPVT-4, full day/half day, CLASS - Instructional Support, teacher education (Has a BA)
Whitebook 1989[72,O]	**Publication:** Report<br>**Design:** Longitudinal<br>**Data set:** NCCSS<br>**Country:** United States<br>**Sample size:** class NR; child, range by analysis 106-125<br>**% Female:** 43<br>**Mean age:** NR<br>**Ethnicity:** C52%<br>**Mean maternal education:** NR<br>**Mean household income:** NR<br>**Child Care Type:** Centre-based programs	ECERS Preschool Appropriate Caregiving 4.09 (0.94)	ALI 56.25 (12.05)<br>ESPM 64.72 (9.26)<br>PPVT-III 94 (17.67)<br>PSPCAY (Teacher) 2.94 (0.63)<br>PSPCAYC (child) 3.37 (0.4)	**Statistics Extracted:** Partial Correlation, F-Ratio<br>**Covariates:**age, maternal education, family income, ethnicity
Zellman 2008[73, m, Z]	**Publication:** Report<br>**Design:** Longitudinal<br>**Country:** United States<br>**Sample size:** class 156; child 1368<br>**% Female:** 50<br>**Mean age:** 47.338<br>**Ethnicity:** NR (42% minority)<br>**Mean maternal education:** NR<br>**Mean household income:** $45,400<br>**Child Care Type:** Centre-based programs	ECERS-R Total Score ("Parents and Staff" subscale scale note included) 4.44 (1.6)	CBI-Apathy 2.134 (0.733)<br>CBI-Considerateness 3.489 (0.868)<br>CBI-Creativity 3.737 (0.773)<br>CBI-Dependence 2.447 (0.806)<br>CBI-Distractibility 2.581 (0.87)<br>CBI-Independence 3.79 (0.682)<br>CBI-TO 3.431 (0.872)<br>CBI-Verbal 3.507 (0.879)<br>PPVT-III 92.756 (14.89)<br>WJ-AP 97.419 (14.392)<br>WJ-LWI 104.755 (16.728)<br>WJ-PC 115.707 (13.322)	**Statistics Extracted:** B, SE<br>**Covariates:** child/family level - age at assessment, gender, learning problems, hours per week with provider, duration with provider, family income, maternal education (has a BA), minority status, speaks other language besides English, parents' child-rearing style; classroom level - Head Start program, non-profit organization, level of intervention intensity as determined by Qualistar
Zill 2003[74, s, K]	**Publication:** Report<br>**Design:** Longitudinal<br>**Data set:** FACES 2000<br>**Country:** United States<br>**Sample size:** class 278; child, range by analysis 957-2138<br>**% Female:** NR	ECERS-R Language Reasoning 4.86 (1.2)	Cooperative Behavior 16.58 (4.63)<br>PPVT-III 89.1 (NR)<br>Problem Behavior 1.21 (1.47)<br>WJ-Dictation 87.1 (NR)<br>WJ-LWI 92.9 (NR)	**Statistics Extracted:** B<br>**Covariates:** child/family level - age, sex, ethnicity, language, disability, mother-father family, neither birth parent in home, parent literacy, parent education, family income, welfare status, books in home, frequency of

Description of Studies Meeting Inclusion Criteria[a]

Study[b]	Characteristics	Quality Measures M(SD)[c]	Outcome Measures M(SD)[d]	Covariates
	**Mean age:** NR<br>**Ethnicity:** NR<br>**Mean maternal education:** NR<br>**Mean household income:** NR<br>**Child Care Type:** Head Start			reading to child; classroom level - full-day class, AP individualizing score, ECERS-R Language, CIS, teacher (a) ratio, (b) experience, (c) DAP beliefs score, (d) ethnicity, (e) salary, parent education, family income, proportion non-minority, proportion language minority; program-level - High Scope curriculum, creative curriculum, teacher salary, proportion non-minority children, parent education, family income, proportion language-minority children
Zill 2006[75, K]	**Publication:** Report<br>**Design:** Longitudinal<br>**Data set:** FACES 2000<br>**Country:** United States<br>**Sample size:** class 278; child, range by analysis 674-1729<br>**% Female:** 50<br>**Mean age:** NR<br>**Ethnicity:** White-35%, B32%, A1%, H28%, M3%, O1%<br>**Mean maternal education:** NR<br>**Mean household income:** NR<br>**Child Care Type:** Head Start	ECERS-R Language Reasoning NR	CAP-One-to-one NR<br>CAP-Color naming NR<br>Draw-A-Design NR<br>PPVT-III NR<br>Aggressive 1.49 (1.93)<br>Book Knowledge NR<br>Hyperactive 0.97 (1.4)<br>Social Awareness NR<br>Social Skills 18.12 (4.28)<br>Withdrawn 2.05 (2.4)<br>WJ-AP NR<br>WJ-Dictation NR<br>WJ-LWI NR	**Statistics Extracted:** B<br>**Covariates:** child/family level - age, gender, ethnicity, disability, parent education, family income, welfare status, language-minority family, mother-father family, neither birth parent in home, parent literacy, books in home, frequency of reading to child, one-year head start graduate; classroom-level - ratio, education, experience, teacher ethnicity, teacher salary, teacher beliefs, CIS, parent education, family income level, proportion language-minority, proportion non-minority, full-day class; program-level - parent education, family income, high/scope curriculum, creative curriculum, teacher salary, proportion non-minority children

Abbreviations: NR=Not Reported; C=Caucasian, B=African American, H=Hispanic, A=Asian, M=Mixed, O=Other. For all other acronyms, please refer to Supplemental Information5(SI) for all child outcomes, and SI 6 for all journal, large study, or covariate acronyms.

[a]Descriptives provided reflect characteristics (actual or estimates) of the sample/research design for which data was extracted for the current study and therefore may represent a subsample/analysis of the larger study.

[b]This paper is one of a series of "Meta-Analyses and Systematic Reviews" assessing the relationship between child care quality and children's outcomes; therefore, uppercase superscript letters below are in reference to various large databases that samples in these papers were drawn from. These letters have been kept consistent across the series of papers for our readers.

[c]ECERS/ECERS-R was operationalized in a number of different ways.

[d]Scale of measurement for the means and standard reported in this table varied across studies (e.g., percentiles, standard scores, raw score). All outcomes used in the current paper are presented in SI 4.

[e]All covariates used in the described sample are listed, but may vary by analyses.

[m]Studies included in the meta analyses.

[A]National Center for Early Development and Learning Dataset (NCEDL, 2002, 2004); [B]Head Start Family and Children Experiences Survey (FACES, 2006 Cohort); [C]Bermuda Preschool Study (1980); [D]Cost, Quality and Outcomes Study (CQO, 1993-1994); [E]Effective Preschool and Primary Education Study (EPPE, 1997-1998); [F]Georgia Early Childhood Study (GECS, 2002); [H]Early Head Start

(EHS, 2001-2003 Cohort); [J]Head Start Family and Children Experiences Survey (FACES, 1997) Cohort; [K]Head Start Family and Children Experiences Survey (FACES, 2000 Cohort); [M]Head Start Family and Children Experiences Survey (FACES, 2009 Cohort); [N]Early Childhood Longitudinal Study (ECLS-B, 2001-2006, Birth Cohort); [O]National Child Care Staffing Study (NCCSS, 1988); [R]Northeastern United States sample (Moller and colleagues, 2008; Year NR); [S]8-County Region of North-Central Indiana (Year NR); [T]Otitis Media Study (Year NR); [U]Preschool Curriculum Evaluation Research (PCER, 1999-2003); [V]Five Rural Districts of Bangladesh (2006-2008);[YA]More is Four North Carolina Study (2002-2003) Cohort; [YB]More is Four North Carolina Study (2003-2004) Cohort; [YC]More is Four North Carolina Study (2005-2006) Cohort; [Z]Colorado QRIS.

The Relationship between the Early Childhood Environment Rating Scale and its Revised Form and Child Outcomes: a Systematic Review and Meta-Analysis

References

1. Aboud F. Evaluation of an early childhood preschool program in rural Bangladesh. *Early Child Res Q*. 2006;21(1):46-60. doi:10.1016/j.ecresq.2006.01.008.
2. Aboud F, Hossain K. The impact of preprimary school on primary school achievement in Bangladesh. *Early Child Res Q*. 2011;26(2):237-246. doi:10.1016/j.ecresq.2010.07.001.
3. Abreu-Lima I, Leal T, Cadima J, Gamelas A. Predicting child outcomes from preschool quality in Portugal. *Eur J Psychol Educ*. 2013;28(2):399-420. doi:10.1007/s10212-012-0120-y.
4. Aikens N, Tarullo L, Hulsey L, Ross C, West J, Xue Y. A Year in Head Start: children, families and programs. ACF-ORPRE report. Washington, DC: U.S. Department of Health and Human Services, Administration for Children and Families, Office of Planning, Research and Evaluation; 2010. http://files.eric.ed.gov/fulltext/ED517213.pdf. Accessed July 1, 2015.
5. Hulsey L, Aikens N, Xue Y, Tarullo L, West J. ACF-OPRE report: data tables for FACES 2006: a year in Head Start report. Washington, DC: U.S. Department of Health and Human Services, Administration for Children and Families, Office of Planning, Research and Evaluation; 2010. http://www.acf.hhs.gov/sites/default/files/opre/year_data_tables.pdf.
6. Aikens N, Moiduddin E, Xue Y, Tarullo L, West J. Data tables for child outcomes and classroom quality in FACES 2009 Report. Washington, DC: U.S. Department of Health and Human Services, Administration for Children and Families, Office of Planning, Research and Evaluation; 2012. http://www.acf.hhs.gov/sites/default/files/opre/data_tables_for_child_outcomes_and_classroom_quality_in_faces_2009.pdf. Accessed July 1, 2015.
7. Moiduddin E, Aikens N, Tarullo L, West J, Xue Y. Child outcomes and classroom quality in FACES 2009. OPRE Report 2012-37a. Washington, DC: U.S. Department of Health and Human Services, Administration for Children and Families, Office of Planning, Research and Evaluation; 2012. http://eric.ed.gov/?id=ED539265.
8. Anders Y, Rossbach H, Weinert S, et al. Home and preschool learning environments and their relations to the development of early numeracy skills. *Early Child Res Q*. 2012;27(2):231-244. doi:10.1016/j.ecresq.2011.08.003.
9. Assel M, Landry S, Swank P. Are early childhood classrooms preparing children to be school ready? The circle teacher behavior rating scale. In: Justice L, Vukelic C, eds. *Achieving Excellence in Preschool Literacy Instruction*. New York, NY: Guilford Press; 2008:120-135.
10. Auger A, Farkas G, Burchinal M, Duncan G, Vandell D. Preschool center care quality effects on academic achievement: an instrumental variables analysis. *Dev Psychol*. 2014;50(12):2559-2571. doi:10.1037/a0037995.
11. Barnett W, Yarosz D, Thomas J, Jung K, Blanco D. Two-way and monolingual English immersion in preschool education: an experimental comparison. *Early Child Res Q*. 2007;22(3):277-293. doi:10.1016/j.ecresq.2007.03.003.
12. Bryant D, Burchinal M, Lau L, Sparling J. Family and classroom correlates of Head Start children’s developmental outcomes. *Early Child Res Q*. 1994;9(3-4):289-304. doi:10.1016/0885-2006(94)90011-6.

13. Bryant D, Maxwell K, Taylor K, Poe M, Peisner-Feinberg E, Bernier K. Smart Start and preschool child care quality in North Carolina: change over time and relation to children's readiness. Chapel Hill, NC: FPG Child Development Institute; 2003. http://files.eric.ed.gov/fulltext/ED473699.pdf.
14. Burchinal M, Nelson L. Family selection and child care experiences: implications for studies of child outcomes. *Early Child Res Q*. 2000;15(3):385-411. doi:10.1016/S0885-2006(00)00072-7.
15. Burchinal M, Peisner-Feinberg E, Bryant D, Clifford R. Children's social and cognitive development and child-care quality: testing for differential associations related to poverty, gender, or ethnicity. *Appl Dev Sci*. 2000;4(3):149-165. doi:10.1207/S1532480XADS0403_4.
16. Burchinal M, Roberts J, Riggins Jr R, Zeisel S, Neebe E, Bryant D. Relating quality of center-based child care to early cognitive and language development longitudinally. *Child Dev*. 2000;71(2):339-357. doi:10.1111/1467-8624.00149.
17. Burchinal M, Roberts J, Zeisel S, Hennon E, Hooper S. Social risk and protective child, parenting, and child care factors in early elementary school years. *Parenting*. 2006;6(1):79-113. doi:10.1207/s15327922par0601_4.
18. Burchinal M, Howes C, Pianta R, et al. Predicting child outcomes at the end of kindergarten from the quality of pre-kindergarten teacher–child interactions and instruction. *Appl Dev Sci*. 2008;12(3):140-153. doi:10.1080/10888690802199418.
19. Burchinal M, Kainz K, Cai Y. How well do our measures of quality predict child outcomes? A meta-analysis and coordinated analysis of data from large-scale studies of early childhood settings. In: Zaslow M, Martinez-Beck I, Tout K, Halle T, eds. *Quality Measurement in Early Childhood Settings*. Baltimore, MD: Paul H Brookes Publishing; 2011:11-31.
20. Chang F, Crawford G, Early D, et al. Spanish-speaking children's social and language development in pre-kindergarten classrooms. *Early Educ Dev*. 2007;18(2):243-269. doi:10.1080/10409280701282959.
21. Chin-Quee D, Scarr S. Lack of early child care effects on school-age children's social competence and academic achievement. *Early Dev Parent*. 1994;3(2):103-112. doi:10.1002/edp.2430030207.
22. Clawson C, Luze G. Individual experiences of children with and without disabilities in early childhood settings. *Top Early Child Spec Educ*. 2008;28(3):132-147. doi:10.1177/0271121407311482.
23. Dang T, Farkas G, Burchinal M, et al. Preschool center quality and school readiness: quality main effects and variation by demographic and child characteristics. Evanston, IL: Society for Research on Educational Effectiveness; 2011. http://eric.ed.gov/?id=ED519004.
24. Dickinson D, Tabors P, eds. *Beginning Literacy with Language: Young Children Learning at Home and School*. Baltimore, MD: Brookes Publishing; 2001.
25. Dotterer A, Burchinal M, Bryant D, Early D, Pianta R. Universal and targeted pre-kindergarten programmes: a comparison of classroom characteristics and child outcomes. *Early Child Dev Care*. 2012;183(7):931-950. doi:10.1080/03004430.2012.698388.
26. Dunn L. Proximal and distal features of day care quality and children's development. *Early Child Res Q*. 1993;8(2):167-192. doi:10.1016/S0885-2006(05)80089-4.

27. Dunn L, Beach S, Kontos S. Quality of the literacy environment in day care and children's development. *J Res Child Educ*. 1994;9(1):24-34. doi:10.1080/02568549409594950.
28. Early D, Bryant D, Pianta R, et al. Are teachers' education, major, and credentials related to classroom quality and children's academic gains in pre-kindergarten? *Early Child Res Q*. 2006;21(2):174-195. doi:10.1016/j.ecresq.2006.04.004.
29. Epstein A. *Training for Quality: Improving Early Childhood Programs through Systematic Inservice Training*. High/Scope Educational Research Foundation, Ypsilanti, MI.; 1993.
30. Fiorentino L, Howe N. Language competence, narrative ability, and school readiness in low-income preschool children. *Can J Behav Sci Can Sci Comport*. 2004;36(4):280-294. doi:10.1037/h0087237.
31. Goelman H, Pence A. Children in three types of day care: daily experiences, quality of care and developmental outcomes. *Early Child Dev Care*. 1988;33(1-4):67-76. doi:10.1080/0300443880330105.
32. Gordon R, Fujimoto K, Kaestner R, Korenman S, Abner K. An assessment of the validity of the ECERS–R with implications for measures of child care quality and relations to child development. *Dev Pscyhology*. 2013;49(1):146-160. doi:10.1037/a0027899.
33. Henry G, Henderson L, Ponder B, Gordon C, Mashburn A, Rickman D. Report of the findings from the Early Childhood Study: 2001-02. Georgia State University, School of Policy Studies; 2003:Atlanta, GA. http://eric.ed.gov/?id=ED481261.
34. Henry G, Rickman D, Ponder B, Henderson L, Mashburn A, Gordon C. The Georgia Early Childhood Study. Atlanta, GA: Georgia State University, School of Policy Studies; 2005.
35. Herrera M, Mathiesen M, Merino J, Recart I. Learning contexts for young children in Chile: process quality assessment in preschool centres. *Int J Early Years Educ*. 2005;13(1):13-27. doi:10.1080/09669760500048253.
36. Hestenes L, Kintner-Duffy V, Wang Y, et al. Comparisons among quality measures in child care settings: understanding the use of multiple measures in North Carolina's QRIS and their links to social-emotional development in preschool children. *Early Child Res Q*. 2015;30:199-214. doi:10.1016/j.ecresq.2014.06.003.
37. Hindman A, Skibbe L, Miller A, Zimmerman M. Ecological contexts and early learning: contributions of child, family, and classroom factors during Head Start, to literacy and mathematics growth through first grade. *Early Child Res Q*. 2010;25(2):235-250. doi:10.1016/j.ecresq.2009.11.003.
38. Howes C, Sakai L, Shinn M, Phillips D, Galinsky E, Whitebook M. Race, social class, and maternal working conditions as influences on children's development. *J Appl Dev Psychol*. 1995;16(1):107-124. doi:10.1016/0193-3973(95)90019-5.
39. Howes C, Burchinal M, Pianta R, et al. Ready to learn? Children's pre-academic achievement in pre-kindergarten programs. *Early Child Res Q*. 2008;23(1):27-50. doi:10.1016/j.ecresq.2007.05.002.
40. Jackson B, Larzelere R, St. Clair L, Corr M, Fichter C, Egertson H. The impact of HeadsUp! reading on early childhood educators' literacy practices and preschool children's literacy skills. *Early Child Res Q*. 2006;21(2):213-226. doi:10.1016/j.ecresq.2006.04.005.
41. Jeon H, Langill C, Peterson C, Luze G, Carta J, Atwater J. Children's individual experiences in early care and education:

relations with overall classroom quality and children's school readiness. *Early Educ Dev*. 2010;21(6):912-939. doi:10.1080/10409280903292500.

42. Keys T, Farkas G, Burchinal M, et al. Preschool center quality and school readiness: quality effects and variation by demographic and child characteristics. *Child Dev*. 2013;84(4):1171-1190. doi:10.1111/cdev.12048.
43. Kontos S. Child care quality, family background, and children's development. *Early Child Res Q*. 1991;6(2):249-262. doi:10.1016/0885-2006(91)90011-9.
44. Kwan C, Sylva K, Reeves B. Day care quality and child development in Singapore. *Early Child Dev Care*. 1998;144(1):69-77. doi:10.1080/0300443981440108.
45. Le V, Schaack D, Setodji C. Identifying baseline and ceiling thresholds within the Qualistar Early Learning Quality Rating and Improvement System. *Early Child Res Q*. 2015;30:215-226. doi:10.1016/j.ecresq.2014.03.003.
46. Lyon M, Canning P. *Atlantic Day Care Study*. Halifax: Mount Saint Vincent University; 1995.
47. Mashburn A. Quality of social and physical environments in preschools and children's development of academic, language, and literacy skills. *Appl Dev Sci*. 2008;12(3):103-127. doi:10.1080/10888690802199392.
48. Mashburn A, Pianta R, Hamre B, et al. Measures of classroom quality in prekindergarten and children's development of academic, language, and social skills. *Child Dev*. 2008;79(3):732-749. doi:10.1111/j.1467-8624.2008.01154.x.
49. McCartney K, Scarr S, Grajek S, Schwarz J. Environmental differences among day care centers and their effects on children's development. In: Zigler E, Gordon E, eds. *Day Care: Scientific and Social Policy Issues*. Boston, MA: Auburn House Publishing Company; 1982.
50. McCartney K. Effect of quality of day care environment on children's language development. *Dev Psychol*. 1984;20(2):244-260. doi:10.1037/0012-1649.20.2.244.
51. McWayne C, Fantuzzo J, McDermott P. Preschool competency in context: an investigation of the unique contribution of child competencies to early academic success. *Dev Psychol*. 2004;40(4):633-645. doi:10.1037/0012-1649.40.4.633.
52. Moller A, Forbes-Jones E, Hightower A. Classroom age composition and developmental change in 70 urban preschool classrooms. *J Educ Psychol*. 2008;100(4):741-753. doi:10.1037/a0013099.
53. Moller A, Forbes-Jones E, Hightower A, Friedman R. The developmental influence of sex composition in preschool classrooms: boys fare worse in preschool classrooms with more boys. *Early Child Res Q*. 2008;23(3):409-418. doi:10.1016/j.ecresq.2008.05.001.
54. Montes G, Hightower A, Brugger L, Moustafa E. Quality child care and socio-emotional risk factors: no evidence of diminishing returns for urban children. *Early Child Res Q*. 2005;20(3):361-372. doi:10.1016/j.ecresq.2005.07.006.
55. Peisner-Feinberg E, Burchinal M. Relations between preschool children's child-care experiences and concurrent development: the cost, quality, and outcomes study. *Merrill-Palmer Q*. 1997;43(3):451-477.
56. Peisner-Feinberg E, Burchinal M, Clifford R, et al. The children of the Cost, Quality, and Outcomes Study go to school: technical report. Chapel Hill, NC: FGP Child Development Center; 1999.

http://fpg.unc.edu/sites/fpg.unc.edu/files/resources/reports-and-policy-briefs/NCEDL_CQO_technical_report.pdf.
57. Peisner-Feinberg E, Maris C, More at Four Evaluation team. Evaluation of the North Carolina More at Four pre-kindergarten program: children's longitudinal outcomes and classroom quality in kindergarten. Chapel Hill, NC: FPG Child Development Institute; 2006. http://fpg.unc.edu/sites/fpg.unc.edu/files/resources/reports-and-policy-briefs/MAF_Yr4_pt2_full_report.pdf.
58. Peisner-Feinberg E, Schaaf J, The More at Four evaluation team. Children's outcomes & program quality in the fifth year. Evaluation of the North Carolina More at Four pre-kindergarten program, year 5 report (July 1, 2005-June 30, 2006). Chapel Hill, NC: FPG Child Development Institute, University of North Carolina; 2007. http://eric.ed.gov/?id=ED499809.
59. Peisner-Feinberg E, Schaaf J. Evaluation of the North Carolina More at Four pre-kindergarten program year 6 report (July 1, 2006-June 30, 2007): Children's longitudinal outcomes and program quality over time (2003-2007). Chapel Hill, NC: FPG Child Development Institute; 2008. http://ea.niusileadscape.org/docs/FINAL_PRODUCTS/LearningCarousel/maf_Yr6_rpt.pdf.
60. Peisner-Feinberg E, Schaaf J. Evaluation of the North Carolina More at Four pre-kindergarten program year 7 report (2007-2008): rerformance and progress in the seventh year (2007-2008). Chapel Hill, NC: FPG Child Development Institute. University of North Carolina; 2008. http://www.ncga.state.nc.us/documentsites/committees/JLEOC/Reports%20Received/Archives/2009%20Reports%20Received/More%20At%20Four%20Program%20Review/Year%207%20Performance%20and%20Progress.pdf. Accessed November 24, 2015.
61. Peisner-Feinberg E, Schaaf J, LaForett D. Children's growth and classroom experiences in Georgia's pre-k program: findings from the 2011–2012 evaluation study. Chapel Hill, NC: FPG Child Development Institute; 2013. http://files.eric.ed.gov/fulltext/ED541933.pdf.
62. Phillips D, McCartney K, Scarr S. Child-care quality and children's social development. *Dev Psychol*. 1987;23(4):537-543. doi:10.1037/0012-1649.23.4.537.
63. Pinto A, Pessanha M, Aguiar C. Effects of home environment and center-based child care quality on children's language, communication, and literacy outcomes. *Early Child Res Q*. 2013;28(1):94-101. doi:10.1016/j.ecresq.2012.07.001.
64. Reid J, Ready D. High-quality preschool: the socioeconomic composition of preschool classrooms and children's learning. *Early Educ Dev*. 2013;24(8):1082-1111. doi:10.1080/10409289.2012.757519.
65. Sabol T, Hong S, Pianta R, Burchinal M. Can rating pre-k programs predict children's learning? *Science*. 2013;341(6148):845-846. doi:10.1126/science.1233517.
66. Sabol T, Pianta R. Do standard measures of preschool quality used in statewide policy predict school readiness? *Educ Finance Policy*. 2014;9(2):116-164. doi:10.1162/EDFP_a_00127.
67. Schliecker E, White D, Jacobs E. The role of day care quality in the prediction of children's vocabulary. *Can J Behav Sci Can Sci Comport*. 1991;23(1):12-24. doi:10.1037/h0078960.
68. Seppanen P, Godin K, Metzger J, Bronson M, Cichon D. Observational study of early childhood programs. Dover, NH:

Development of Assistance Corp.; 1993:222. http://files.eric.ed.gov/fulltext/ED366469.pdf.
69. Sylva K, Siraj-Blatchford I, Taggart B, et al. Capturing quality in early childhood through environmental rating scales. *Early Child Res Q*. 2006;21(1):76-92. doi:10.1016/j.ecresq.2006.01.003.
70. Weiland C, Ulvestad K, Sachs J, Yoshikawa H. Associations between classroom quality and children's vocabulary and executive function skills in an urban public prekindergarten program. *Early Child Res Q*. 2013;28(2):199-209. doi:10.1016/j.ecresq.2012.12.002.
71. West J, Malone L, Hulsey L, Aikens N, Tarullo L. ACF-OPRE report: Head Start children go to kindergarten. Washington, DC: U.S. Department of Health and Human Services, Administration for Children and Families, Office of Planning, Research and Evaluation; 2010. http://www.acf.hhs.gov/sites/default/files/opre/hs_kindergarten.pdf.
72. Whitebook M, Howes C, Phillips D. Who cares? Child care teachers and the quality of care in America. Final report National Child Care Staffing Study. Berkely, CA: Child Care Employee Project; 1989:41-45.
73. Zellman G, Perlman M, Le V, Setodji C. Assessing the validity of the Qualistar Early Learning Quality Rating and Improvement System as a tool for improving child-care quality. Santa Monica, CA: RAND Education; 2008. http://www.rand.org/content/dam/rand/pubs/monographs/2008/RAND_MG650.pdf.
74. Zill N, Resnick G, Kim K, et al. Head Start FACES 2000: A whole-child perspective on program performance. Fourth progress report. Washington, DC: U.S. Department of Health and Human Services, Administration for Children and Families, Office of Planning, Research and Evaluation; 2003. http://eric.ed.gov/?id=ED478791.
75. Zill N, Resnick G, Kim K, et al. Head Start performance measures center Family and Child Experiences Survey (FACES 2000): technical report. Washington, DC: U.S. Department of Health and Human Services, Administration for Children and Families, Office of Planning, Research and Evaluation; 2006. http://www.acf.hhs.gov/sites/default/files/opre/tech2k_final2.pdf.
