# Supplementary material for: The relationship between the Early Childhood Environment Rating Scale and its revised form and child outcomes: A systematic review and meta-analysis
Source: PLoS One. 2017 Jun 6;12(6):e0178512. doi: 10.1371/journal.pone.0178512 (PMC5461062; doi:10.1371/journal.pone.0178512)
Supplement: S4 File — (PDF) [file pone.0178512.s004.pdf]

The Relationship between the Early Childhood Environment Rating Scale and its Revised Form and Child Outcomes: a Systematic Review and Meta-Analysis

Supplemental Information 4

List of Child Outcome Variables

Outcome Category	Number of Variables	Child Outcome Variable	Number of Studies	Number of Samples
Approach	n=8	Adaptive Social Behavior Inventory – Independence and Concentration (Teacher, ASBI)	1	1
		Child Behavior Inventory - Creativity (Teacher, CBI)	1	1
		Child Behavior Inventory - Dependence (Teacher, CBI)	3	3
		Child Behavior Inventory - Dependence (Parent, CBI)		
		Child Behavior Inventory - Distractibility (Teacher, CBI)	1	1
		Child Behavior Inventory - Independent (Teacher, CBI)	1	1
		Child Behavior Inventory - Task Orientation (Teacher, CBI)	4	4
		Child Behavior Inventory - Task Orientation (Parent, CBI)		
		Child Observation Record - Initiative (COR)	1	1
		Teacher Child Rating Scale - Task orientation (percent at risk) (Teacher, TCRS)	1	1
Cognitive	n=21	Academic Achievement (Teacher)	1	2
		Backward Digit Span	1	1
		Bayley Scales of Infant Development: Mental Development Index (BSID)	1	1
		British Ability Scales-II - Pattern Construction	1	1
		British Ability Scales-II - Picture Similarities	1	1
		Child Behavior Inventory - Intelligence (Parent, CBI)	6	6
		Child Behavior Inventory - Intelligence (Teacher, CBI)		
		Child Observation Record - Cognitive (Teacher, COR)	2	2
		Cognitive (author created)	1	1
		Developmental Indicators for the Assessment of Learning - Revised - Concepts (DIAL-R)	1	1
		FACES – Social Awareness Task	6	6
		Forward Digit Span	1	1
		Identifying Colours (also referred to as Color Knowledge; Colour Naming Task)	5	5
		Kaufman Assessment Battery for Children – Achievement (K-ABC)	2	3
		Kaufman Assessment Battery for Children - Mental Processing (K-ABC)	1	2
		Pictorial Scale of Perceived Competence and Acceptance for Young Children - Cognitive Competence (Teacher)	2	2
		Preschool Inventory - Revised (PSI-R)	4	5
		School Readiness	1	1
		Skills Mastery	1	1
		Slosson Intelligence Test	1	1
		Wechsler Preschool Primary Scale of Intelligence III - Matrix Reasoning (WPPSI-III)	1	1
		Wechsler Preschool Primary Scale of Intelligence III -	1	1

The Relationship between the Early Childhood Environment Rating Scale and its Revised Form and Child Outcomes: a Systematic Review and Meta-Analysis

List of Child Outcome Variables				
**Outcome Category**	**Number of Variables**	**Child Outcome Variable**	**Number of Studies**	**Number of Samples**
		Similarities (WPPSI-III)		
Combination	n=10	Academic Skills (Woodcock Johnson - Applied Problems and Letter Word Identification)	1	1
		Child Behavior Inventory - Cognitive/Attention (Teacher, CBI)	3	3
		Child Behavior Rating Scale (Teacher, CBRS)	1	1
		Child Observation Record - Representation (COR)	1	1
		Child Observation Record - Total (Teacher, COR)	3	3
		Competency Profiles (i.e., Classroom Competency patterns) (Teacher)	1	1
		Developmental Indicators for the Assessment of Learning - Revised - Total (DIAL-R)	1	1
		Early Development Instrument -Total (Teacher)	1	1
		Early Screening Inventory - Revised, Kindergarten Version	1	1
		Vineland - Adaptive Behavior Scale (Teacher, VABS)	1	1
Language	n=52	Academic Rating Scale: Language & Literacy (ARS)	7	7
		Adaptive Language Inventory (Teacher, ALI)	5	5
		British Ability Scales-II - Language (BAS-II)	1	1
		British Ability Scales-II - Verbal Fluency (BAS-II)	1	1
		British Ability Scales-II - Word Reading (BAS-II)	1	1
		Child Observation Record - Language (COR)	1	1
		Cross-Linguistic Assessment of Foundation Level - Letter identification (CLAF) – Letter Identification	1	1
		Communication Task	1	1
		Comprehensive Assessment Program (CAP) Early Childhood Diagnostic Instrument - Emergent Literature (Mason & Stewart, 1989 ver.)	1	1
		Developmental Indicators for the Assessment of Learning - Revised - Language (DIAL-R)	1	1
		Expressive One Word Picture Vocabulary Test (English, EOWPVT)	2	2
		Expressive One Word Picture Vocabulary Test (Spanish, EOWPVTSBE)		
		Griffiths – Language	1	1
		Identifying Letters (also referred to as Alphabet Recognition, Letter Identification; Letter Knowledge; Naming Letters; Letter Naming Task)	11	12
		Language Skills (composite of Peabody Picture Vocabulary Test & Oral and Written Language Scales)	1	1
		Language Skills (composite of Peabody Picture Vocabulary Test and the Clinical Evaluation of Language Fundamentals–Preschool)	1	1
		MacArthur Story Stem Battery - Information Total (MSSB)	1	1
		Oral & Written Language Scales - Oral Expression Scale (OWLS)	6	6
		Oral & Written Language Scales - Total Scale (OWLS)	2	2
		Phonological Awareness Test (PAT)	1	1
		Peabody Picture Vocabulary Test (PPVT-III)	45	56

The Relationship between the Early Childhood Environment Rating Scale and its Revised Form and Child Outcomes: a Systematic Review and Meta-Analysis

List of Child Outcome Variables				
**Outcome Category**	**Number of Variables**	**Child Outcome Variable**	**Number of Studies**	**Number of Samples**
		Test de Vocabulario en Imagenes Peabody (Spanish, TVIP)		
		Test de vocabulario en imágenes (TEVI) (unstandardized measure similar to TVIP, used in Chile)		
		Phoneme Deletion (English)	1	2
		Phoneme Deletion (Spanish)		
		Pre-Reading Composite	1	1
		PreLAS 2000 - Simon Says, Art Show & Human Body (Pre-LAS 2000)	2	2
		Preschool Language Assessment Instrument (PLAI-3)	2	2
		Reading (Grade 1)	1	1
		Rhyme Recognition (English)	1	2
		Rhyme Recognition (Spanish)		
		School-Home Early Language and Literacy Battery-Kindergarten - Formal Definitions (SHELL-K)	1	1
		School-Home Early Language and Literacy Battery-Kindergarten - Narrative Production (SHELL-K)	1	1
		Sequenced Inventory of Communication Development-Revised - Expressive Communication Age (SICD-R)	1	1
		Sequenced Inventory of Communication Development-Revised -Receptive Communication Age (SICD-R)	1	1
		Story and Print Concepts – Total Score	8	8
		Story and Print Concepts - Book Knowledge	2	2
		Story and Print Concepts - Print Awareness	1	2
		Story and Print Concepts - Story Comprehension	1	1
		Teacher Rating of Oral Language and Literacy (Teacher, TROLL)	1	1
		Test of Early Language Development (TELD)	1	1
		Test of Early Reading Ability - Reading Quotient (TERA-3)	1	1
		Test of Early Reading Ability-III: Alphabet and Conventions (TERA-3)	1	1
		Test of Preschool Early Literacy - Phonological Awareness (TOPEL)	1	1
		Test of Preschool Early Literacy - Print Knowledge (TOPEL)	1	1
		Vineland Adaptive Behavior Scales - Communication (VABS)	3	4
		Wechsler Preschool Primary Scale of Intelligence III - Vocabulary (WPPSI-III)	1	1
		Woodcock Johnson- Dictation (WJ)	2	2
		Woodcock Johnson- Letter Word Identification (Spanish, WM)	16	20
		Woodcock Johnson- Letter Word Identification (WJ)		
		Woodcock Johnson- Passage Comprehension (WJ)	2	2
		Woodcock Johnson- Picture Vocabulary (English, WJ)	2	3
		Bateria psico-educativa revisada de woodcock-Munoz - Revisada - Picture Vocabulary (Spanish, WM)		
		Woodcock Johnson- Rhyming (English, WJ)	7	7
		Woodcock Johnson- Rhyming (Spanish, WM)		

The Relationship between the Early Childhood Environment Rating Scale and its Revised Form and Child Outcomes: a Systematic Review and Meta-Analysis

List of Child Outcome Variables				
**Outcome Category**	**Number of Variables**	**Child Outcome Variable**	**Number of Studies**	**Number of Samples**
		Woodcock Johnson- Sound Awareness (WJ)	1	1
		Woodcock Johnson- Spelling (English, WJ)	1	1
		Woodcock Johnson- Spelling (Spanish, WM)		
		Woodcock Johnson- Word Attack (WJ)	1	1
		Woodcock-Munoz Language Survey - Broad Abilities (Spanish, WM)	1	1
		Writing (Grade 1)	1	1
Math	n=9	British Ability Scales-II – Early Number Concepts	1	1
		Child Observation Record - Logic & Math (COR)	1	1
		Counting Task (also referred to as One-to-One Counting)	8	8
		Early Childhood Longitudinal Study-Birth Cohort - Math (ECLS-B)	7	7
		Identifying Numbers (also referred to as Naming Numbers)	3	3
		Kaufman Assessment Battery for Children - Arithmetic (German, K-ABC)	1	1
		Math (Grade 1)	1	1
		Wechsler Preschool Primary Scale of Intelligence III - Math (WPPSI-III)	1	1
		Woodcock Johnson- Applied Problems (English, WJ)	31	38
		Bateria psico-educativa revisada de woodcock-Munoz - Revisada- Applied Problems (Spanish, WM)		
Physical	n=5	Child Observation Record - Motor (Teacher, COR)	2	2
		Child Observation Record - Music & Movement (COR)	1	1
		Design Copying	1	1
		Developmental Indicators for the Assessment of Learning - Revised - Motor (DIAL-R)	1	1
		Pictorial Scale of Perceived Competence and Social Acceptance for Young Children - Physical Competence (Child)	1	1
		Pictorial Scale of Perceived Competence and Social Acceptance for Young Children - Physical Competence (Teacher)		
Positive Behavior	n=34	Adaptive Social Behavior Inventory - Comply (Teacher, ASBI)	1	2
		Adaptive Social Behavior Inventory - Cooperation and Conformity (Teacher, ASBI)	1	1
		Adaptive Social Behavior Inventory - Express (Teacher, ASBI)	1	2
		Adaptive Social Behavior Inventory - Peer Sociability (Teacher, ASBI)	1	1
		Adult Orientation (Teacher, author created)	1	1
		Attitudes/Perceptions of Competence (shortened version)	1	1
		Behavior Problems Index - Social Skills (Teacher, BPI)	1	2
		Child Behavior Inventory - Considerateness (Teacher, CBI)	4	4
		Child Behavior Inventory - Considerateness (Parent, CBI)		

The Relationship between the Early Childhood Environment Rating Scale and its Revised Form and Child Outcomes: a Systematic Review and Meta-Analysis

List of Child Outcome Variables				
**Outcome Category**	**Number of Variables**	**Child Outcome Variable**	**Number of Studies**	**Number of Samples**
		Child Behavior Inventory - Sociability (Teacher, CBI)	7	7
		Child Behavior Inventory - Sociability (Parent, CBI)		
		Child Observation Record - Social Relations (Teacher, COR)	3	3
		Cooperative Behavior (Teacher)	1	1
		Early Childhood Longitudinal Study-Birth Cohort – Attention and Concentration (ECLS-B)	1	1
		Early Childhood Longitudinal Study-Birth Cohort – Emotional and Behavioral Regulation (ECLS-B)	1	1
		Early Childhood Longitudinal Study-Birth Cohort - Prosocial (Parent, ECLS-B)	3	3
		Early Childhood Longitudinal Study-Birth Cohort - Prosocial (Teacher, ECLS-B)		
		Entwistle Scale of Personal Maturity	2	2
		Executive Functioning Pencil Tapping Task	1	1
		Pictorial Scale of Perceived Competence and Social Acceptance for Young Children - Cognitive Competence (Child)	2	2
		Pictorial Scale of Perceived Competence and Social Acceptance for Young Children - Maternal Acceptance (Child)	1	1
		Pictorial Scale of Perceived Competence and Social Acceptance for Young Children - Peer Acceptance (Child)	2	2
		Pictorial Scale of Perceived Competence and Social Acceptance for Young Children - Peer Acceptance (Teacher)		
		Social Competence (author created versions)	4	4
		Social Skills and Behavior Problems Scale - Social Competence (Teacher, SSBS)	1	1
		Social Skills Improvement System – Self-Efficacy (Teacher, SSIS)	1	1
		Social Skills Improvement System - Social Skills (Teacher, SSIS)	2	2
		Social Skills Rating System - Social Skills (Parent, SSRS)	10	10
		Social Skills Rating System - Social Skills (Teacher, SSRS)		
		Student-Teacher Relationship Scale (Teacher, STRS)	1	1
		Teacher Child Rating Scale - Assertive Social skills (Teacher, TCRS)	1	1
		Teacher Child Rating Scale - Behavior Control (Teacher, TCRS)	1	1
		Teacher Child Rating Scale - Decreased risk status (Teacher, TCRS)	1	1
		Teacher Child Rating Scale - Peer sociability (Teacher, TCRS)	1	1
		Teacher Child Rating Scale - Percent not at risk (Teacher, TCRS)	1	1
		Teacher Child Rating Scale - Social Competence	5	6

List of Child Outcome Variables				
**Outcome Category**	**Number of Variables**	**Child Outcome Variable**	**Number of Studies**	**Number of Samples**
		(Teacher, TCRS)		
**Problem Behavior**	n=28	Adaptive Social Behavior Inventory – Antisocial/Worried (Teacher, ASBI)	1	1
		Adaptive Social Behavior Inventory - Disrupt (Teacher, ASBI)	2	4
		Behavior Problems Index - Problem Beh (Teacher, BPI)	1	2
		Behavior Problems (author created)	1	1
		Child Behavior Checklist- Aggressive (Teacher, CBCL)	1	1
		Child Behavior Inventory - Apathy (Teacher, CBI)	1	1
		Child Behavior Inventory - Behavior Problems (Teacher, CBI)	4	4
		Child Behavior Inventory - Hostility (Teacher, CBI)	1	1
		Early Childhood Longitudinal Study-Birth Cohort – Externalizing (ECLS-B)	2	2
		FACES - Aggressive Behavior (Parent)	1	1
		FACES - Aggressive Behavior (Teacher)		
		FACES - Hyperactive Behavior (Parent)	1	1
		FACES - Hyperactive Behavior (Teacher)		
		FACES - Withdrawn Behavior (Parent)	1	1
		FACES - Withdrawn Behavior (Teacher)		
		Preschool Behavior Questionnaire - Aggressiveness (Teacher, PBQ)	2	2
		Preschool Behavior Questionnaire – Aggressiveness (Parent, PBQ)		
		Preschool Behavior Questionnaire - Aggressiveness-Hostile (PBQ)	1	1
		Preschool Behavior Questionnaire - Anxiety (Teacher, PBQ)	2	2
		Preschool Behavior Questionnaire - Anxiety (Parent, PBQ)		
		Preschool Behavior Questionnaire - Hyperactivity (Teacher, PBQ)	2	2
		Preschool Behavior Questionnaire - Hyperactivity (Parent, PBQ)		
		Preschool Behavior Questionnaire - Hyperactivity-Distractible (Parent, PBQ)	3	3
		Preschool Behavior Questionnaire - Total Maladjustment (Teacher, PBQ)	1	1
		Social Skills and Behavior Problems Scale - Behavior Problems (Teacher, SSBS)	1	1
		Social Skills Improvement System - Externalizing Problems (Teacher, SSIS)	1	1
		Social Skills Improvement System - Internalizing Problems (Teacher, SSIS)	1	1
		Social Skills Improvement System - Problem Behavior (Teacher, SSIS)	1	1
		Social Skills Rating System - Problem Behaviors (Teacher, SSRS)	6	6
		Teacher Child Rating Scale - Behavior Problems (Teacher, TCRS)	5	5

List of Child Outcome Variables				
**Outcome Category**	**Number of Variables**	**Child Outcome Variable**	**Number of Studies**	**Number of Samples**
		Teacher Child Rating Scale - Change in risk status (Teacher, TCRS)	1	1
		Teacher Child Rating Scale - Increased risk status (Teacher, TCRS)	1	1
		Teacher Child Rating Scale - Multiple risk (Teacher, TCRS)	1	1
		Teacher Child Rating Scale - Socio-emotional Risk (Teacher, TCRS)	1	1
		Teacher Report Form – Problem Behavior (Teacher, TRF)	1	1
**Total**	**n=168**			
