# Supplementary material for: The relationship between the Early Childhood Environment Rating Scale and its revised form and child outcomes: A systematic review and meta-analysis
Source: PLoS One. 2017 Jun 6;12(6):e0178512. doi: 10.1371/journal.pone.0178512 (PMC5461062; doi:10.1371/journal.pone.0178512)
Supplement: S5 File — (PDF) [file pone.0178512.s005.pdf]

The Relationship between the Early Childhood Environment Rating Scale and its Revised Form and Child Outcomes: a Systematic Review and Meta-Analysis

Supplemental Information 5
Systematic Review Results: All Outcomes

Table A. ECERS/ECERS-R Predicting all Approach & Combination Outcomes

ECERS/ECERS-R Measurement Level		STUDY[a, b]	Approach[c]								COMBO								
			ASBI – Independence and Concentration	CBI - Creativity	CBI - Dependence	CBI - Distractibility	CBI - Independence	CBI - Task Orientation	COR - Initiative	TCRS - Task Orientation	Academic Skills (Composite)	CBI - Cognitive/Attention	Child Behavior Rating Scale	Competency Patterns Profiles	COR - Representation	COR - Total	DIAL-R - Total	Early Development Instrument	Early Screening Inventory-K
ECERS	Total Score	Burchinal 2011[19] – CQO[D]						⌘											
		Epstein 1993[29]							★						★	★	★		
		McCartney 1982[49, C]			▰★			■★											
	Total Score (Parents and Staff Subscale not included)	Peisner-Feinberg 1997[55,D]										★							
		Phillips 1987[62, C]			↓↓			↓↓											
		Seppanen 1993[68]											⌘						
	Mean of 5 items	Peisner-Feinberg 1999[56, D]										★ ↓							
ECERS-R	Total Score	Abreu-Lima 2013[3]										■ ★							
		Fiorentino 2004[30]																●	
		McWayne 2004[51]												●					○
		Moller… Friedman 2008[53,R]														■			
		Moller… Hightower 2008[52,R]														■			
		Montes 2005[54]								●									
		Sylva 2006[69,E]	■																

The Relationship between the Early Childhood Environment Rating Scale and its Revised Form and Child Outcomes: a Systematic Review and Meta-Analysis

	**Total Score (Parents and Staff Subscale not included)**	Mashburn 2008[47, F]								■ (grey)									
		Zellman 2008[73, Z]		■ (grey)	■ (grey)	■ (grey)	■ (grey)	■ (grey)											
**ECERS Subscales**	**Creative Activities**	Epstein 1993[29]							★ (grey)						★	★ (grey)	★		
	**Fine and Gross Motor Activities**	Epstein 1993[29]							★ (grey)						★	★ (grey)	★ (grey)		
	**Furnishings and Display**	Epstein 1993[29]							★ (grey)						★ (grey)	★ (grey)	★ (grey)		
	**Language-Reasoning**	Epstein 1993[29]							★ (grey)						★	★ (grey)	★ (grey)		
	**Personal Care Routines**	Epstein 1993[29]							★ (grey)						★ (grey)	★ (grey)	★ (grey)		
	**Social Development**	Epstein 1993[29]							★ (grey)						★ (grey)	★ (grey)	★ (grey)		
**ECERS-R Subscales**	**Activities**	Sylva 2006[69,E]	■ (grey)																
	**Interactions**	Sylva 2006[69,E]	■																
	**Language-Reasoning**	Sylva 2006[69,E]	■ (grey)																
	**Parents & Staff**	Sylva 2006[69,E]	■ (grey)																
	**Personal Care Routine**	Sylva 2006[69,E]	■ (grey)																
	**Program Structure**	Sylva 2006[69,E]	■ (grey)																
	**Space and Furnishings**	Sylva 2006[69,E]	■ (grey)																

Legend for Table

**Significant and Positive**	**Significant and Negative**	**Non-Significant**	**Statistic**
★	*★*	★ (grey)	r - Zero Order Pearson's Correlation
**O**	***O***	O (grey)	Beta
■	*■*	■ (grey)	B (Unstandardized Coefficient)
⌘	*⌘*	⌘ (grey)	Partial Correlation
●	*●*	● (grey)	F-Ratio
➔ (down)	*➔ (down)*	➔ (down, grey)	Effect Size

[a]This paper is one of a series of Meta-Analyses and Systematic Reviews assessing the relationship between child care quality and children's outcomes; therefore, superscript letters below are in reference to various large databases that samples in these papers were drawn from. These letters have been kept consistent across the series for our readers.
[b]Samples within papers are described in more detail in S3.
[c]Acronyms for child outcomes are listed in S4.
[C]Bermuda Preschool Study (1980); [D]Cost, Quality and Outcomes Study (CQO, 1993-1994); [E]Effective Preschool and Primary Education Study (EPPE, 1997-1998); [F]Georgia Early Childhood Study (GECS, 2002); [R]Northeastern United States sample (Moller and colleagues, 2008; Year NR); [Z]Colorado QRIS.

The Relationship between the Early Childhood Environment Rating Scale and its Revised Form and Child Outcomes: a Systematic Review and Meta-Analysis

Supplemental Information 5
Systematic Review Results: All Outcomes

Table B. ECERS/ECERS-R Predicting all Cognitive Outcomes

**ECERS/ECERS-R Measurement Level**		**STUDY[a,b]**	**COGNITIVE[c]**																				
			Academic Achievement	BAS II - Pattern Construction	BAS II - Picture Similarities	Backward Digit Span	Bayley - Mental Development	CBI - Intelligence	Cognitive Composite	COR- Cognitive	DIAL-R - Concepts	Forward Digit Span	Identifying Colors[d]	K-ABC Achievement	K-ABC Mental Processing	PSPCSA - Competence	Preschool Inventory	School Readiness	Skills Mastery	Slosson Intelligence Test	FACES - Social Awareness	WPPSI-III Matrix Reasoning	WPPSI-III Similarities
**ECERS**	**Total Score**	Bryant 1994[12]- NonSuspect Homes												■	■		■						
		Bryant 1994[12]– Suspect Homes												■	■		■						
		Burchinal, Peisner-2000[15] – NC HS												★									
		Dunn 1993[26,S]						★ ■									⌘						
		Epstein 1993[29]									★												
		Kontos 1991[43]						★ ○												★ ○			
		Lyon 1995[46]														●							
		McCartney 1982[49,C]						★ ■															
	**Total Score (Parents and Staff Subscale not included)**	Burchinal, Roberts 2000[16,T]					★																
		Chin-Quee 1994[21] – Primary 1,2	★																				

The Relationship between the Early Childhood Environment Rating Scale and its Revised Form and Child Outcomes: a Systematic Review and Meta-Analysis

		Chin-Quee 1994[21] – Primary 3,4	★ ○																				
		Phillips 1987[62,C]						🡇 🡇															
		Seppanen 1993[68]															⌘						
ECERS-R	Total Score	Aboud 2006[1,V]																★				★	★
		Henry 2003[33,F]																	■				
		Moller…Hightower 2008[52,R]								■													
		Moller…Friedman 2008[53,R]								■													
		Peisner-Feinberg 2013[61]																			■		
		Sylva 2006[69,E]		■	■																		
		Weiland 2013[70]				■ ■ ■						■ ■ ■											
	ECERS-R – Total Score (Parents and Staff Subscale not included)	Le 2015[45,Z]							■ ■ ■ ■														
		Peisner-Feinberg 2006[57,YA, YC]											■								■		
		Peisner-Feinberg 2007[58,YC]											■								■		
		Peisner-Feinberg 2008[60] - NC 07/08																			❖		
		Peisner-Feinberg 2008[59] – NC 03/07 [YB, YC]											❖								❖		
		Zellman 2008[73,Z]						■															
ECERS Subscales	Creative Activities	Epstein 1993[29]									★												
	Fine and Gross Motor Activities	Epstein 1993[29]									★												
	Furnishings and Display	Epstein 1993[29]									★												

	**Language-Reasoning**	Epstein 1993[29]									★ (grey)												
		Dunn 1994[27,S]						★ ○									★ (grey)						
	**Personal Care Routines**	Epstein 1993[29]									★ (grey)												
	**Social Development**	Epstein 1993[29]									★ (grey)												
**ECERS-R Subscales**	**Activities**	Sylva 2006[69,E]		■	■																		
	**Interactions**	Sylva 2006[69,E]		■	■																		
	**Language-Reasoning**	Sylva 2006[69,E]		■	■																		
		Zill 2006[75,K]											■								■		
	**Parents and Staff**	Sylva 2006[69,E]		■	■																		
	**Personal Care Routines**	Sylva 2006[69,E]		■	■																		
	**Program Structure**	Sylva 2006[69,E]		■	■																		
	**Space and Furnishings**	Sylva 2006[69,E]		■	■																		
**ECERS Factors**	**Developmentally Appropriate Activities**	Dunn 1994[27,S]						★ ○									★ (grey)						
		Lyon 1995[46]														●							
	**Preschool Appropriate Caregiving**	Lyon 1995[46]														●							
		Whitebrook 1989[72,O]														⌘							
**ECERS-R Factors**	**Provisions for Learning**	Early 2006[28,A]											★ (grey)										
		Weiland 2013[70]				■■ ■						■ ■ ■											
	**Teaching & Interactions**	Early 2006[28,A]											★										
		Weiland 2013[70]				■■ ■						■ ■ ■											

Legend for Table

**Significant and Positive**	**Significant and Negative**	**Non-Significant**	**Statistic**

The Relationship between the Early Childhood Environment Rating Scale and its Revised Form and Child Outcomes: a Systematic Review and Meta-Analysis

★	★	★	r - Zero Order Pearson's Correlation
○	○	○	Beta
■	■	■	B (Unstandardized Coefficient)
❖	❖	❖	T-Test
⌘	⌘	⌘	Partial Correlation
●	●	●	F-Ratio
➔	➔	➔	Effect Size

[a]This paper is one of a series of Meta-Analyses and Systematic Reviews assessing the relationship between child care quality and children's outcomes; therefore, superscript letters below are in reference to various large databases that samples in these papers were drawn from. These letters have been kept consistent across the series for our readers.

[b]Samples within papers are described in more detail in S3.

[c]Acronyms for child outcomes are listed in S4.

[d]Identifying Colors (also referred to as Color Knowledge, Color Naming, Color Naming Task).

[A]National Center for Early Development and Learning Dataset (NCEDL, 2002, 2004); [C]Bermuda Preschool Study (1980); [E]Effective Preschool and Primary Education Study (EPPE, 1997-1998); [F]Georgia Early Childhood Study (GECS, 2002); [K]Head Start Family and Children Experiences Survey (FACES,2000) Cohort; [O]National Child Care Staffing Study (NCCSS, 1988); [R]Northeastern United States sample (Moller and colleagues, 2008; Year NR); [S]8-County Region of North-Central Indiana (Year NR); [T]Otitis Media Study (Year NR); [V]Five Rural Districts of Bangladesh (2006-2008); [YA]More is Four North Carolina Study (2002-2003) Cohort; [YB]More is Four North Carolina Study (2003-2004) Cohort; [YC]More is Four North Carolina Study (2005-2006) Cohort; [Z]Colorado QRIS.

The Relationship between the Early Childhood Environment Rating Scale and its Revised Form and Child Outcomes: a Systematic Review and Meta-Analysis

Supplemental Information 5
Systematic Review Results: All Outcomes

Table C. ECERS/ECERS-R Predicting all Physical and Math Outcomes

**ECERS/ECERS-R Measurement Level**		**STUDY**[a,b]	**MATH**[c]									**PHYSICAL**				
			BAS II - Early Number Concepts	COR - Logic & Math	Counting Task[d]	ECLS-B Math	Identifying Numbers[e]	K-ABC Arithmetic	Math (Grade 1)	WJ - Applied Problems	WPPSI- Math	COR - Motor	COR - Music & Movement	Design Copying	DIAL-R - Motor	PSPCSA - Physical Competence
**ECERS**	**Total Score**	Burchinal, Peisner 2000[15] - CQO[D]								★						
		Burchinal, Peisner 2000[15] - NC PS								★ (grey)						
		Burchinal 2011[19] - CQO[D]								■■⌘ (grey)						
		Epstein 1993[29]		★ (grey)									★ (grey)		★ (grey)	
		Lyon 1995[46]														●● (grey)
	**Total Score (Parents and Staff Subscale not included)**	Bryant 2003[13] - Whole Sample			■					■						
		Peisner-Feinberg 1997[55,D]								★						
	**Mean of 5 Items**	Peisner-Feinberg 1999[56,D]								★↓ (arrow grey)						
**ECERS - R**	**Total Score**	Abreu-Lima 2013[3]			★ ■ (grey)		★ ■ (grey)				★ ■ (grey)					
		Anders 2012[8]						■ ■ (grey)								
		Barnett 2007[11] – Whole Sample								■ (grey)						
		Barnett 2007[11] - Spanish								■ (grey)						

		Burchinal 2011[19] – NCEDL[A]								■■⌘						
		Burchinal 2011[19] – FACES 1997[J]								▰■⌘						
		Burchinal 2011[19] – FACES 2000[K]								■■⌘						
		Henry 2003[33,F]								■						
		Henry 2005[34,F]								▰						
		Jeon 2010[41]								★○						
		Moller...Friedman 2008[53,R]										■				
		Moller...Hightower 2008[52,R]										■				
		Peisner-Feinberg 2013[61]			■					■						
		Sabol 2013[65,A]								★○						
		Sylva 2006[69,E]	■													
	**Total Score (Parents and Staff Subscale not included)**	Aboud 2011[2]							○							
		Dang 2011[23] - ECLS-B 05/06[N]				■										
		Dang 2011[23] – NCEDL[A]								■						
		Dang 2011[23] - EHS 01/03[H]								■						
		Gordon 2013[32,N]				○										
		Hindman 2010[37,J]								■						
		Keys 2013[42] – ECLS-B 05/06[N]				■										
		Keys 2013[42] - NCEDL[A]								■						
		Keys 2013[42] - EHS 01/03[H]								■						
		Le 2015[45,Z]								■■■■						
		Mashburn, Pianta 2008[48,A]								■						
		Peisner-Feinberg 2006[57,YA, YC]			■					■						
		Peisner-Feinberg 2007[58,YC]			■					■						
		Peisner-Feinberg 2008[60]- NC 07/08			❖					❖						

		Peisner-Feinberg 2008[59]- NC 03/07[YB, YC]			❖					❖						
		Reid 2013[64,A]								○						
		Sabol 2014[66,N]				■■■■■■■■■■■ ■■■■■■■■■■■ ■■■■■■■■■■■ ■★										
		Zellman 2008[73,Z]								■						
ECERS Subscales	**Creative Activities**	Epstein 1993[29]		★									★		★	
	**Fine and Gross Motor Skills**	Epstein 1993[29]		★									★		★	
	**Furnishings and Display**	Epstein 1993[29]		★									★		★	
	**Interactions (Author Created)**	Burchinal 2011[19] – CQO[D]								⌘						
	**Language-Reasoning**	Burchinal 2011[19] – CQO[D]								⌘						
		Epstein 1993[29]		★									★		★	
	**Personal Care Routines**	Epstein 1993[29]		★									★		★	
	**Social Development**	Epstein 1993[29]		★									★		★	
ECERS-R Subscales	**Activities**	Jeon 2010[41]								★○						
		Sylva 2006[69,E]	■													
	**Interactions**	Burchinal 2011[19] – NCEDL[A]								⌘						
		Burchinal 2011[19] – FACES 1997[J]								⌘						
		Burchinal 2011[19] – FACES 2000[K]								⌘						
		Jeon 2010[41]								★○						
		Sylva 2006[69,E]	■													
	**Language-**	Burchinal 2011[19] – NCEDL[A]								⌘						

	**Reasoning**	Burchinal 2011[19] – FACES 1997[J]								⌘						
		Burchinal 2011[19] – FACES 2000[K]								⌘						
		Jeon 2010[41]								★○						
		Sylva 2006[69,E]	■													
		Zill 2006[75,K]			■					■				■		
	**Parents and Staff**	Sylva 2006[69,E]	■													
	**Personal Care Routines**	Sylva 2006[69,E]	■													
	**Program Structure**	Jeon 2010[41]								★○						
		Sylva 2006[69,E]	■													
	**Space and Furnishings**	Sylva 2006[69,E]	■													
**ECERS Factors**	**Developmentally Appropriate Activities**	Lyon 1995[46]														●●
	**Preschool Appropriate Caregiving**	Lyon 1995[46]														●●
**ECERS-R Factors**	**Provisions for Learning**	Auger 2014[10,U]								■						
		Dotterer 2012[25,A]					■			■						
		Early 2006[28,A]					★			**★**						
		Howes 2008[39,A]								■**★**						
		West 2010[71,B]				○				○**○**						
	**Teaching & Interactions**	Aikens 2010[6,B]				○○ ○**○**				○○						
		Aikens 2012[6,M]				■■■ ■■				■■■■■						
		Auger 2014[10,U]								■						
		Burchinal 2008[18,A]								**★**						
		Dotterer 2012[25,A]					■			■						
		Early 2006[28,A]					★			**★**						

		Howes 2008[39,A]								■★						
		Sabol 2013[65,A]								**O**						
		West 2010[71,B]				○				**OO**						

Legend for Table

**Significant and Positive**	**Significant and Negative**	**Non-Significant**	**Statistic**
★	★	★	r - Zero Order Pearson's Correlation
**O**	*O*	○	Beta
■	■	■	B (Unstandardized Coefficient)
❖	❖	❖	T-Test
⌘	⌘	⌘	Partial Correlation
●	●	●	F-Ratio
🡻	🡻	🡻	Effect Size

[a]This paper is one of a series of Meta-Analyses and Systematic Reviews assessing the relationship between child care quality and children's outcomes; therefore, superscript letters below are in reference to various large databases that samples in these papers were drawn from. These letters have been kept consistent across the series for our readers.
[b]Samples within papers are described in more detail in S3.
[c]Acronyms for child outcomes are listed in S4.
[d]Counting Task (also referred to as One to One Counting).
[e]Identifying Numbers (also referred to as Naming Numbers).
[A]National Center for Early Development and Learning Dataset (NCEDL, 2002, 2004); [B]Head Start Family and Children Experiences Survey (FACES, 2006) Cohort; [D]Cost, Quality and Outcomes Study (CQO, 1993-1994); [E]Effective Preschool and Primary Education Study (EPPE, 1997-1998); [F]Georgia Early Childhood Study (GECS, 2002); [H]Early Head Start (EHS, 2001-2003 Cohort); [J]Head Start Family and Children Experiences Survey (FACES, 1997) Cohort; [K]Head Start Family and Children Experiences Survey (FACES, 2000) Cohort; [M]Head Start Family and Children Experiences Survey (FACES, 2009) Cohort; [N]Early Childhood Longitudinal Study (ECLS-B, 2001-2006, Birth Cohort); [R]Northeastern United States sample (Moller and colleagues, 2008; Year NR);[U] Preschool Curriculum Evaluation Research (PCER, 1999-2003); **[YA]**More is Four North Carolina Study (2002-2003) Cohort; **[YB]**More is Four North Carolina Study (2003-2004) Cohort; **[YC]**More is Four North Carolina Study (2005-2006) Cohort; [Z] Colorado QRIS.

The Relationship between the Early Childhood Environment Rating Scale and its Revised Form and Child Outcomes: a Systematic Review and Meta-Analysis

Supplemental Information 5
Systematic Review Results: All Outcomes

Table D. ECERS/ECERS-R Predicting all Positive Behavior Outcomes A to P

ECERS/ECERS-R Measurement Level		STUDY[a,b]	Positive Behavior[c]																			
			Adult Orientation	ASBI - Comply	ASBI - Cooperation and Conformity	ASBI - Express	ASBI - Peer Sociability	Attitudes/Perceptions	BPI - Social Skills	CBI - Considerateness	CBI - Sociability	COR - Social Relations	Cooperative Behavior	ECLS-B - Attention and Concentration	ECLS-B- - Emotional and Behavioral Regulation	ECLS-B - Prosocial	Entwistle Scale of Personal Maturity	Learning Self-Efficacy	Pencil Tap Task	PSPCSA - Cognitive Competence	PSPCSA - Maternal Acceptance	PSPCSA - Peer Acceptance
ECERS	Total Score	Bryant 1994[12] - NonSuspect Homes		■		■																
		Bryant 1994[12] - Suspect Homes		■		■																
		Dunn 1993[26,S]									★											
		Epstein 1993[29]										★										
		Herrera 2005[35]																				
		Kontos 1991[43]									○★											
		Lyon 1995[46]															●			●	●	●●
		McCartney 1982[49,C]	■ ★							■★	■★											

The Relationship between the Early Childhood Environment Rating Scale and its Revised Form and Child Outcomes: a Systematic Review and Meta-Analysis

	**ECERS - Total Score (Parents and Staff Subscale not included)**	Peisner-Feinberg 1997[55,D]						★			★											
		Phillips 1987[62,C]								⬇⬇	⬇⬇											
	**Mean of 5 Items**	Peisner-Feinberg 1999[56,D]									★⬇											
**ECERS - R**	**Total Score**	Abreu-Lima 2013[3]								★■	★■											
		Burchinal 2011[19] - FACES 1997[J]							■⌘■													
		Burchinal 2011[19] - FACES 2000[K]							■⌘■													
		Hestenes 2015[36]																■				
		Moller… Hightower 2008[52,R]										■										
		Moller… Friedman 2008[53,R]										■										
		Sylva 2006[69,E]			■		■															
		Weiland 2013[70]																	■■ ■			
	**ECERS - R Total Score (Parents and Staff Subscale not included)**	Gordon 2013[32,N]												○	○	○						
		Keys 2013[42] - ECLS-B 2005-2006[N]														■						

		Sabol 2014[66,N]														■■ ■■ ■■ ■■ ★						
		Zellman 2008[73,Z]								■												
**ECERS Subscales**	**Creative Activities**	Epstein 1993[29]										★										
	**Fine and Gross Motor Activities**	Epstein 1993[29]										★										
	**Furnishings**	Epstein 1993[29]										★										
	**Language-Reasoning**	Epstein 1993[29]										★										
	**Personal Care Routines**	Epstein 1993[29]										★										
	**Social Development**	Epstein 1993[29]										★										
**ECERS-R Subscales**	**Activities**	Sylva 2006[69,E]			■		■															
	**Interactions**	Burchinal 2011[19] - FACES 1997[J]							⌘													
		Burchinal 2011[19]- FACES 2000[K]							⌘													
		Sylva 2006[69,E]			■		■															
	**Language-Reasoning**	Sylva 2006[69,E]			■		■															
		Zill 2003[74,K]											■									
	**Parents and Staff**	Sylva 2006[69,E]			■		■															
	**Personal Care Routines**	Sylva 2006[69,E]			■		■															
	**Program**	Sylva			■		■															

	**Structure**	2006[69,E]																				
	**Space and Furnishings**	Sylva 2006[69,E]			■		■															
**ECERS Factors**	**Developmentally Appropriate Activities**	Lyon 1995[46]															●			●	●	●●
	**Preschool Appropriate Caregiving**	Lyon 1995[46]															●			●	●	●●
		Whitebook 1989[72,O]															⌘			⌘●		⌘⌘
**ECERS-R Factors**	**Teaching and Interactions**	Weiland 2013[70]																	■■■			
	**Provisions for Learning**	Weiland 2013[70]																	■■■			

Legend for Table

**Significant & Positive**	**Significant & Negative**	**Non-Significant**	**Statistic**
★	★	★	r - Zero Order Pearson's Correlation
**O**	***O***	O	Beta
■	■	■	B (Unstandardized Coefficient)
⌘	⌘	⌘	Partial Correlation
●	●	●	F-Ratio
➔	➔	➔	Effect Size

[a]This paper is one of a series of Meta-Analyses and Systematic Reviews assessing the relationship between child care quality and children's outcomes; therefore, superscript letters below are in reference to various large databases that samples in these papers were drawn from. These letters have been kept consistent across the series for our readers.
[b]Samples within papers are described in more detail in S3.
[c]Acronyms for child outcomes are listed in S4.
[A]National Center for Early Development and Learning Dataset (NCEDL, 2002, 2004); [C]Bermuda Preschool Study (1980); [D]Cost, Quality and Outcomes Study (CQO, 1993-1994); [E]Effective Preschool and Primary Education Study (EPPE, 1997-1998); [J]Head Start Family and Children Experiences Survey (FACES, 1997) Cohort; [K]Head Start Family and Children Experiences Survey (FACES, 2000) Cohort; [N]Early Childhood Longitudinal Study (ECLS-B, 2001-2006, Birth Cohort);); [O]National Child Care Staffing Study (NCCSS, 1988); [R]Northeastern United States sample (Moller and colleagues, 2008; Year NR); [S]Eight-County Region of North-Central Indiana (Year NR); [Z]Colorado QRIS.

The Relationship between the Early Childhood Environment Rating Scale and its Revised Form and Child Outcomes: a Systematic Review and Meta-Analysis

Supplemental Information 5
Systematic Review Results: All Outcomes

Table E. ECERS/ECERS-R Predicting all Positive Behavior Outcomes S to Z

ECERS/ECERS-R Measurement Level		STUDY[a,b]	Positive Behavior									
			Social Competence (author created)	SSBPS - Social Competence	SSRS/SSIS - Social Skills	Student-Teacher Relationship Scale	TCRS - Assertive Social Skills	TCRS - Behavior Control	TCRS - Decreased Risk Status	TCRS - Peer Sociability	TCRS - Percent Not at Risk	TCRS - Social Competence
**ECERS**	**Total Score**	Burchinal 2006[17,T]			★							
		Herrera 2005[35]	★									
	**Total Score (Parents and Staff Subscale not included)**	Bryant 2003[13] – Whole Sample			■							
		Chin-Quee 1994[21] – Primary 1,2	★○									
		Chin-Quee 1994[21] – Primary 3,4	★									
**ECERS - R**	**Total Score**	Burchinal 2011[19] – NCEDL[A]										■⌘■
		Jeon 2010[41]			○★	○★						
		Hestenes 2015[36]			■							
		Montes 2005[54]					●	●	●■	●	●	
		Peisner-Feinberg 2013[61]			■							
		Sabol 2013[65,A]										○★
	**Total Score (Parents and Staff Subscale not included)**	Keys 2013[42] – NCEDL[A]										■
		Le 2015[45,Z]	■									

		Mashburn, Pianta 2008[48,A]										■
		Peisner-Feinberg 2006[57,YA, YC]			■							
		Peisner-Feinberg 2007[58,YC]			■							
		Peisner-Feinberg 2008[60] - NC 07/08			❖							
		Peisner-Feinberg 2008[59] - NC 03/07[YB,YC]			**❖**							
		Reid 2013[64,A]										○
**ECERS-R Subscale**	**Activities**	Jeon 2010[41]			**○★**	○★						
	**Interactions**	Jeon 2010[41]			○★	○★						
		Burchinal 2011[19] – NCEDL[A]										⌘
	**Language-Reasoning**	Jeon 2010[41]			○★	○★						
		Zill 2006[75,K]			■■							
	**Program Structure**	Jeon 2010[41]			○**★**	○★						
**ECERS-R Factors**	**Provisions for Learning**	Howes 2008[39,A]		■★								
		West 2010[71,B]			○							
	**Teaching & Interactions**	Aikens 2010[4,B]			○○							
		Burchinal 2008[18,A]										★
		Howes 2008[39,A]		■★								
		Sabol 2013[65,A]										○
		West 2010[71,B]			○							

Legend for Table

**Significant and Positive**	**Significant and Negative**	**Non-Significant**	**Statistic**
**★**	***★***	★	r - Zero Order Pearson's Correlation
**○**	***○***	○	Beta
**■**	***■***	■	B (Unstandardized Coefficient)
**❖**	***❖***	❖	T-Test
**⌘**	***⌘***	⌘	Partial Correlation
**●**	***●***	●	F-Ratio

[a]This paper is one of a series of Meta-Analyses and Systematic Reviews assessing the relationship between child care quality and children's outcomes; therefore, superscript letters below are in reference to various large databases that samples in these papers were drawn from. These letters have been kept consistent across the series for our readers.
[b]Samples within papers are described in more detail in S3.

The Relationship between the Early Childhood Environment Rating Scale and its Revised Form and Child Outcomes: a Systematic Review and Meta-Analysis

[c]Acronyms for child outcomes are listed in S4.
[A]National Center for Early Development and Learning Dataset (NCEDL, 2002, 2004); [B]Head Start Family and Children Experiences Survey (FACES, 2006) Cohort; [K]Head Start Family and Children Experiences Survey (FACES, 2000) Cohort; [T]Otitis Media Study (Year NR); **[YA]**More is Four North Carolina Study (2002-2003) Cohort; **[YB]**More is Four North Carolina Study (2003-2004) Cohort; **[YC]**More is Four North Carolina Study (2005-2006) Cohort; **[Z]**Colorado QRIS;
[Z]Colorado QRIS.

The Relationship between the Early Childhood Environment Rating Scale and its Revised Form and Child Outcomes: a Systematic Review and Meta-Analysis

Supplemental Information 5
Systematic Review Results: All Outcomes

Table F. ECERS Predicting all Problem Behavior Outcomes

ECERS Measurement Level		STUDY[a,b]	Problem Behavior[c]																											
			ASBI - Antisocial/Worried	ASBI - Disruptive	Problem Behaviors (Composite)	BPI - Problem Behavior	CBCL - Aggressive	CBCL - Problem Behavior	CBI - Apathy	CBI - Behavior Problems	CBI - Hostility	ECLS-B - Externalizing Problems	FACES - Aggressive Behavior	FACES - Hyperactive Behavior	FACES - Withdrawn Behaviors	PBQ - Aggressiveness/Hostility	PBQ - Anxiety	PBQ - Hyperactivity/Distractibility	PBQ - Total Maladjustment	PBS – Hyperactive Behavior	SSIS – Externalizing Behavior	SSIS – Internalizing Behavior	SSIS/SSRS - Problem Behavior	SSPBS – Behavior Problems	TCRS - Behavior Problems	TCRS – Change in Risk	TCRS – Increased Risk	TCRS – Multiple Risk	TCRS – Social Emotional Risk	TRF - Problem Behavior
ECERS	Total Score	Bryant 1994[12] – NonSuspect Homes		■																										
		Bryant 1994[12] – Suspect Homes		■																										
		Burchinal, Peisner 2000[15] – CQO[D]								★																				
		Burchinal, Peisner 2000[15] – NC HS		★																										
		Burchinal, Peisner 2000[15] – NC PS		★																										
		Burchinal 2011[19] – CQO[D]								■<br>■<br>⌘																				
		Dunn 1993[26,S]																★												
		Kontos 1991[43]																○<br>★												
		McCartney 1982[49,C]														■<br>★	■<br>★	■<br>★	■<br>★											

	**ECERS - Total Score (Parents and Staff Subscale not included)**	Bryant 2003[13] – Whole Sample																				■								
		Peisner-Feinberg 1997[55,D]								★																				
		Phillips 1987[62,C]														↓ ↓	↓ ↓	↓ ↓												
	**Mean of 5 Items**	Peisner-Feinberg 1999[56,D]								★ ↓																				
**ECERS - R**	**Total Score**	Burchinal 2011[19] – NCEDL[A]																						■ ■ ⌘						
		Burchinal 2011[19] – FACES 1997[J]				▰ ■ ⌘																								
		Burchinal 2011[19] – FACES 2000[K]				■ ■ ⌘																								
		Clawson 2008[22]						★																						★
		Hestenes 2015[36]																			■	■								
		Montes 2005[54]																								●	▰ ●	●	●	
		Peisner-Feinberg 2013[61]																					■							
		Sabol 2013[65,A]																							★ ○					
		Sylva 2006[69,E]	■																											
	**ECERS-R – Total Score (Parents and Staff Subscale not included)**	Keys 2013[42] – ECLS-B 05/06[N]										■																		
		Keys 2013[42] – NCEDL[A]																							■					
		Keys 2013[42] – EHS 01/03[H]											■																	
		Mashburn, Pianta 2008[48,A]																							■					
		Peisner-Feinberg 2006[57,YA,YC]																					■							
		Peisner-Feinberg 2007[58,YC]																					■							
		Peisner-Feinberg 2008[60]- NC 07/08																					❖							

		Peisner-Feinberg 2008[59] NC 03/07[YB,YC]																				❖								
		Sabol 2014[66,N]									■<br>■<br>■<br>■<br>■<br>■<br>■<br>■<br>★																				
		Zellman 2008[73,Z]							■		■																				
ECERS Subscales	ECERS-Interactions (Author Created)	Burchinal 2011[19] – CQO[D]								⌘																					
ECERS-R Subscales	Activities	Sylva 2006[69,E]	■																												
	Interactions	Burchinal 2011[19] – NCEDL[A]																							⌘						
		Burchinal 2011[19] – FACES 1997[J]				⌘																									
		Burchinal 2011[19] – FACES 2000[K]				⌘																									
		Sylva 2006[69,E]	■																												
	Language-Reasoning	Sylva 2006[69,E]	■																												
		Zill 2003[74,K]																		■											
		Zill 2006[75,K]											■<br>■	■<br>■	■<br>■																
	Parents and Staff	Sylva 2006[69,E]	■																												
	Personal Care Routines	Sylva 2006[69,E]	■																												
	Program Structure	Sylva 2006[69,E]	■																												

	**Space and Furnishings**	Sylva 2006[69,E]	■																											
**ECERS-R Factors**	**Provisions for Learning**	Howes 2008[39,A]																				■ ★						
		West 2010[71,B]																			○							
	**Teaching & Interactions**	Aikens 2010[4,B]			○ ○																							
		Burchinal 2008[18,A]																					★					
		Howes 2008[39,A]																				■ ★						
		Sabol 2013[65,A]																					○					
		West 2010[71,B]																			○							

Legend for Table

**Significant and Positive**	**Significant and Negative**	**Nonsignificant**	**Statistic**
★	★	★	r - Zero Order Pearson's Correlation
○	○	○	Beta
■	■	■	B (Unstandardized Coefficient)
❖	❖	❖	T-Test
⌘	⌘	⌘	Partial Correlation
●	●	●	F-Ratio
🡻	🡻	🡻	Effect Size

[a]This paper is one of a series of Meta-Analyses and Systematic Reviews assessing the relationship between child care quality and children's outcomes; therefore, superscript letters below are in reference to various large databases that samples in these papers were drawn from. These letters have been kept consistent across the series for our readers.

[b]Samples within papers are described in more detail in S3.

[c]Acronyms for child outcomes are listed in S4.

[A]National Center for Early Development and Learning Dataset (NCEDL, 2002, 2004); [B]Head Start Family and Children Experiences Survey (FACES, 2006) Cohort; [C]Bermuda Preschool Study (1980); [D]Cost, Quality and Outcomes Study (CQO, 1993-1994); [E]Effective Preschool and Primary Education Study (EPPE, 1997-1998); [H]Early Head Start (EHS, 2001-2003 Cohort); [J]Head Start Family and Children Experiences Survey (FACES, 1997) Cohort; [K]Head Start Family and Children Experiences Survey (FACES, 2000) Cohort; [N]Early Childhood Longitudinal Study (ECLS-B, 2001-2006, Birth Cohort); [S]8-County Region of North-Central Indiana (Year NR); [YA]More is Four North Carolina Study (2002-2003) Cohort; [YB]More is Four North Carolina Study (2003-2004) Cohort; [YC]More is Four North Carolina Study (2005-2006) Cohort; [Z] Colorado QRIS.

The Relationship between the Early Childhood Environment Rating Scale and its Revised Form and Child Outcomes: a Systematic Review and Meta-Analysis

Supplemental Information 5
Systematic Review Results: All Outcomes

Table G. ECERS Predicting all Language Outcomes A to L

ECERS	Measurement Level	STUDY[a,b]	LANGUAGE[c]: ARS - Language Literacy	Adaptive Language Inventory	Book Knowledge	BAS-II - Language	BAS - II - Verbal Fluency	BAS - II – Word Reading	CAP - Emergent Literacy	COR - Language	Communication Task	DIAL-R - Language	ECLS-K – Language Literacy	Expressive One Word Picture Vocabulary Test	Griffiths -Language (hearing and speech)	Identifying Letters[d]	Language Skills (Composite)
ECERS	Total Score	Burchinal 2006[17]															★
		Epstein 1993[29]								★ (grey)		★					
		Goelman 1988[31]												★ (grey)			
		Kontos 1991[43]		○ (grey)<br>★ (grey)													
		Kwan 1998[44]					↓	↓ (grey)									
		Lyon 1995[46]		● (grey)													
		McCartney 1982[49,C]		■ (grey)<br>★													
	Total Score (Parents and Staff Subscale not included)	Bryant 2003[13] - Whole Sample														■ (grey)	
		McCartney 1984[50,E]		○<br>★							○<br>★						

The Relationship between the Early Childhood Environment Rating Scale and its Revised Form and Child Outcomes: a Systematic Review and Meta-Analysis

ECERS-R	Total Score	Abreu-Lima 2013[3]														■ (grey)<br>★ (grey)	
		Barnett 2007[11] - Whole Sample														■ (grey)	
		Barnett 2007[11] - Spanish Sample														■	
		Peisner-Feinberg 2013[61]														■ (grey)	
		Sabol 2013[65,A]														○ (grey)<br>★ (grey)	
		Sylva 2006[69,E]				■ (grey)											
	ECERS-R - Total Score (Parents and Staff Subscale not included)	Dang 2011[23] - ECLS-B 05/06[N]	■														
		Gordon 2013[32,N]	○														
		Keys 2013[42] - ECLS-B 05/06[N]	■														
		Mashburn 2008[47,F]															■ (grey)
		Mashburn, Pianta 2008[48,A]														■ (grey)	
		Peisner-Feinberg 2006[57, YA, YC]														■ (grey)	
		Peisner-Feinberg 2007[58,YC]														▰	
		Peisner-Feinberg 2008[59] - NC 03/07[YB, YC]														❖	
		Pinto 2013[63] - (Portuguese version)													★ ■ (grey)		
		Sabol 2014[66,N]	■■■■■■ (grey)<br>■■■■ (grey)<br>■■■■■■ (grey)<br>■■■■ (grey)<br>■■■■■■ (grey)<br>■■■■ (grey)<br>■■■■ (grey)<br>★ (grey)														
ECERS Subscales	Adult Needs	Kwan 1998[44]					↓	↓									
	Creative Activities	Epstein 1993[29]								★ (grey)		★					
		Kwan 1998[44]					↓	↓ (grey)									

The Relationship between the Early Childhood Environment Rating Scale and its Revised Form and Child Outcomes: a Systematic Review and Meta-Analysis

	Fine and Gross Motor Skills	Epstein 1993[29]								★		★					
		Kwan 1998[44]					↓ (grey)	↓ (grey)									
	Furnishings and Display	Epstein 1993[29]								★ (grey)		★					
		Kwan 1998[44]					↓	↓ (grey)									
	Language-Reasoning	Epstein 1993[29]								★ (grey)		★					
		Dickinson 2001[24]							★ (grey)								
		Kwan 1998[44]					↓	↓ (grey)									
	Personal Care Routines	Epstein 1993[29]								★ (grey)		★ (grey)					
		Kwan 1998[44]					↓	↓									
	Social Development	Epstein 1993[29]								★ (grey)		★					
		Kwan 1998[44]					↓	↓ (grey)									
ECERS-R Subscales	Activities	Sylva 2006[69,E]				■ (grey)											
	Interactions	Sylva 2006[69,E]				■ (grey)											
	Language-Reasoning	Sylva 2006[69,E]				■ (grey)											
	Parents and Staff	Sylva 2006[69,E]				■ (grey)											
	Personal Care Routines	Sylva 2006[69,E]				■ (grey)											
	Program Structure	Sylva 2006[69,E]				■ (grey)											
	Space and Furnishings	Sylva 2006[69,E]				■ (grey)											
ECERS Factors	Developmentally Appropriate Activities	Lyon 1995[46]		● (grey)													
	Preschool Appropriate Caregiving	Lyon 1995[46]		● (grey)													
		Whitebook 1989[72,O]		⌘													
ECERS-R Factors	Provisions for Learning	Dotterer 2012[25,A]	■ (grey)													■ (grey)	
		Early 2006[28,A]														★ (grey)	

The Relationship between the Early Childhood Environment Rating Scale and its Revised Form and Child Outcomes: a Systematic Review and Meta-Analysis

		Howes 2008[39,A]	■★													■<br>★	
	**Teaching & Interactions**	Aikens 2012[6,M]												■■<br>■<br>■■			
		Burchinal 2008[18,A]	★														
		Dotterer 2012[25,A]	■													■	
		Early 2006[28,A]														★	
		Howes 2008[39,A]	■★													■<br>★	
		Sabol 2013[65,A]														○	

Legend for Table

Significant and Positive	Significant and Negative	Non-Significant	Statistic
★	★	★	r - Zero Order Pearson's Correlation
○	○	○	Beta
■	■	■	B (Unstandardized Coefficient)
❖	❖	❖	T-Test
⌘	⌘	⌘	Partial Correlation
●	●	●	F-Ratio
⬇	⬇	⬇	Effect Size

[a]This paper is one of a series of Meta-Analyses and Systematic Reviews assessing the relationship between child care quality and children's outcomes; therefore, superscript letters below are in reference to various large databases that samples in these papers were drawn from. These letters have been kept consistent across the series for our readers.
[b]Samples within papers are described in more detail in S3.
[c]Acronyms for child outcomes are listed in S4.
[d]Identifying Letters (also referred to as Alphabet Recognition Test; Letter Identification, Letter Knowledge, Letter-Naming Test, Naming Letters, Cross-Linguistic Assessment of Foundation Level-Letter Identification).
[A]National Center for Early Development and Learning Dataset (NCEDL, 2002, 2004); [C]Bermuda Preschool Study (1980); [E]Effective Preschool and Primary Education Study (EPPE, 1997-1998); [F]Georgia Early Childhood Study (GECS, 2002); [M]Head Start Family and Children Experiences Survey (FACES, 2009) Cohort; [N]Early Childhood Longitudinal Study (ECLS-B, 2001-2006, Birth Cohort); [O]National Child Care Staffing Study (NCCSS, 1988); [YA]More is Four North Carolina Study (2002-2003) Cohort; [YB]More is Four North Carolina Study (2003-2004) Cohort; [YC]More is Four North Carolina Study (2005-2006) Cohort.

The Relationship between the Early Childhood Environment Rating Scale and its Revised Form and Child Outcomes: a Systematic Review and Meta-Analysis

Supplemental Information 5
Systematic Review Results: All Outcomes

Table H. ECERS Predicting all Language Outcomes M to S

**ECERS Measurement Level**		**STUDY**[a,b]	**LANGUAGE**[c]																
			MacArthur Story Stem Battery	OWLS - Oral Expression Scale	OWLS - Total Scale	PAT – Phonological Awareness	PPVT - Vocabulary	Phoneme Deletion	Pre-Reading	PreLAS 2000 – Language	PreLAS 2000	Preschool Language Assessment	Reading (Grade 1)	Rhyme Recognition	SHELL-K - Formal Definitions	SHELL-K - Narrative Production	SICD - Expressive Communication Age	SICD - Receptive Communication Age
**ECERS**	**Total Score**	Burchinal, Nelson 2000[14,D]					■⌘⌘⌘ ⌘⌘⌘ ★★											
		Burchinal, Peisner 2000[15] – CQO[D]					★											
		Burchinal, Peisner 2000[15] – NC PS					★											
		Burchinal 2011[19] – CQO[D]					■■⌘											
		Goelman 1988[31]					★											
		Herrera 2005[35]					★											
		Howes 1995[38] – African American[O]					○											
		Howes 1995[38] – European American[O]					○											
		Lyon 1995[46]					●											

Table H. ECERS Predicting all Language Outcomes M to S

		McCartney 1982[49,C]					■★					■ ★						
		Schliecker 1991[67] – Whole Sample					■❖											
		Schliecker 1991[67] – One Parent					■❖											
		Schliecker 1991[67] – Two Parent					■❖											
	**Total Score (Parents and Staff Subscale not included)**	Bryant 2003[13] – Whole Sample					■											
		Burchinal, Roberts 2000[16,T]															★	★
		McCartney 1984[50,C]					○★					○ ★						
		Peisner-Feinberg 1997[55,D]					★											
	**ECERS – Mean of 5 Items**	Peisner-Feinberg 1999[56,D]					★🡻											
**ECERS- R**	**Total Score**	Abreu-Lima 2013[3]				■ ★	■★											
		Assel 2008[9]					■											
		Barnett 2007[11] – Whole Sample					■	■						■				
		Barnett 2007[11] – Spanish					■	■						■				
		Burchinal 2011[19] - NCEDL[A]			⌘		■■⌘											
		Burchinal 2011[19] – FACES 1997[J]					▰■⌘											
		Burchinal 2011[19] – FACES 2000[K]					■■⌘											
		Fiorentino 2004[30]	●				●											
		Henry 2003[33,F]					■											

The Relationship between the Early Childhood Environment Rating Scale and its Revised Form and Child Outcomes: a Systematic Review and Meta-Analysis

Table H. ECERS Predicting all Language Outcomes M to S

		Henry 2005[34,F]					■											
		Jeon 2010[41]					○★											
		Sabol 2013[65,A]		○ ★			○ ★											
		Sabol 2014[66,N]								■■ ■■■ ■■■ ★								
		Sylva 2006[69,E]							■									
		Weiland 2013[70]					■■■											
	**ECERS-R – Total Score (Parents and Staff Subscale not included)**	Aboud 2011[2,V]											■					
		Dang 2011[23] – NCEDL[A]					■											
		Dang 2011[23] – EHS 2001-2003[H]					■											
		Keys 2013[42] – NCEDL[A]					■											
		Keys 2013[42] – EHS 01/03[H]					■											
		Le 2015[45,Z]					■■■■											
		Mashburn, Pianta 2008[48,A]		■			■											
		Peisner-Feinberg 2006[57, YA, YC]					■											
		Peisner-Feinberg 2007[58,YC]					■											
		Peisner-Feinberg 2008[60] NC 07/08					❖											
		Peisner-Feinberg 2008[59] NC 03/07[YB, YC]					❖											
		Reid 2013[64,A]		○			○											
		Zellman 2008[73,Z]					■											

Table H. ECERS Predicting all Language Outcomes M to S

	**ECERS – Total Score (Toileting Indicator and Parents and Staff Subscale not included)**	Chang 2007[20] – NCEDL (Multi & SWEEP)[A]								■								
		Chang 2007[20] – Spanish-Spanish Testing[A]					■											
		Chang 2007[20] – SWEEP (Spanish Children)[A]					■											
**ECERS Subscales**	**Language-Reasoning**	Burchinal 2011[19] – CQO[D]					⌘											
		Dickinson 2001[24]					★								★	★		
	**Interactions (Author Created)**	Burchinal 2011[19] – CQO[D]					⌘											
**ECERS-R Subscales**	**Activities**	Jeon 2010[41]					○★											
		Sylva 2006[69,E]							■									
	**Interactions**	Burchinal 2011[19] – NCEDL[A]					⌘											
		Burchinal 2011[19] – FACES 1997[J]					⌘											
		Burchinal 2011[19] – FACES 2000[K]					⌘											
		Jeon 2010[41]					○★											
		Sylva 2006[69,E]							■									
	**Language-Reasoning**	Burchinal 2011[19] - NCED[A]					⌘											
		Burchinal 2011[19] – FACES 1997[J]					⌘											
		Burchinal 2011[19] – FACES 2000[K]					⌘											
		Jeon 2010[41]					○★											
		Sylva 2006[69,E]							■									
		Zill 2003[74,K]					■											
		Zill 2006[75,K]					■											

The Relationship between the Early Childhood Environment Rating Scale and its Revised Form and Child Outcomes: a Systematic Review and Meta-Analysis

Table H. ECERS Predicting all Language Outcomes M to S

	**Parents and Staff**	Sylva 2006[69,E]							■									
	**Personal Care Routines**	Sylva 2006[69,E]							■									
	**Program Structure**	Jeon 2010[41]					○★											
		Sylva 2006[69,E]							■									
	**Space and Furnishings**	Sylva 2006[69,E]							■									
**ECERS Factors**	**Developmentally Appropriate Activities**	Lyon 1995[46]					●											
	**Preschool Appropriate Caregiving**	Lyon 1995[46]					●											
		Whitebook 1989[72,O]					⌘●											
**ECERS-R Factors**	**Provisions for Learning**	Auger 2014[10,U]					■											
		Dotterer 2012[25,A]			■		■											
		Early 2006[28,A]		★			★											
		Howes 2008[39,A]		■ ★			■★											
		Weiland 2013[70]					■■■											
		West 2010[71,B]					○○											
	**Teaching & Interactions**	Aikens 2010[4,B]					○○											
		Aikens 2012[6,M]					■■■■ ■											
		Auger 2014[10,U]					■											
		Burchinal 2008[18,A]		★			★											
		Dotterer 2012[25,A]			■		■											
		Early 2006[28,A]		★			★											
		Howes 2008[39,A]		■ ★			■★											
		Sabol 2013[65,A]		○			■											
		Weiland 2013[70]					■■■											
		West 2010[71,B]					○○											

The Relationship between the Early Childhood Environment Rating Scale and its Revised Form and Child Outcomes: a Systematic Review and Meta-Analysis

Table H. ECERS Predicting all Language Outcomes M to S

Legend for Table

Significant and Positive	Significant and Negative	Non-Significant	Statistic
★	*★*	★ (grey)	r - Zero Order Pearson's Correlation
**O**	*O*	O (grey)	Beta
■	*■*	■ (grey)	B (Unstandardized Coefficient)
❖	*❖*	❖ (grey)	T-Test
⌘	*⌘*	⌘ (grey)	Partial Correlation
●	*●*	● (grey)	F-Ratio
🡻	*🡻*	🡻 (grey)	Effect Size

[a]This paper is one of a series of Meta-Analyses and Systematic Reviews assessing the relationship between child care quality and children's outcomes; therefore, superscript letters below are in reference to various large databases that samples in these papers were drawn from. These letters have been kept consistent across the series for our readers.

[b]Samples within papers are described in more detail in S3.

[c]Acronyms for child outcomes are listed in S4.

[A]National Center for Early Development and Learning Dataset (NCEDL, 2002, 2004); [B]Head Start Family and Children Experiences Survey (FACES, 2006) Cohort; [C]Bermuda Preschool Study (1980); [D]Cost, Quality and Outcomes Study (CQO, 1993-1994); [E]Effective Preschool and Primary Education Study (EPPE, 1997-1998); [F]Georgia Early Childhood Study (GECS, 2002); [H]Early Head Start (EHS, 2001-2003 Cohort); [J]Head Start Family and Children Experiences Survey (FACES, 1997) Cohort; [K]Head Start Family and Children Experiences Survey (FACES, 2000) Cohort; [M]Head Start Family and Children Experiences Survey (FACES, 2009) Cohort; [N]Early Childhood Longitudinal Study (ECLS-B, 2001-2006, Birth Cohort); [O]National Child Care Staffing Study (NCCSS, 1988); [T]Otitis Media Study (Year NR); Preschool Curriculum Evaluation Research (PCER, 1999-2003); [V]Five Rural Districts of Bangladesh (2006-2008); (2002-2003) Cohort; [YA]More is Four North Carolina Study (2002-2003) Cohort; [YB]More is Four North Carolina Study (2003-2004) Cohort; [YC]More is Four North Carolina Study (2005-2006) Cohort; [Z]Colorado QRIS.

Supplemental Information 5
Systematic Review Results: All Outcomes

Table I. ECERS Predicting all Language Outcomes S to V

ECERS Measurement Level		STUDY[a,b]	LANGUAGE[b]										
			Story and Print Concepts[e]	Story and Print Concepts - Book Knowledge	Story and Print Concepts - Print Awareness	Story and Print Concepts - Story Comprehension	Teacher Rating of Oral Language and Literacy	Test for Early Language Development	Test of Early Reading Ability - Alphabet & Convention	Test of Early Reading Ability - Reading Quotient	TOPEL - Phonological Awareness	TOPEL - Print Knowledge	Vineland Adaptive Behavior Scales - Communication
ECERS	Total Score	Bryant 1994[12] – NonSuspect Homes											■ (grey)
		Bryant 1994[12] – Suspect Homes											■ (grey)
		Herrera 2005[35]											★
		Kontos 1991[43]						★ ○ (grey)					
	ECERS - Total Score (Parents Staff Subscale not included)	Bryant 2003[13] - Whole Sample		■	■	■ (grey)							
		Bryant 2003[13] - Males			■								
		Bryant 2003[13] - Females			■ (grey)								
ECERS-R	Total Score	Abreu-Lima 2013[3]	■★										
		Assel 2008[9]							■ (grey)				
		Henry 2003[33,F]	○										
		Henry 2005[34,F]	○ (grey)										
		Jackson 2006[40]					■ (grey)			■			

The Relationship between the Early Childhood Environment Rating Scale and its Revised Form and Child Outcomes: a Systematic Review and Meta-Analysis

Table I. ECERS Predicting all Language Outcomes S to V

		Pinto 2013[63] (Portuguese ver.)	■★										■★
	**ECERS-R – Total Score (Parents Staff Subscale not included)**	Mashburn 2008[47,F]	■										
		Peisner-Feinberg 2006[57,YA, YC]	■										
		Peisner-Feinberg 2007[58,YC]	■										
		Peisner-Feinberg 2008[59] NC 03/07 [YB, YC]	❖										
		Peisner-Feinberg 2008[59] NC 07/08 [YB, YC]									❖	❖	
**ECERS-R subscales**	**Language-Reasoning**	Jackson 2006[40]					■			■			
		Zill 2006[75,K]		■									

Legend for Table

Significant and Positive	Significant and Negative	Non-Significant	Statistic
★	★	★	r - Zero Order Pearson's Correlation
○	○	○	Beta
■	■	■	B (Unstandardized Coefficient)
⌘	⌘	⌘	Partial Correlation
⬇	⬇	⬇	Effect Size
❖	❖	❖	t-test

[a]This paper is one of a series of Meta-Analyses and Systematic Reviews assessing the relationship between child care quality and children's outcomes; therefore, superscript letters below are in reference to various large databases that samples in these papers were drawn from. These letters have been kept consistent across the series for our readers.
[b]Samples within papers are described in more detail in S3.
[c]Acronyms for child outcomes are listed in S4.
[F]Georgia Early Childhood Study (GECS, 2002); [K]Head Start Family and Children Experiences Survey (FACES, 2000) Cohort; [YA]More is Four North Carolina Study (2002-2003) Cohort; [YB]More is Four North Carolina Study (2003-2004) Cohort; [YC]More is Four North Carolina Study (2005-2006) Cohort; [Z]Colorado QRIS.

The Relationship between the Early Childhood Environment Rating Scale and its Revised Form and Child Outcomes: a Systematic Review and Meta-Analysis

Supplemental Information 5
Systematic Review Results: All Outcomes

Table J. ECERS Predicting all Language Outcomes W to Z

**ECERS Measurement Level**		**STUDY**[a,b]	**LANGUAGE**[c]											
			WJ - Dictation	WJ - Letter-Word ID[h]	WJ - Passage Comprehension	WJ - Picture Vocabulary	WJ - Rhyming	WJ - Sound Awareness	WJ - Spelling	WJ - Word Attack	WM - Language Survey -Broad Abilities	WPPSI-III Vocabulary	Writing (Grade 1)
**ECERS**	**Total Score**	Burchinal, Peisner 2000[15] - CQO[D]		★									
		Burchinal, Peisner 2000[15] - NC PS		★									
		Burchinal 2011 - CQO[D]		▰■⌘									
	**ECERS - Total Score (Parents and Staff Subscale not included)**	Peisner-Feinberg 1997[55,D]		★									
	**ECERS - Mean 5 Items**	Peisner-Feinberg 1999[56,D]		★🡇									
**ECERS-R**	**Total Score**	Aboud 2006[1,V]										★	
		Assel 2008[9]		■									
		Barnett 2007[11] - Whole Sample				■							
		Barnett 2007[11] - Spanish				■							
		Burchinal 2011[19] - NCEDL[A]		■■⌘									
		Burchinal 2011[19] - FACES 1997[J]		■■⌘									

Table J. ECERS Predicting all Language Outcomes W to Z

		Burchinal 2011[19] - FACES 2000[K]		■■⌘									
		Henry 2003[33,F]		■									
		Jackson 2006[40]									■		
		Peisner-Feinberg 2013[61]		■		■		■		■			
		Sabol 2013[65,A]					○★						
	ECERS-R - Total Score (Parents and Staff subscale not included)	Aboud 2011[2,V]											○
		Hindman 2010[37, J]	■										
		Le 2015[45,Z]		■■■■	■■■■								
		Mashburn, Pianta 2008[48,A]					■						
		Peisner-Feinberg 2006[57, YA, YC]					■						
		Peisner-Feinberg 2007[58, YC]					■						
		Peisner-Feinberg 2008[60] NC 07/08		❖									
		Peisner-Feinberg 2008[59] NC 03/07[YB, YC]					❖						
		Zellman 2008[73,Z]		■	■								
ECERS Subscales	Interactions (Author Created)	Burchinal 2011[19] - CQO[D]		⌘									
	Language-Reasoning	Burchinal 2011[19] - CQO[D]		⌘									
ECERS-R Subscales	Interactions	Burchinal 2011[19] - NCEDL[A]		⌘									
		Burchinal 2011[19] - FACES 1997[J]		⌘									
		Burchinal 2011[19] - FACES 2000[K]		⌘									
	Language-Reasoning	Burchinal 2011[19] - NCEDL[A]		⌘									
		Burchinal 2011[19] - FACES 1997[J]		⌘									
		Burchinal 2011[19] - FACES 2000[K]		⌘									
		Jackson 2006[40]									■		

The Relationship between the Early Childhood Environment Rating Scale and its Revised Form and Child Outcomes: a Systematic Review and Meta-Analysis

Table J. ECERS Predicting all Language Outcomes W to Z

		Zill 2003[74,K]		□									
		Zill 2006[75,K]	□	□									
**ECERS-R Factors**	**Provisions for Learning**	Auger 2014[10,U]		□									
		Dotterer 2012[25,A]					□						
		Early 2006[28,A]					☆						
		West 2010[71,B]		○ *●*									
	**Teaching & Interactions**	Aikens 2010[4,B]		○○									
		Aikens 2012[6,M]		□□□□□					■■□□				
		Auger 2014[10,U]		□									
		Dotterer 2012[25,A]					■						
		Early 2006[28,A]					★						
		Sabol 2013[65,A]					●						
		West 2010[71,B]		○○									

Legend for Table

Significant and Positive	Significant and Negative	Non-Significant	Statistic
★	*★*	☆	r - Zero Order Pearson's Correlation
●	*●*	○	Beta
■	*■*	□	B (Unstandardized Coefficient)
❖	*❖*	◇	T-Test
⌘	*⌘*	⌘	Partial Correlation
⬇	*⬇*	⇩	Effect Size

aThis paper is one of a series of Meta-Analyses and Systematic Reviews assessing the relationship between child care quality and children's outcomes; therefore, superscript letters below are in reference to various large databases that samples in these papers were drawn from. These letters have been kept consistent across the series for our readers.
bSamples within papers are described in more detail in S3.
cAcronyms for child outcomes are listed in S4.
ANational Center for Early Development and Learning Dataset (NCEDL, 2002, 2004); BHead Start Family and Children Experiences Survey (FACES, 2006) Cohort; DCost, Quality and Outcomes Study (CQO, 1993-1994); FGeorgia Early Childhood Study (GECS, 2002); JHead Start Family and Children Experiences Survey (FACES, 1997) Cohort; KHead Start Family and Children Experiences Survey (FACES, 2000) Cohort; MHead Start Family and Children Experiences Survey (FACES, 2009) Cohort; UPreschool Curriculum Evaluation Research (PCER, 1999-2003); VFive Rural Districts of Bangladesh (2006-2008); **YA**More is Four North Carolina Study (2002-2003) Cohort; **YB**More is Four North Carolina Study (2003-2004) Cohort; **YC**More is Four North Carolina Study (2005-2006) Cohort; ZColorado QRIS.

The Relationship between the Early Childhood Environment Rating Scale and its Revised Form and Child Outcomes: a Systematic Review and Meta-Analysis

References

1. Aboud F. Evaluation of an early childhood preschool program in rural Bangladesh. *Early Child Res Q*. 2006;21(1):46-60. doi:10.1016/j.ecresq.2006.01.008.
2. Aboud F, Hossain K. The impact of preprimary school on primary school achievement in Bangladesh. *Early Child Res Q*. 2011;26(2):237-246. doi:10.1016/j.ecresq.2010.07.001.
3. Abreu-Lima I, Leal T, Cadima J, Gamelas A. Predicting child outcomes from preschool quality in Portugal. *Eur J Psychol Educ*. 2013;28(2):399-420. doi:10.1007/s10212-012-0120-y.
4. Aikens N, Tarullo L, Hulsey L, Ross C, West J, Xue Y. A Year in Head Start: children, families and programs. ACF-ORPRE report. Washington, DC: U.S. Department of Health and Human Services, Administration for Children and Families, Office of Planning, Research and Evaluation; 2010. http://files.eric.ed.gov/fulltext/ED517213.pdf. Accessed July 1, 2015.
5. Hulsey L, Aikens N, Xue Y, Tarullo L, West J. ACF-OPRE report: data tables for FACES 2006: a year in Head Start report. Washington, DC: U.S. Department of Health and Human Services, Administration for Children and Families, Office of Planning, Research and Evaluation; 2010. http://www.acf.hhs.gov/sites/default/files/opre/year_data_tables.pdf.
6. Aikens N, Moiduddin E, Xue Y, Tarullo L, West J. Data tables for child outcomes and classroom quality in FACES 2009 Report. Washington, DC: U.S. Department of Health and Human Services, Administration for Children and Families, Office of Planning, Research and Evaluation; 2012. http://www.acf.hhs.gov/sites/default/files/opre/data_tables_for_child_outcomes_and_classroom_quality_in_faces_2009.pdf. Accessed July 1, 2015.
7. Moiduddin E, Aikens N, Tarullo L, West J, Xue Y. Child outcomes and classroom quality in FACES 2009. OPRE Report 2012-37a. Washington, DC: U.S. Department of Health and Human Services, Administration for Children and Families, Office of Planning, Research and Evaluation; 2012. http://eric.ed.gov/?id=ED539265.
8. Anders Y, Rossbach H, Weinert S, et al. Home and preschool learning environments and their relations to the development of early numeracy skills. *Early Child Res Q*. 2012;27(2):231-244. doi:10.1016/j.ecresq.2011.08.003.
9. Assel M, Landry S, Swank P. Are early childhood classrooms preparing children to be school ready? The circle teacher behavior rating scale. In: Justice L, Vukelic C, eds. *Achieving Excellence in Preschool Literacy Instruction*. New York, NY: Guilford Press; 2008:120-135.
10. Auger A, Farkas G, Burchinal M, Duncan G, Vandell D. Preschool center care quality effects on academic achievement: an instrumental variables analysis. *Dev Psychol*. 2014;50(12):2559-2571. doi:10.1037/a0037995.
11. Barnett W, Yarosz D, Thomas J, Jung K, Blanco D. Two-way and monolingual English immersion in preschool education: an experimental comparison. *Early Child Res Q*. 2007;22(3):277-293. doi:10.1016/j.ecresq.2007.03.003.
12. Bryant D, Burchinal M, Lau L, Sparling J. Family and classroom correlates of Head Start children’s developmental outcomes. *Early Child Res Q*. 1994;9(3-4):289-304. doi:10.1016/0885-2006(94)90011-6.

13. Bryant D, Maxwell K, Taylor K, Poe M, Peisner-Feinberg E, Bernier K. Smart Start and preschool child care quality in North Carolina: change over time and relation to children's readiness. Chapel Hill, NC: FPG Child Development Institute; 2003. http://files.eric.ed.gov/fulltext/ED473699.pdf.
14. Burchinal M, Nelson L. Family selection and child care experiences: implications for studies of child outcomes. *Early Child Res Q*. 2000;15(3):385-411. doi:10.1016/S0885-2006(00)00072-7.
15. Burchinal M, Peisner-Feinberg E, Bryant D, Clifford R. Children's social and cognitive development and child-care quality: testing for differential associations related to poverty, gender, or ethnicity. *Appl Dev Sci*. 2000;4(3):149-165. doi:10.1207/S1532480XADS0403_4.
16. Burchinal M, Roberts J, Riggins Jr R, Zeisel S, Neebe E, Bryant D. Relating quality of center-based child care to early cognitive and language development longitudinally. *Child Dev*. 2000;71(2):339-357. doi:10.1111/1467-8624.00149.
17. Burchinal M, Roberts J, Zeisel S, Hennon E, Hooper S. Social risk and protective child, parenting, and child care factors in early elementary school years. *Parenting*. 2006;6(1):79-113. doi:10.1207/s15327922par0601_4.
18. Burchinal M, Howes C, Pianta R, et al. Predicting child outcomes at the end of kindergarten from the quality of pre-kindergarten teacher–child interactions and instruction. *Appl Dev Sci*. 2008;12(3):140-153. doi:10.1080/10888690802199418.
19. Burchinal M, Kainz K, Cai Y. How well do our measures of quality predict child outcomes? A meta-analysis and coordinated analysis of data from large-scale studies of early childhood settings. In: Zaslow M, Martinez-Beck I, Tout K, Halle T, eds. *Quality Measurement in Early Childhood Settings*. Baltimore, MD: Paul H Brookes Publishing; 2011:11-31.
20. Chang F, Crawford G, Early D, et al. Spanish-speaking children's social and language development in pre-kindergarten classrooms. *Early Educ Dev*. 2007;18(2):243-269. doi:10.1080/10409280701282959.
21. Chin-Quee D, Scarr S. Lack of early child care effects on school-age children's social competence and academic achievement. *Early Dev Parent*. 1994;3(2):103-112. doi:10.1002/edp.2430030207.
22. Clawson C, Luze G. Individual experiences of children with and without disabilities in early childhood settings. *Top Early Child Spec Educ*. 2008;28(3):132-147. doi:10.1177/0271121407311482.
23. Dang T, Farkas G, Burchinal M, et al. Preschool center quality and school readiness: quality main effects and variation by demographic and child characteristics. Evanston, IL: Society for Research on Educational Effectiveness; 2011. http://eric.ed.gov/?id=ED519004.
24. Dickinson D, Tabors P, eds. *Beginning Literacy with Language: Young Children Learning at Home and School*. Baltimore, MD: Brookes Publishing; 2001.
25. Dotterer A, Burchinal M, Bryant D, Early D, Pianta R. Universal and targeted pre-kindergarten programmes: a comparison of classroom characteristics and child outcomes. *Early Child Dev Care*. 2012;183(7):931-950. doi:10.1080/03004430.2012.698388.
26. Dunn L. Proximal and distal features of day care quality and children's development. *Early Child Res Q*. 1993;8(2):167-192. doi:10.1016/S0885-2006(05)80089-4.

27. Dunn L, Beach S, Kontos S. Quality of the literacy environment in day care and children's development. *J Res Child Educ*. 1994;9(1):24-34. doi:10.1080/02568549409594950.
28. Early D, Bryant D, Pianta R, et al. Are teachers' education, major, and credentials related to classroom quality and children's academic gains in pre-kindergarten? *Early Child Res Q*. 2006;21(2):174-195. doi:10.1016/j.ecresq.2006.04.004.
29. Epstein A. *Training for Quality: Improving Early Childhood Programs through Systematic Inservice Training*. High/Scope Educational Research Foundation, Ypsilanti, MI.; 1993.
30. Fiorentino L, Howe N. Language competence, narrative ability, and school readiness in low-income preschool children. *Can J Behav Sci Can Sci Comport*. 2004;36(4):280-294. doi:10.1037/h0087237.
31. Goelman H, Pence A. Children in three types of day care: daily experiences, quality of care and developmental outcomes. *Early Child Dev Care*. 1988;33(1-4):67-76. doi:10.1080/0300443880330105.
32. Gordon R, Fujimoto K, Kaestner R, Korenman S, Abner K. An assessment of the validity of the ECERS–R with implications for measures of child care quality and relations to child development. *Dev Pscyhology*. 2013;49(1):146-160. doi:10.1037/a0027899.
33. Henry G, Henderson L, Ponder B, Gordon C, Mashburn A, Rickman D. Report of the findings from the Early Childhood Study: 2001-02. Georgia State University, School of Policy Studies; 2003:Atlanta, GA. http://eric.ed.gov/?id=ED481261.
34. Henry G, Rickman D, Ponder B, Henderson L, Mashburn A, Gordon C. The Georgia Early Childhood Study. Atlanta, GA: Georgia State University, School of Policy Studies; 2005.
35. Herrera M, Mathiesen M, Merino J, Recart I. Learning contexts for young children in Chile: process quality assessment in preschool centres. *Int J Early Years Educ*. 2005;13(1):13-27. doi:10.1080/09669760500048253.
36. Hestenes L, Kintner-Duffy V, Wang Y, et al. Comparisons among quality measures in child care settings: understanding the use of multiple measures in North Carolina's QRIS and their links to social-emotional development in preschool children. *Early Child Res Q*. 2015;30:199-214. doi:10.1016/j.ecresq.2014.06.003.
37. Hindman A, Skibbe L, Miller A, Zimmerman M. Ecological contexts and early learning: contributions of child, family, and classroom factors during Head Start, to literacy and mathematics growth through first grade. *Early Child Res Q*. 2010;25(2):235-250. doi:10.1016/j.ecresq.2009.11.003.
38. Howes C, Sakai L, Shinn M, Phillips D, Galinsky E, Whitebook M. Race, social class, and maternal working conditions as influences on children's development. *J Appl Dev Psychol*. 1995;16(1):107-124. doi:10.1016/0193-3973(95)90019-5.
39. Howes C, Burchinal M, Pianta R, et al. Ready to learn? Children's pre-academic achievement in pre-kindergarten programs. *Early Child Res Q*. 2008;23(1):27-50. doi:10.1016/j.ecresq.2007.05.002.
40. Jackson B, Larzelere R, St. Clair L, Corr M, Fichter C, Egertson H. The impact of HeadsUp! reading on early childhood educators' literacy practices and preschool children's literacy skills. *Early Child Res Q*. 2006;21(2):213-226. doi:10.1016/j.ecresq.2006.04.005.
41. Jeon H, Langill C, Peterson C, Luze G, Carta J, Atwater J. Children's individual experiences in early care and education:

relations with overall classroom quality and children's school readiness. *Early Educ Dev*. 2010;21(6):912-939. doi:10.1080/10409280903292500.
42. Keys T, Farkas G, Burchinal M, et al. Preschool center quality and school readiness: quality effects and variation by demographic and child characteristics. *Child Dev*. 2013;84(4):1171-1190. doi:10.1111/cdev.12048.
43. Kontos S. Child care quality, family background, and children's development. *Early Child Res Q*. 1991;6(2):249-262. doi:10.1016/0885-2006(91)90011-9.
44. Kwan C, Sylva K, Reeves B. Day care quality and child development in Singapore. *Early Child Dev Care*. 1998;144(1):69-77. doi:10.1080/0300443981440108.
45. Le V, Schaack D, Setodji C. Identifying baseline and ceiling thresholds within the Qualistar Early Learning Quality Rating and Improvement System. *Early Child Res Q*. 2015;30:215-226. doi:10.1016/j.ecresq.2014.03.003.
46. Lyon M, Canning P. *Atlantic Day Care Study*. Halifax: Mount Saint Vincent University; 1995.
47. Mashburn A. Quality of social and physical environments in preschools and children's development of academic, language, and literacy skills. *Appl Dev Sci*. 2008;12(3):103-127. doi:10.1080/10888690802199392.
48. Mashburn A, Pianta R, Hamre B, et al. Measures of classroom quality in prekindergarten and children's development of academic, language, and social skills. *Child Dev*. 2008;79(3):732-749. doi:10.1111/j.1467-8624.2008.01154.x.
49. McCartney K, Scarr S, Grajek S, Schwarz J. Environmental differences among day care centers and their effects on children's development. In: Zigler E, Gordon E, eds. *Day Care: Scientific and Social Policy Issues*. Boston, MA: Auburn House Publishing Company; 1982.
50. McCartney K. Effect of quality of day care environment on children's language development. *Dev Psychol*. 1984;20(2):244-260. doi:10.1037/0012-1649.20.2.244.
51. McWayne C, Fantuzzo J, McDermott P. Preschool competency in context: an investigation of the unique contribution of child competencies to early academic success. *Dev Psychol*. 2004;40(4):633-645. doi:10.1037/0012-1649.40.4.633.
52. Moller A, Forbes-Jones E, Hightower A. Classroom age composition and developmental change in 70 urban preschool classrooms. *J Educ Psychol*. 2008;100(4):741-753. doi:10.1037/a0013099.
53. Moller A, Forbes-Jones E, Hightower A, Friedman R. The developmental influence of sex composition in preschool classrooms: boys fare worse in preschool classrooms with more boys. *Early Child Res Q*. 2008;23(3):409-418. doi:10.1016/j.ecresq.2008.05.001.
54. Montes G, Hightower A, Brugger L, Moustafa E. Quality child care and socio-emotional risk factors: no evidence of diminishing returns for urban children. *Early Child Res Q*. 2005;20(3):361-372. doi:10.1016/j.ecresq.2005.07.006.
55. Peisner-Feinberg E, Burchinal M. Relations between preschool children's child-care experiences and concurrent development: the cost, quality, and outcomes study. *Merrill-Palmer Q*. 1997;43(3):451-477.
56. Peisner-Feinberg E, Burchinal M, Clifford R, et al. The children of the Cost, Quality, and Outcomes Study go to school: technical report. Chapel Hill, NC: FGP Child Development Center; 1999.

http://fpg.unc.edu/sites/fpg.unc.edu/files/resources/reports-and-policy-briefs/NCEDL_CQO_technical_report.pdf.

57. Peisner-Feinberg E, Maris C, More at Four Evaluation team. Evaluation of the North Carolina More at Four pre-kindergarten program: children's longitudinal outcomes and classroom quality in kindergarten. Chapel Hill, NC: FPG Child Development Institute; 2006. http://fpg.unc.edu/sites/fpg.unc.edu/files/resources/reports-and-policy-briefs/MAF_Yr4_pt2_full_report.pdf.
58. Peisner-Feinberg E, Schaaf J, The More at Four evaluation team. Children's outcomes & program quality in the fifth year. Evaluation of the North Carolina More at Four pre-kindergarten program, year 5 report (July 1, 2005-June 30, 2006). Chapel Hill, NC: FPG Child Development Institute, University of North Carolina; 2007. http://eric.ed.gov/?id=ED499809.
59. Peisner-Feinberg E, Schaaf J. Evaluation of the North Carolina More at Four pre-kindergarten program year 6 report (July 1, 2006-June 30, 2007): Children's longitudinal outcomes and program quality over time (2003-2007). Chapel Hill, NC: FPG Child Development Institute; 2008. http://ea.niusileadscape.org/docs/FINAL_PRODUCTS/LearningCarousel/maf_Yr6_rpt.pdf.
60. Peisner-Feinberg E, Schaaf J. Evaluation of the North Carolina More at Four pre-kindergarten program year 7 report (2007-2008): rerformance and progress in the seventh year (2007-2008). Chapel Hill, NC: FPG Child Development Institute. University of North Carolina; 2008. http://www.ncga.state.nc.us/documentsites/committees/JLEOC/Reports%20Received/Archives/2009%20Reports%20Received/More%20At%20Four%20Program%20Review/Year%207%20Performance%20and%20Progress.pdf. Accessed November 24, 2015.
61. Peisner-Feinberg E, Schaaf J, LaForett D. Children's growth and classroom experiences in Georgia's pre-k program: findings from the 2011–2012 evaluation study. Chapel Hill, NC: FPG Child Development Institute; 2013. http://files.eric.ed.gov/fulltext/ED541933.pdf.
62. Phillips D, McCartney K, Scarr S. Child-care quality and children's social development. *Dev Psychol*. 1987;23(4):537-543. doi:10.1037/0012-1649.23.4.537.
63. Pinto A, Pessanha M, Aguiar C. Effects of home environment and center-based child care quality on children's language, communication, and literacy outcomes. *Early Child Res Q*. 2013;28(1):94-101. doi:10.1016/j.ecresq.2012.07.001.
64. Reid J, Ready D. High-quality preschool: the socioeconomic composition of preschool classrooms and children's learning. *Early Educ Dev*. 2013;24(8):1082-1111. doi:10.1080/10409289.2012.757519.
65. Sabol T, Hong S, Pianta R, Burchinal M. Can rating pre-k programs predict children's learning? *Science*. 2013;341(6148):845-846. doi:10.1126/science.1233517.
66. Sabol T, Pianta R. Do standard measures of preschool quality used in statewide policy predict school readiness? *Educ Finance Policy*. 2014;9(2):116-164. doi:10.1162/EDFP_a_00127.
67. Schliecker E, White D, Jacobs E. The role of day care quality in the prediction of children's vocabulary. *Can J Behav Sci Can Sci Comport*. 1991;23(1):12-24. doi:10.1037/h0078960.
68. Seppanen P, Godin K, Metzger J, Bronson M, Cichon D. Observational study of early childhood programs. Dover, NH:

Development of Assistance Corp.; 1993:222. http://files.eric.ed.gov/fulltext/ED366469.pdf.

69. Sylva K, Siraj-Blatchford I, Taggart B, et al. Capturing quality in early childhood through environmental rating scales. *Early Child Res Q*. 2006;21(1):76-92. doi:10.1016/j.ecresq.2006.01.003.
70. Weiland C, Ulvestad K, Sachs J, Yoshikawa H. Associations between classroom quality and children's vocabulary and executive function skills in an urban public prekindergarten program. *Early Child Res Q*. 2013;28(2):199-209. doi:10.1016/j.ecresq.2012.12.002.
71. West J, Malone L, Hulsey L, Aikens N, Tarullo L. ACF-OPRE report: Head Start children go to kindergarten. Washington, DC: U.S. Department of Health and Human Services, Administration for Children and Families, Office of Planning, Research and Evaluation; 2010. http://www.acf.hhs.gov/sites/default/files/opre/hs_kindergarten.pdf.
72. Whitebook M, Howes C, Phillips D. Who cares? Child care teachers and the quality of care in America. Final report National Child Care Staffing Study. Berkely, CA: Child Care Employee Project; 1989:41-45.
73. Zellman G, Perlman M, Le V, Setodji C. Assessing the validity of the Qualistar Early Learning Quality Rating and Improvement System as a tool for improving child-care quality. Santa Monica, CA: RAND Education; 2008. http://www.rand.org/content/dam/rand/pubs/monographs/2008/RAND_MG650.pdf.
74. Zill N, Resnick G, Kim K, et al. Head Start FACES 2000: A whole-child perspective on program performance. Fourth progress report. Washington, DC: U.S. Department of Health and Human Services, Administration for Children and Families, Office of Planning, Research and Evaluation; 2003. http://eric.ed.gov/?id=ED478791.
75. Zill N, Resnick G, Kim K, et al. Head Start performance measures center Family and Child Experiences Survey (FACES 2000): technical report. Washington, DC: U.S. Department of Health and Human Services, Administration for Children and Families, Office of Planning, Research and Evaluation; 2006. http://www.acf.hhs.gov/sites/default/files/opre/tech2k_final2.pdf.
