# Supplementary material for: The relationship between the Early Childhood Environment Rating Scale and its revised form and child outcomes: A systematic review and meta-analysis
Source: PLoS One. 2017 Jun 6;12(6):e0178512. doi: 10.1371/journal.pone.0178512 (PMC5461062; doi:10.1371/journal.pone.0178512)
Supplement: S6 File — (PDF) [file pone.0178512.s006.pdf]

The Relationship between the Early Childhood Environment Rating Scale and its Revised Form and Child Outcomes: a Systematic Review and Meta-Analysis

Supplemental Information 6

**List of Acronyms Used in the Description of Studies**	
**Acronym Category**	**Full Name**
**Journals**	Applied Developmental Science (ADS)<br>Canadian Journal of Behavioral Science (CJBS)<br>Child Development (CD)<br>Developmental Psychology (DP)<br>Early Child Development & Care (ECD&C)<br>Early Childhood Research Quarterly (ECRQ)<br>Early Development & Parenting (ED&P)<br>Early Education & Development (EE&D)<br>European Journal of Psychology of Education (EJPE)<br>International Journal of Early Years Education (IJEYE)<br>Journal of Applied Developmental Psychology (JADP)<br>Journal of Early Childhood Research (JECR)<br>Journal of Educational Psychology (JEP)<br>Journal of Research in Childhood Education (JRCE)<br>Merrill-Palmer Quarterly (MPQ)<br>Parenting: Science and Practice (PS&P)<br>Topics in Early Childhood Special Education (TECSE)
**Large Sample**	Canadian Victoria Day Care Research Project<br>Cost, Quality and Outcomes Study (CQO)<br>Decisions in Preschool and School-Age Children (BiKS)<br>Early Childhood Longitudinal Study (ECLS-B)<br>Early Childhood Study (ECS)<br>Early Head Start (EHS) Research and Evaluation Project (EHSRE).<br>Educational Processes, Competence Development, and Selection<br>Effective Preschool and Primary Education Study (EPPE)<br>Georgia Early Childhood Study (GECS)<br>Head Start Family and Children Experiences Study (FACES)<br>Home School Study<br>National Center for Early Development and Learning Dataset (NCEDL)<br>Study of State-Wide Early Education Programs (SWEEP)<br>National Child Care Staffing Study (NCCSS)<br>North Carolina Head Start Partnership Study (NCHS Partnership Study)<br>North Carolina More at Four (NC More at Four)<br>North Carolina Public Preschool Evaluation (NCPPE)<br>Observational Study of Early Childhood Programs (OSECP)<br>Otitis Media Study (OMS)<br>Preschool Curriculum Evaluation Research (PCER)<br>Rochester Early Childhood Assessment Partnership (RECAP)

The Relationship between the Early Childhood Environment Rating Scale and its Revised Form and Child Outcomes: a Systematic Review and Meta-Analysis

List of Acronyms Used in the Description of Studies	
**Acronym Category**	**Full Name**
**Covariates**	Advanced Placement (AP)<br>Bachelor of Arts (BA)<br>Body Mass Index (BMI)<br>British Ability Scales II-General Conceptual Ability (BAS II-GCA)<br>Child Development Associate (CDA)<br>Classroom Assessment Scoring System (CLASS)<br>Developmentally Appropriate Beliefs (DAP)<br>Early Childhood Education (ECE)<br>Early Head Start (EHS) Research and Evaluation Project (EHSRE).<br>Early Language and Literacy Classroom Observation (ELLCO)<br>English as a Second Language Status (ESL)<br>English Immersion (EI)<br>Environment Rating Scales (ECERS)<br>Environment Rating Scales-Revised (ECERS-R)<br>Head Start (HS)<br>Home Observation of the *Measurement* of the Environment (HOME)<br>Home Screening Questionnaire (HSQ)<br>Intelligence Quotient (IQ)<br>Masters of Arts (MA)<br>More at Four (MAF)<br>National Institute for Early Education Research Index (NIEER Index)<br>Peabody Picture Vocabulary Test (PPVT)<br>Preschool Inventory (PSI)<br>Socio-Economic Status (SES)<br>Standard Deviation (SD)<br>Teacher–Child (T-C)<br>Two-Way Immersion (TWI)<br>Well-Child Check Up Received (WIC)
